# Supplementary material for: Non-Invasive Differential Diagnosis of Cervical Neoplastic Lesions by the Lipid Profile Analysis of Cervical Scrapings
Source: Metabolites. 2022 Sep 19;12(9):883. doi: 10.3390/metabo12090883 (PMC9506087; doi:10.3390/metabo12090883)
Supplement: Supplementary file 1 [file metabolites-12-00883-s001.zip › Supplementary file 1.pdf]

```
library(ropls)
library(pROC)
##Read and normalization data
data <- read.csv(filename, header=FALSE, stringsAsFactors = FALSE)
names(data) <- data[1,]
data <- data[-1,]
names(data) <- vapply(names(data), function(x) gsub("_neg.mzXML Peak area","",x),names(data)[1])
data[[1]] <- vapply(data[[1]],function(x) unlist(strsplit(x," | "))[1],data[[1]][1])
data[[1]] <- vapply(data[[1]], function(x) substr(x,3,nchar(x)),data[[1]][1])
data <- data[,- c(2:4)]
for (i in 2:ncol(data)) data[[i]]<-as.numeric(data[[i]])
samples_information <- read.csv(inf_filename, header=FALSE, stringsAsFactors = FALSE)
names(samples_information) <- samples_information[1,]
samples_information <- samples_information[-1,]
data <- data[,!grepl("qc",names(data))]
data <- data[,!grepl("target",names(data))]
group_by_day <- list()
for (i in 1:length(unique(samples_information$day))) group_by_day[[i]]<-data.frame("Lipid"=data[,1])
samples_information$day <- as.numeric(samples_information$day)
i <- 2
while ( i<= ncol(data))
{
     print(i)
     print(names(data)[i])
     sample <- unlist(strsplit(names(data)[i],"_"))[2]
    if (sum(samples_information$sample == sample)>0)
    {
          group_by_day[[samples_information[samples_information$sample==sample,"day"]]] <-
cbind(group_by_day[[samples_information[samples_information$sample==sample,"day"]]],data[[i]])
          names(group_by_day
[[samples_information[samples_information$sample==sample,"day"]]])[ncol(group_by_day[[samples_i
nformation[samples_information$sample==sample,"day"]]])] <- names(data)[i]
          i <- i+1
```

```
    }
    else data <- data[,-i]
}
sigma_common <- apply(data[,-1],1,sd)
mean_common <- apply(data[,-1],1,mean)
group_by_day_norm <- list()
for (i in 1:length(group_by_day))
{
    sigma<-apply(group_by_day[[i]][,-1],1,sd)
    mean<-apply(group_by_day[[i]][,-1],1,mean)
    group_by_day_norm[[i]] <- group_by_day[[i]]
    for (k in 1:nrow(group_by_day_norm[[i]]))
        group_by_day_norm[[i]][k,-1]<-(group_by_day[[i]][k,-1] mean[k]) / sigma[k]*sigma_common[k]
+ mean_common[k]
}
data_autoscaling <- data[,1]
for (i in 1:length(group_by_day_norm))
     data_autoscaling<-cbind(data_autoscaling,group_by_day_norm[[i]][,-1])
data_autoscaling[[1]] <- data[[1]]
sample <- names(data_autoscaling)[-1]
temp_matrix <- t(data_autoscaling[,-1])
lipid <- data_autoscaling[[1]]
case <- c()
for (i in 1:length(sample))
{
     case<-append(case, samples_information$group[samples_information$sample ==
unlist(strsplit(sample[i],"_"))[2]])
     print(samples_information$group[samples_information$sample==unlist(strsplit(sample[i],"_"))[2]])
}
data_for_analysis <- data.frame("Sample"= sample,"Case" = case)
data_for_analysis <- data.frame(cbind(data_for_analysis, temp_matrix),stringsAsFactors = FALSE)
data_for_analysis$Sample <- as.character(data_for_analysis$Sample)
data_for_analysis$Case <- as.character(data_for_analysis$Case)
```

```
names(data_for_analysis) <- c("Sample","Case",lipid)


####Manna-Whitney test selection#########
data_for_analysis [data_for_analysis  < 0] <- 0
for (i in 0:3)
{
    for (j in (i+1):4)
    {
          data_temp <- data_for_analysis[data_for_analysis$Case==i | data_for_analysis_zero$Case==j,]
          k <- 3
         Case <- data_temp$Case
         data_temp$Case <- as.factor(data_temp$Case)
         p <- c()
        while(k <= ncol(data_temp))
        {
             if (wilcox.test(data_temp[[k]] ~ data_temp$Case,paired=FALSE)$p.value<0.05)
            {
                 p <- append(p,wilcox.test(data_temp[[k]]~data_temp$Case,paired=FALSE)$p.value)
                 k <- k+1
            }
            else data_temp <- data_temp[,-k]
         }
         if (ncol(data_temp) > 2)
         {
                  if (ncol(data_temp) == 3)
                  {
                       data_for_quantile <- data_temp[,-1]
                       data_for_quantile$Case <- as.character(data_for_quantile$Case)
                       ctype <- unique(data_for_quantile$Case)
                    }
                   else
         {
```

```
            data_for_quantile <- data_temp[,-c(1,2)]
            data_for_quantile <- cbind(Case,data_for_quantile)
             data_for_quantile$Case <- as.character(data_for_quantile$Case)
            ctype<-unique(data_for_quantile$Case)
          }
      stat_inf<-list()
      for (k in 1:length(ctype))
      {
          stat_inf[[k]]<-t(apply(data.frame(data_for_quantile[data_for_quantile$Case==ctype[k],-
1]),2,function(x) quantile(x,probs=c(0.25,0.5,0.75),na.rm = FALSE)))
          names(stat_inf)[k]<-ctype[k]
      }
       p_value <- p
      lipid_stat_inf <- data.frame(Lipid=names(data_temp)[-c(1,2)])
      for (k in 1:length(ctype))
      {
           stat <- apply(stat_inf[[k]], 1, function(x) paste0(format(x[2],scientific = TRUE,digits =
2),"(",format(x[1],scientific = TRUE,digits = 2),";",format(x[3],scientific = TRUE,digits = 2),")"))
        lipid_stat_inf <- cbind(lipid_stat_inf,stat)
        names(lipid_stat_inf)[k+1] <- ctype[k]
      }
      lipid_stat_inf <- cbind(lipid_stat_inf,p_value)
      write.csv(lipid_stat_inf,path_write,row.names = FALSE)
  }
  }
}
########OPLS-based VIP calculation#######
for (i in 0:3)
{
  for (j in (i+1):4)
  {
    data_temp <- data_for_analysis [data_for_analysis$Case==i | data_for_analysis $Case==j,-1]
    msData <- data_temp[, -1]
```

```
    parametr <- temp[[1]]
    endoPos.oplsda <- opls(msData, as.factor(parametr), predI = 1, orthoI = 1, scaleC = 'pareto')
    x <- endoPos.oplsda@vipVn
    sortedVIP <- sort(x, decreasing = T)
    write.csv(sortedVIP, pathwrite)
}
}

###### Manna-Whitney set #######
lipid<- read.csv(manna_whitney_file, stringAsFactors = FALSE]
####OPLS-set feature ####
VIP<-read.csv(VIP_file, stringAsFactors = FALSE)
lipid <- VIP[[1]][VIP [[2]] >1]
#######logistic regression feature selection######
data_case <- data[data$Case == i | data$Case == j, -1]
data_case$Case[data$Case == min(data$Case)] <- 0
data_case$Case[data$Case == max(data$Case)] <- 1
full <- glm(Case~.,data=data_case,family = binomial())
nothing <- glm(Case~ 1,data=data_case,family=binomial())
forwards<- step(nothing,scope=list(lower=formula(nothing),upper=formula(full)), direction="forward",
steps = 100000,method="AIC",trace=0)
set<-as.character(vapply(row.names(summary(forwards)$coefficients)[-1],function(x)
gsub("`","",x),FUN.VALUE = row.names(summary(forwards)$coefficients)[1]))
select_data<-data_case[,c(Case, set)]
model<-glm(Case~.,data= select_data,family = binomial())
write.csv(summary(model)$coefficients,file_coefs))
write.csv(confint(model),file_confint)

#######models combination and validation#########
lipid_pos<-read.csv(lipid_pos_file,stringsAsFactors = FALSE,header = FALSE)
names(lipid_pos)<-lipid_pos[1,]
lipid_pos<-lipid_pos[-1,]
names(lipid_pos)[2]<-"Group"
```

```
for (i in 2:ncol(lipid_pos)) lipid_pos[[i]]<-as.numeric(lipid_pos[[i]])

models_pos<-list()

temp<-lipid_pos[lipid_pos$Group==0 | lipid_pos$Group==1,c("Group","Cer-NDS(d16:0/16:0)+H","CE(24:1)+NH4")]

result<-c()

true_result<-temp$Group

for (i in 1:nrow(temp))

{

  data_test<-temp[i,-1]

  data_train<-temp[-i,]

  model<-glm(Group~.,data=data_train,family = binomial())

  result<-append(result,predict(model,data_test))

}

roc_inf<-roc(true_result,result)

thre<-coords(roc_inf,"best", "threshold")$threshold

model<-glm(Group~.,data=temp,family = binomial())

models_pos[[1]]<-list(model,thre)

names(models_pos)[1]<-"0_1"

names(models_pos[[1]])<-c("model","thre")


temp<-lipid_pos[lipid_pos$Group==0 | lipid_pos$Group==2,c("Group","Cer-NDS(d16:0/16:0)+H","PEtOH(20:1_20:1)-H")]

temp$Group[temp$Group==2]<-1

result<-c()

true_result<-temp$Group

for (i in 1:nrow(temp))

{

  data_test<-temp[i,-1]

  data_train<-temp[-i,]

  model<-glm(Group~.,data=data_train,family = binomial())

  result<-append(result,predict(model,data_test))

}

roc_inf<-roc(true_result,result)
```

```
thre<-coords(roc_inf,"best", "threshold")$threshold
model<-glm(Group~.,data=temp,family = binomial())
models_pos[[2]]<-list(model,thre)
names(models_pos)[2]<-"0_2"
names(models_pos[[2]])<-c("model","thre")

temp<-lipid_pos[lipid_pos$Group==0 | lipid_pos$Group==3,c("Group","Cer-NDS(d16:0/18:0)+H")]
temp$Group[temp$Group==3]<-1
result<-c()
true_result<-temp$Group
for (i in 1:nrow(temp))
{
  data_test<-data.frame(temp[i,-1])
  names(data_test)[1]<-"Cer-NDS(d16:0/18:0)+H"
  data_train<-temp[-i,]
  model<-glm(Group~.,data=data_train,family = binomial())
  result<-append(result,predict(model,data_test))
}
roc_inf<-roc(true_result,result)
thre<-coords(roc_inf,"best", "threshold")$threshold
model<-glm(Group~.,data=temp,family = binomial())
models_pos[[3]]<-list(model,thre)
names(models_pos)[3]<-"0_3"
names(models_pos[[3]])<-c("model","thre")

temp<-lipid_pos[lipid_pos$Group==0 |
lipid_pos$Group==4,c("Group","CE(24:1)+NH4","PC(18:2_18:3)+H","TG(10:0_8:0_8:0)+NH4")]
temp$Group[temp$Group==4]<-1
result<-c()
true_result<-temp$Group
for (i in 1:nrow(temp))
{
  data_test<-temp[i,-1]
```

```
  data_train<-temp[-i,]
  model<-glm(Group~.,data=data_train,family = binomial())
  result<-append(result,predict(model,data_test))
}
roc_inf<-roc(true_result,result)
thre<-coords(roc_inf,"best", "threshold")$threshold
model<-glm(Group~.,data=temp,family = binomial())
models_pos[[4]]<-list(model,thre)
names(models_pos)[4]<-"0_4"
names(models_pos[[4]])<-c("model","thre")

temp<-lipid_pos[lipid_pos$Group==1 | lipid_pos$Group==2,c("Group","SM(d18:0/16:0)+H","PC(16:0_22:6)+H","PE(18:0_20:4)+H","SM(d18:1/22:0)+H","Plasmenyl-PE(P-16:0/22:6)+H","CE(18:1)+NH4","PC(16:1_18:0)+H","CE(24:1)+NH4")]
temp$Group[temp$Group==1]<-0
temp$Group[temp$Group==2]<-1
result<-c()
true_result<-temp$Group
for (i in 1:nrow(temp))
{
  data_test<-temp[i,-1]
  data_train<-temp[-i,]
  model<-glm(Group~.,data=data_train,family = binomial())
  result<-append(result,predict(model,data_test))
}
roc_inf<-roc(true_result,result)
thre<-coords(roc_inf,"best", "threshold")$threshold
model<-glm(Group~.,data=temp,family = binomial())
models_pos[[5]]<-list(model,thre)
names(models_pos)[5]<-"1_2"
names(models_pos[[5]])<-c("model","thre")

temp<-lipid_pos[lipid_pos$Group==1 | lipid_pos$Group==3,c("Group","CE(24:1)+NH4")]
```

```
temp$Group[temp$Group==1]<-0
temp$Group[temp$Group==3]<-1
result<-c()
true_result<-temp$Group
for (i in 1:nrow(temp))
{
  data_test<-data.frame(temp[i,-1])
  names(data_test)[1]<-"CE(24:1)+NH4"
  data_train<-temp[-i,]
  model<-glm(Group~.,data=data_train,family = binomial())
  result<-append(result,predict(model,data_test))
}
roc_inf<-roc(true_result,result)
thre<-coords(roc_inf,"best", "threshold")$threshold
model<-glm(Group~.,data=temp,family = binomial())
models_pos[[6]]<-list(model,thre)
names(models_pos)[6]<-"1_3"
names(models_pos[[6]])<-c("model","thre")


temp<-lipid_pos[lipid_pos$Group==1 |
lipid_pos$Group==4,c("Group","CE(18:1)+NH4","MG(18:0)+H","PC(16:0_16:0)+H","TG(14:0_16:0_16:1)+
NH4","LPC(22:3)+Na","TG(16:0_16:0_18:0)+NH4")]
temp$Group[temp$Group==1]<-0
temp$Group[temp$Group==4]<-1
result<-c()
true_result<-temp$Group
for (i in 1:nrow(temp))
{
  data_test<-temp[i,-1]
  data_train<-temp[-i,]
  model<-glm(Group~.,data=data_train,family = binomial())
  result<-append(result,predict(model,data_test))
}
```

```
roc_inf<-roc(true_result,result)
thre<-coords(roc_inf,"best", "threshold")$threshold
model<-glm(Group~.,data=temp,family = binomial())
models_pos[[7]]<-list(model,thre)
names(models_pos)[7]<-"1_4"
names(models_pos[[7]])<-c("model","thre")

temp<-lipid_pos[lipid_pos$Group==2 |
lipid_pos$Group==3,c("Group","PC(16:0_22:6)+H","PC(14:0_16:0)+H")]
temp$Group[temp$Group==2]<-0
temp$Group[temp$Group==3]<-1
result<-c()
true_result<-temp$Group
for (i in 1:nrow(temp))
{
  data_test<-temp[i,-1]
  data_train<-temp[-i,]
  model<-glm(Group~.,data=data_train,family = binomial())
  result<-append(result,predict(model,data_test))
}
roc_inf<-roc(true_result,result)
thre<-coords(roc_inf,"best", "threshold")$threshold
model<-glm(Group~.,data=temp,family = binomial())
models_pos[[8]]<-list(model,thre)
names(models_pos)[8]<-"2_3"
names(models_pos[[8]])<-c("model","thre")

temp<-lipid_pos[lipid_pos$Group==2 |
lipid_pos$Group==4,c("Group","PC(16:0_18:1)+H","SM(d20:0/22:0)+H","CerP(d18:0/22:0)+H","LPC(22:3
)+Na","PC(16:0_20:5)+H","plasmanyl-TG(O-18:1_16:0_18:1)+NH4")]
temp$Group[temp$Group==2]<-0
temp$Group[temp$Group==4]<-1
result<-c()
```

```
true_result<-temp$Group
for (i in 1:nrow(temp))
{
  data_test<-temp[i,-1]
  data_train<-temp[-i,]
  model<-glm(Group~.,data=data_train,family = binomial())
  result<-append(result,predict(model,data_test))
}
roc_inf<-roc(true_result,result)
thre<-coords(roc_inf,"best", "threshold")$threshold
model<-glm(Group~.,data=temp,family = binomial())
models_pos[[9]]<-list(model,thre)
names(models_pos)[9]<-"2_4"
names(models_pos[[9]])<-c("model","thre")

temp<-lipid_pos[lipid_pos$Group==3 | lipid_pos$Group==4,c("Group","CE(24:1)+NH4","Plasmenyl-PE(P-16:0/20:4)+H","Plasmanyl-LPC(O-16:0)+H")]
temp$Group[temp$Group==3]<-0
temp$Group[temp$Group==4]<-1
result<-c()
true_result<-temp$Group
for (i in 1:nrow(temp))
{
  data_test<-temp[i,-1]
  data_train<-temp[-i,]
  model<-glm(Group~.,data=data_train,family = binomial())
  result<-append(result,predict(model,data_test))
}
roc_inf<-roc(true_result,result)
thre<-coords(roc_inf,"best", "threshold")$threshold
model<-glm(Group~.,data=temp,family = binomial())
models_pos[[10]]<-list(model,thre)
names(models_pos)[10]<-"3_4"
```

```
names(models_pos[[10]])<-c("model","thre")


lipid_neg<-read.csv(lipid_neg_file,stringsAsFactors = FALSE,header = FALSE)
names(lipid_neg)<-lipid_neg[1,]
lipid_neg<-lipid_neg[-1,]
names(lipid_neg)[2]<-"Group"
for (i in 2:ncol(lipid_neg)) lipid_neg[[i]]<-as.numeric(lipid_neg[[i]])
models_neg<-list()
temp<-lipid_neg[lipid_neg$Group==0 | lipid_neg$Group==1,c("Group","LPC(18:2)+HCO2","LPC(18:1)+HCO2","Plasmenyl-PE(P-16:0/22:6)-H")]
result<-c()
true_result<-temp$Group
for (i in 1:nrow(temp))
{
  data_test<-temp[i,-1]
  data_train<-temp[-i,]
  model<-glm(Group~.,data=data_train,family = binomial())
  result<-append(result,predict(model,data_test))
}
roc_inf<-roc(true_result,result)
thre<-coords(roc_inf,"best", "threshold",as.list=FALSE, best.method = "closest.topleft")$threshold
model<-glm(Group~.,data=temp,family = binomial())
models_neg[[1]]<-list(model,thre)
names(models_neg)[1]<-"0_1"
names(models_neg[[1]])<-c("model","thre")

temp<-lipid_neg[lipid_neg$Group==0 | lipid_neg$Group==2,c("Group","LPC(18:2)+HCO2","LPC(18:1)+HCO2","PC(16:0_18:2)+HCO2","PC(16:0_18:1)+HCO2")]
temp$Group[temp$Group==2]<-1
result<-c()
true_result<-temp$Group
```

```
for (i in 1:nrow(temp))
{
  data_test<-temp[i,-1]
  data_train<-temp[-i,]
  model<-glm(Group~.,data=data_train,family = binomial())
  result<-append(result,predict(model,data_test))
}
roc_inf<-roc(true_result,result)
thre<-coords(roc_inf,"best", "threshold",as.list=FALSE, best.method = "closest.topleft")$threshold
model<-glm(Group~.,data=temp,family = binomial())
models_neg[[2]]<-list(model,thre)
names(models_neg)[2]<-"0_2"
names(models_neg[[2]])<-c("model","thre")


temp<-lipid_neg[lipid_neg$Group==0 | lipid_neg$Group==3,c("Group","Plasmenyl-PE(P-16:0/16:1)-H","OxPC(18:1_16:1(OH))+HCO2","PC(18:2_18:2)+HCO2","OxPC(16:0_14:1(COOH))+HCO2","PC(16:0_22:6)+HCO2")]
temp$Group[temp$Group==3]<-1
result<-c()
true_result<-temp$Group
for (i in 1:nrow(temp))
{
  data_test<-temp[i,-1]
  data_train<-temp[-i,]
  model<-glm(Group~.,data=data_train,family = binomial())
  result<-append(result,predict(model,data_test))
}
roc_inf<-roc(true_result,result)
thre<-coords(roc_inf,"best", "threshold",as.list=FALSE, best.method = "closest.topleft")$threshold
model<-glm(Group~.,data=temp,family = binomial())
models_neg[[3]]<-list(model,thre)
names(models_neg)[3]<-"0_3"
names(models_neg[[3]])<-c("model","thre")
```

```
temp<-lipid_neg[lipid_neg$Group==0 | lipid_neg$Group==4,c("Group","Plasmanyl-PC(O-16:0/16:0)+HCO2","CL(16:0_16:0_16:1_18:1)-2H","SM(d16:0/18:2)+HCO2","PC(16:0_22:6)+HCO2","PC(16:0_18:2)+HCO2")]

temp$Group[temp$Group==4]<-1

result<-c()

true_result<-temp$Group

for (i in 1:nrow(temp))

{

  data_test<-temp[i,-1]

  data_train<-temp[-i,]

  model<-glm(Group~.,data=data_train,family = binomial())

  result<-append(result,predict(model,data_test))

}

roc_inf<-roc(true_result,result)

thre<-coords(roc_inf,"best", "threshold",as.list=FALSE, best.method = "closest.topleft")$threshold

model<-glm(Group~.,data=temp,family = binomial())

models_neg[[4]]<-list(model,thre)

names(models_neg)[4]<-"0_4"

names(models_neg[[4]])<-c("model","thre")


temp<-lipid_neg[lipid_neg$Group==1 | lipid_neg$Group==2,c("Group","Plasmenyl-PE(P-18:0/22:6)-H","Cer-NP(t18:1/24:1)+HCO2","Cer-NDS(d20:0/26:0)+HCO2","OxCL(16:0_16:0_16:1(OOH)_18:1)-2H","SM(d16:0/18:1)+HCO2")]

temp$Group[temp$Group==1]<-0

temp$Group[temp$Group==2]<-1

result<-c()

true_result<-temp$Group

for (i in 1:nrow(temp))

{

  data_test<-temp[i,-1]

  data_train<-temp[-i,]

  model<-glm(Group~.,data=data_train,family = binomial())

  result<-append(result,predict(model,data_test))
```

```
}
roc_inf<-roc(true_result,result)
thre<-coords(roc_inf,"best", "threshold",as.list=FALSE, best.method = "closest.topleft")$threshold
model<-glm(Group~.,data=temp,family = binomial())
models_neg[[5]]<-list(model,thre)
names(models_neg)[5]<-"1_2"
names(models_neg[[5]])<-c("model","thre")


temp<-lipid_neg[lipid_neg$Group==1 |
lipid_neg$Group==3,c("Group","PC(16:0_18:2)+HCO2","PC(18:1_20:0)+HCO2","SM(d24:0/18:1)+HCO2",
"OxCL(22:6_22:6_22:6(OOH)_22:6(OOH))-2H","Plasmenyl-PE(P-16:0/16:1)-
H","PC(18:1_18:1)+HCO2","OxPG(18:0_20:3(2O))-H","OxPC(16:0_14:0(CHO))+HCO2","HexCer-
AP(t20:2/24:0)+HCO2","SM(d26:0/18:1)+HCO2")]
temp$Group[temp$Group==1]<-0
temp$Group[temp$Group==3]<-1
result<-c()
true_result<-temp$Group
for (i in 1:nrow(temp))
{
  data_test<-temp[i,-1]
  data_train<-temp[-i,]
  model<-glm(Group~.,data=data_train,family = binomial())
  result<-append(result,predict(model,data_test))
}
roc_inf<-roc(true_result,result)
thre<-coords(roc_inf,"best", "threshold",as.list=FALSE, best.method = "closest.topleft")$threshold
model<-glm(Group~.,data=temp,family = binomial())
models_neg[[6]]<-list(model,thre)
names(models_neg)[6]<-"1_3"
names(models_neg[[6]])<-c("model","thre")


temp<-lipid_neg[lipid_neg$Group==1 |
lipid_neg$Group==4,c("Group","SM(d22:0/20:3)+HCO2","OxCL(18:1_18:2_18:3(OOH)2_20:4)-
2H","SM(d24:1/18:1)+HCO2","PC(18:1_18:2)+HCO2","SM(d22:1/20:0)+HCO2","SM(d20:0/14:0)+HCO2")
]
```

```
temp$Group[temp$Group==1]<-0

temp$Group[temp$Group==4]<-1

result<-c()

true_result<-temp$Group

for (i in 1:nrow(temp))

{

  data_test<-temp[i,-1]

  data_train<-temp[-i,]

  model<-glm(Group~.,data=data_train,family = binomial())

  result<-append(result,predict(model,data_test))

}

roc_inf<-roc(true_result,result)

thre<-coords(roc_inf,"best", "threshold",as.list=FALSE, best.method = "closest.topleft")$threshold

model<-glm(Group~.,data=temp,family = binomial())

models_neg[[7]]<-list(model,thre)

names(models_neg)[7]<-"1_4"

names(models_neg[[7]])<-c("model","thre")


temp<-lipid_neg[lipid_neg$Group==2 |
lipid_neh$Group==3,c("Group","OxCL(22:6_22:6_22:6(OOH)_22:6(OOH))-2H","HexCer-
AP(t20:2/24:0)+HCO2")]

temp$Group[temp$Group==2]<-0

temp$Group[temp$Group==3]<-1

result<-c()

true_result<-temp$Group

for (i in 1:nrow(temp))

{

  data_test<-temp[i,-1]

  data_train<-temp[-i,]

  model<-glm(Group~.,data=data_train,family = binomial())

  result<-append(result,predict(model,data_test))

}

roc_inf<-roc(true_result,result)
```

```
thre<-coords(roc_inf,"best", "threshold",as.list=FALSE, best.method = "closest.topleft")$threshold

model<-glm(Group~.,data=temp,family = binomial())

models_neg[[8]]<-list(model,thre)

names(models_neg)[8]<-"2_3"

names(models_neg[[8]])<-c("model","thre")


temp<-lipid_neg[lipid_neg$Group==2 |
lipid_neg$Group==4,c("Group","SM(d22:0/20:3)+HCO2","SM(d22:0/18:1)+HCO2","PC(18:1_20:0)+HCO2
","SM(d24:0/18:1)+HCO2","SM(d24:0/18:0)+HCO2","SM(d24:1/18:1)+HCO2","PC(18:1_18:1)+HCO2")]

temp$Group[temp$Group==2]<-0

temp$Group[temp$Group==4]<-1

result<-c()

true_result<-temp$Group

for (i in 1:nrow(temp))

{

  data_test<-temp[i,-1]

  data_train<-temp[-i,]

  model<-glm(Group~.,data=data_train,family = binomial())

  result<-append(result,predict(model,data_test))

}

roc_inf<-roc(true_result,result)

thre<-coords(roc_inf,"best", "threshold",as.list=FALSE, best.method = "closest.topleft")$threshold

model<-glm(Group~.,data=temp,family = binomial())

models_neg[[9]]<-list(model,thre)

names(models_neg)[9]<-"2_4"

names(models_neg[[9]])<-c("model","thre")


temp<-lipid_neg[lipid_neg$Group==3 |
lipid_neg$Group==4,c("Group","SM(d16:0/18:1)+HCO2","SM(d22:0/18:1)+HCO2","SM(d22:0/20:3)+HC
O2","OxCL(18:1_18:2_20:3_20:4(OOH))-
2H","OxPC(16:0_14:1(COOH))+HCO2","SM(d20:1/16:0)+HCO2","PC(18:0_20:3)+HCO2","SM(d18:0/16:0)
+HCO2")]

temp$Group[temp$Group==3]<-0

temp$Group[temp$Group==4]<-1
```

```
result<-c()
true_result<-temp$Group
for (i in 1:nrow(temp))
{
  data_test<-temp[i,-1]
  data_train<-temp[-i,]
  model<-glm(Group~.,data=data_train,family = binomial())
  result<-append(result,predict(model,data_test))
}
roc_inf<-roc(true_result,result)
thre<-coords(roc_inf,"best", "threshold",as.list=FALSE, best.method = "closest.topleft")$threshold
model<-glm(Group~.,data=temp,family = binomial())
models_neg[[10]]<-list(model,thre)
names(models_neg)[10]<-"3_4"
names(models_neg[[10]])<-c("model","thre")

result<-c()
lipid_pos<-lipid_pos[order(lipid_pos$Sample),]
lipid_neg<-lipid_neg[order(lipid_neg$Sample),]
data_for_work_pos<-lipid_pos[,-1]
data_for_work_neg<-lipid_neg[,-1]
for (i in 1:nrow(data_for_work_pos))
{
  data_test_pos<-data_for_work_pos[i,-1]
  data_test_neg<-data_for_work_neg[i,-1]
  summ_result<-c(0,0,0,0,0)
  for (j in 1:length(models_pos))
  {
    temp_result<-predict(models_pos[[j]]$model,data_test_pos)
    if (temp_result<models_pos[[j]]$thre)
summ_result[as.numeric(unlist(strsplit(names(models_pos)[j],"_"))[1])+1]<-
summ_result[as.numeric(unlist(strsplit(names(models_pos)[j],"_")[1]))+1]+1
```

```
  else summ_result[as.numeric(unlist(strsplit(names(models_pos)[j],"_"))[2])+1]<-
summ_result[as.numeric(unlist(strsplit(names(models_pos)[j],"_"))[2])+1]+1


  temp_result<-predict(models_neg[[j]]$model,data_test_neg)
  if (temp_result<models_neg[[j]]$thre)
summ_result[as.numeric(unlist(strsplit(names(models_neg)[j],"_"))[1])+1]<-
summ_result[as.numeric(unlist(strsplit(names(models_neg)[j],"_")[1]))+1]+1
  else summ_result[as.numeric(unlist(strsplit(names(models_neg)[j],"_"))[2])+1]<-
summ_result[as.numeric(unlist(strsplit(names(models_neg)[j],"_"))[2])+1]+1
 }
 if (sum(max(summ_result)==summ_result)==1) result<-append(result,which.max(summ_result)-1)
 else
 {
  tasks<-grep(max(summ_result),summ_result)
  temp_summ_result<-rep(0,length(summ_result))
  for (j in 1:(length(tasks)-1))
  {
   for (k in (j+1):length(tasks))
   {
    name<-paste0(tasks[j]-1,"_",tasks[k]-1)
    temp_model<-models_pos[[paste0(tasks[j]-1,"_",tasks[k]-1)]]
    temp_result<-predict(temp_model$model,data_test_pos)
    if (temp_result<temp_model$thre) temp_summ_result[tasks[j]]<-temp_summ_result[tasks[j]]+1
    else temp_summ_result[tasks[k]]<-temp_summ_result[tasks[k]]+1
    temp_model<-models_neg[[paste0(tasks[j]-1,"_",tasks[k]-1)]]
    temp_result<-predict(temp_model$model,data_test_neg)
    if (temp_result<temp_model$thre) temp_summ_result[tasks[j]]<-temp_summ_result[tasks[j]]+1
    else temp_summ_result[tasks[k]]<-temp_summ_result[tasks[k]]+1
   }
  }
  result<-append(result,which.max(temp_summ_result)[1]-1)
 }
}
```

```
matrix_result<-matrix(0, nrow = 5,ncol=5)
colnames(matrix_result)=c("NILM","cervicit","LSIL","HSIL","cancer")
rownames(matrix_result)<-c("NILM","cervicit","LSIL","HSIL","cancer")
true_result<-lipid_pos$Group
for (i in 1:length(result_pos))
{
  matrix_result[true_result[i]+1,result_pos[i]+1]<-matrix_result[true_result[i]+1,result_pos[i]+1]+1
}

write.csv(matrix_result,"D:/cervical brush/expand_result_lipid.csv")
```
