# Supplementary material for: Non-Invasive Differential Diagnosis of Cervical Neoplastic Lesions by the Lipid Profile Analysis of Cervical Scrapings
Source: Metabolites. 2022 Sep 19;12(9):883. doi: 10.3390/metabo12090883 (PMC9506087; doi:10.3390/metabo12090883)
Supplement: Supplementary file 1 [file metabolites-12-00883-s001.zip › Supplementary file 2.pdf]

NILM vs cervicitis

$R^2X = 0.67$
$R^2Y = 0.26$
$Q^2Y = - 0.39$

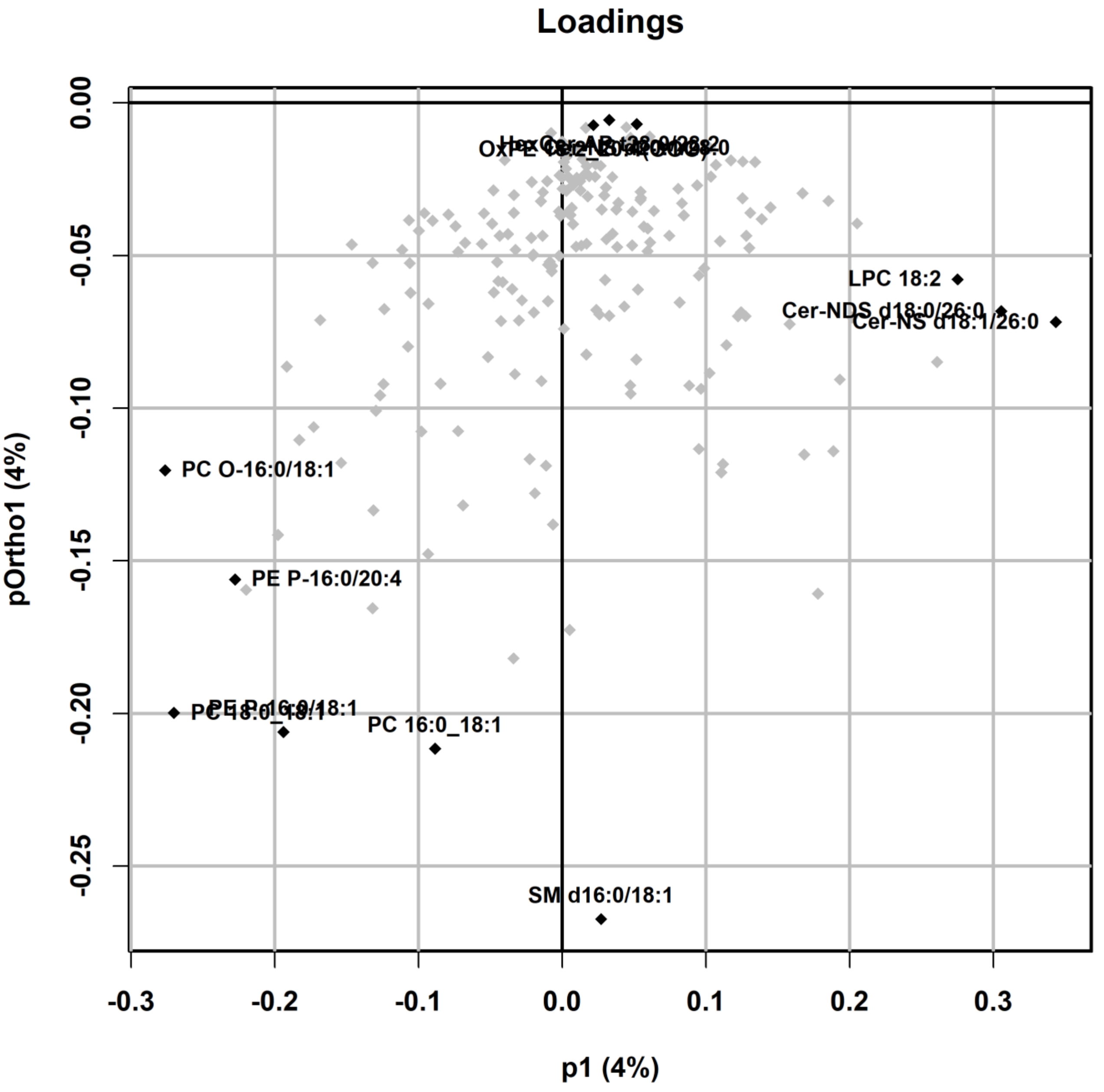

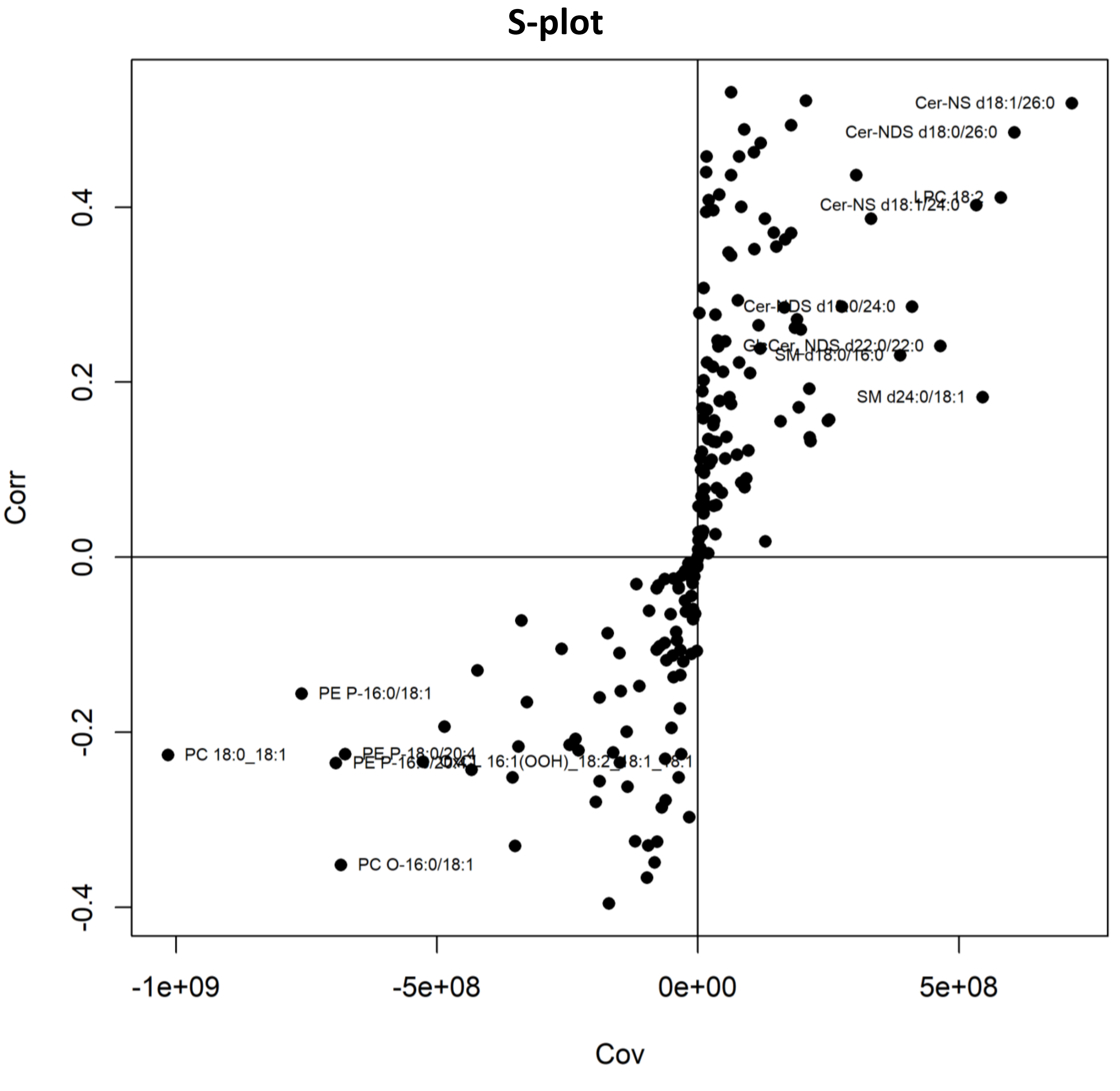
S-plot
Corr
Cov
0.4
0.2
0.0
-0.2
-0.4
-1e+09
-5e+08
0e+00
5e+08
Cer-NS d18:1/26:0
Cer-NDS d18:0/26:0
LPC 18:2
Cer-NS d18:1/24:0
SM d18:0/16:0
SM d24:0/18:1
PE P-16:0/18:1
PC 18:0_18:1
PE P-18:0/20:4
PC O-16:0/18:1

VIP-plot

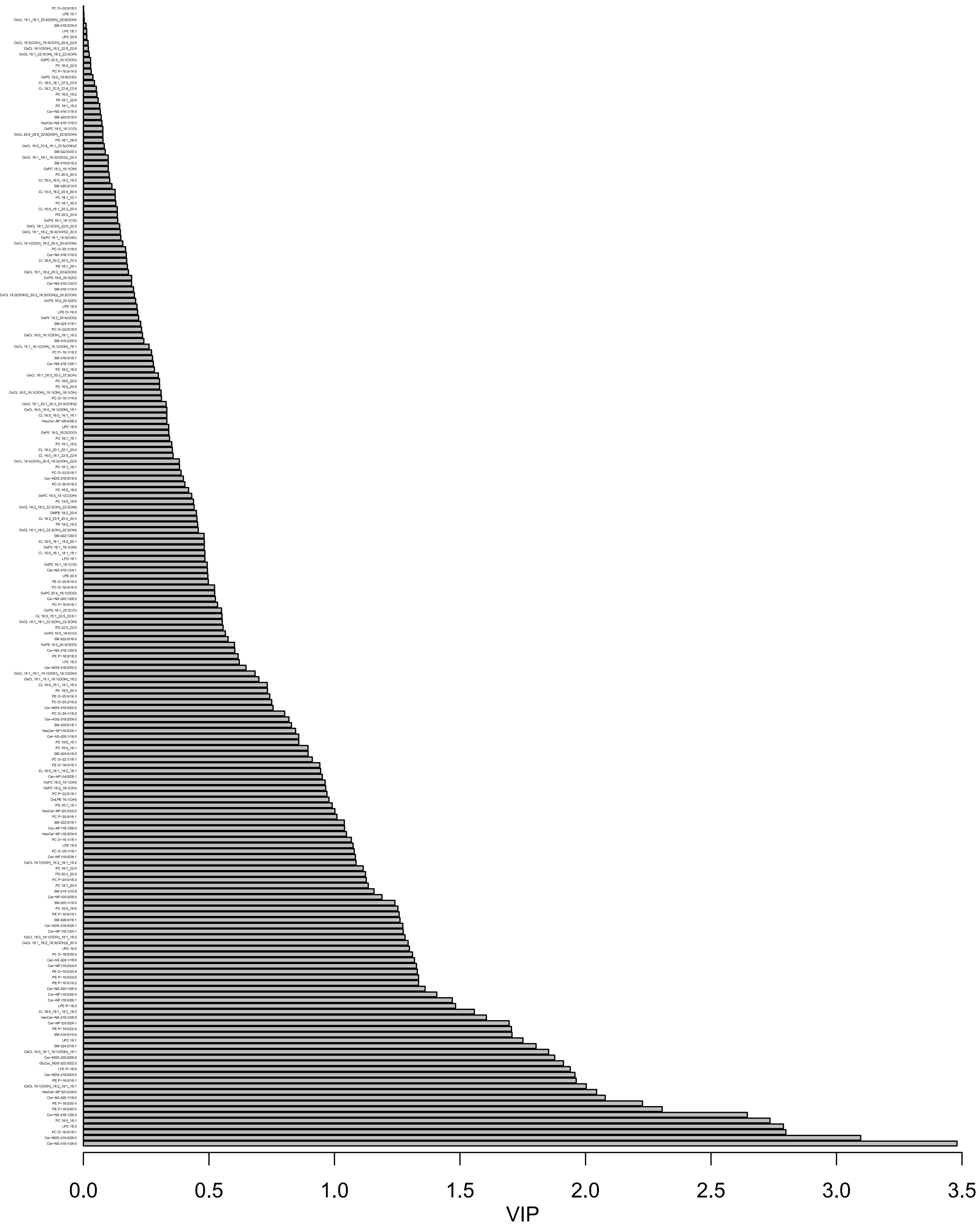

NILM vs LSIL

$R^2X = 0.74$
$R^2Y = 0.16$
$Q^2Y = - 0.36$

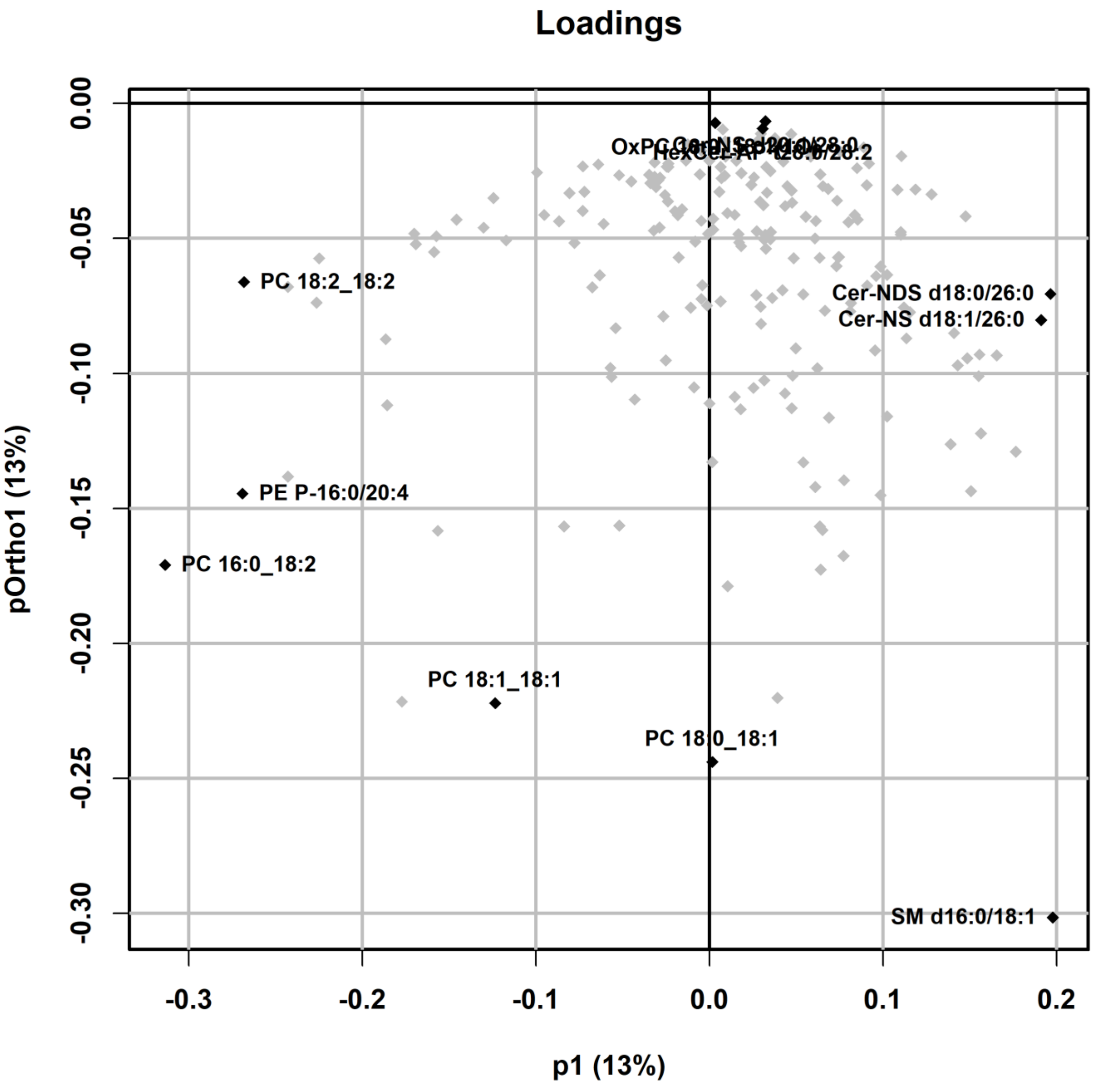

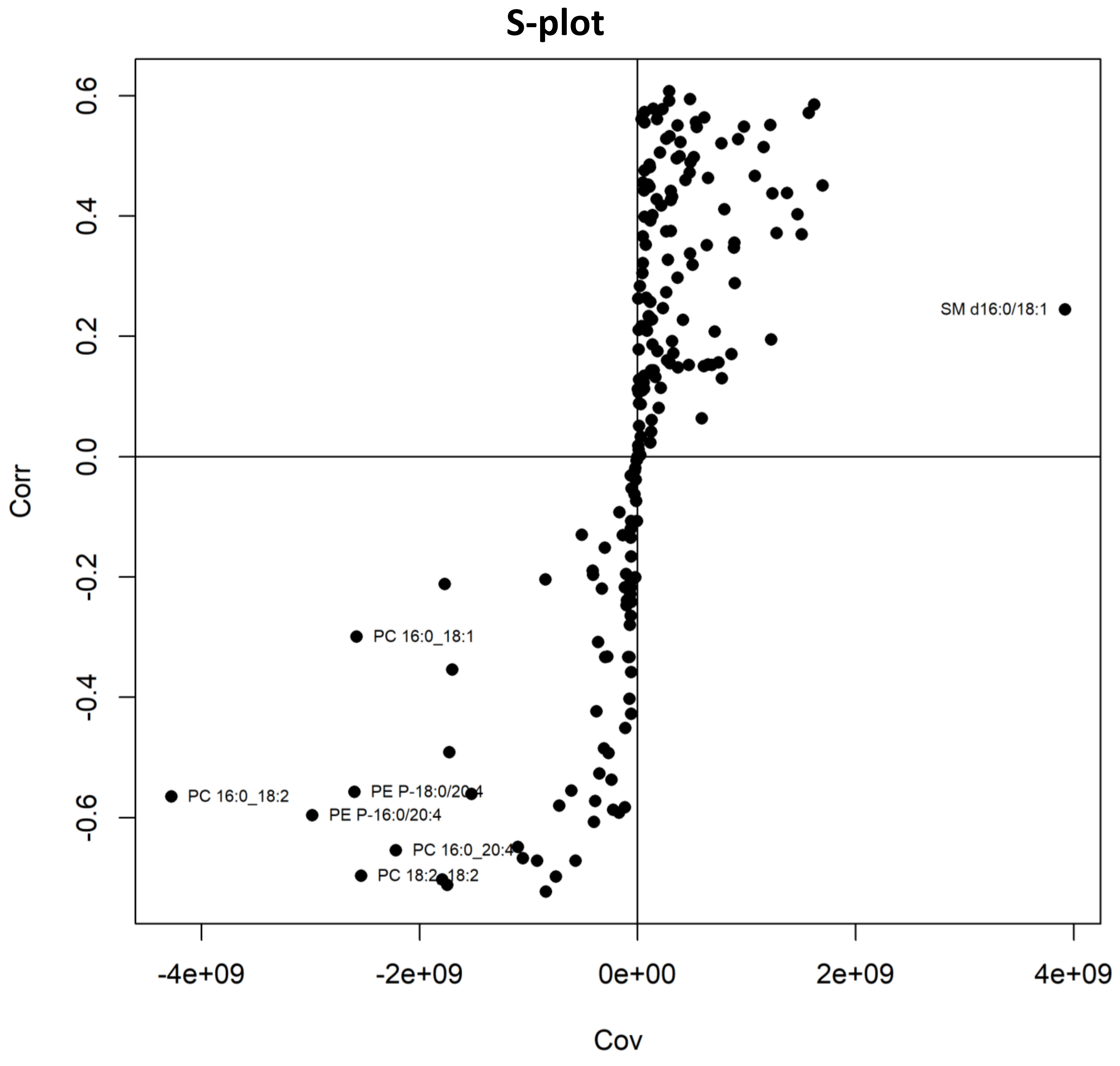
S-plot
Corr
Cov
0.6
0.4
0.2
0.0
-0.2
-0.4
-0.6
-4e+09
-2e+09
0e+00
2e+09
4e+09
SM d16:0/18:1
PC 16:0_18:1
PC 16:0_18:2
PE P-18:0/20:4
PE P-16:0/20:4
PC 16:0_20:4
PC 18:2_18:2

VIP-plot

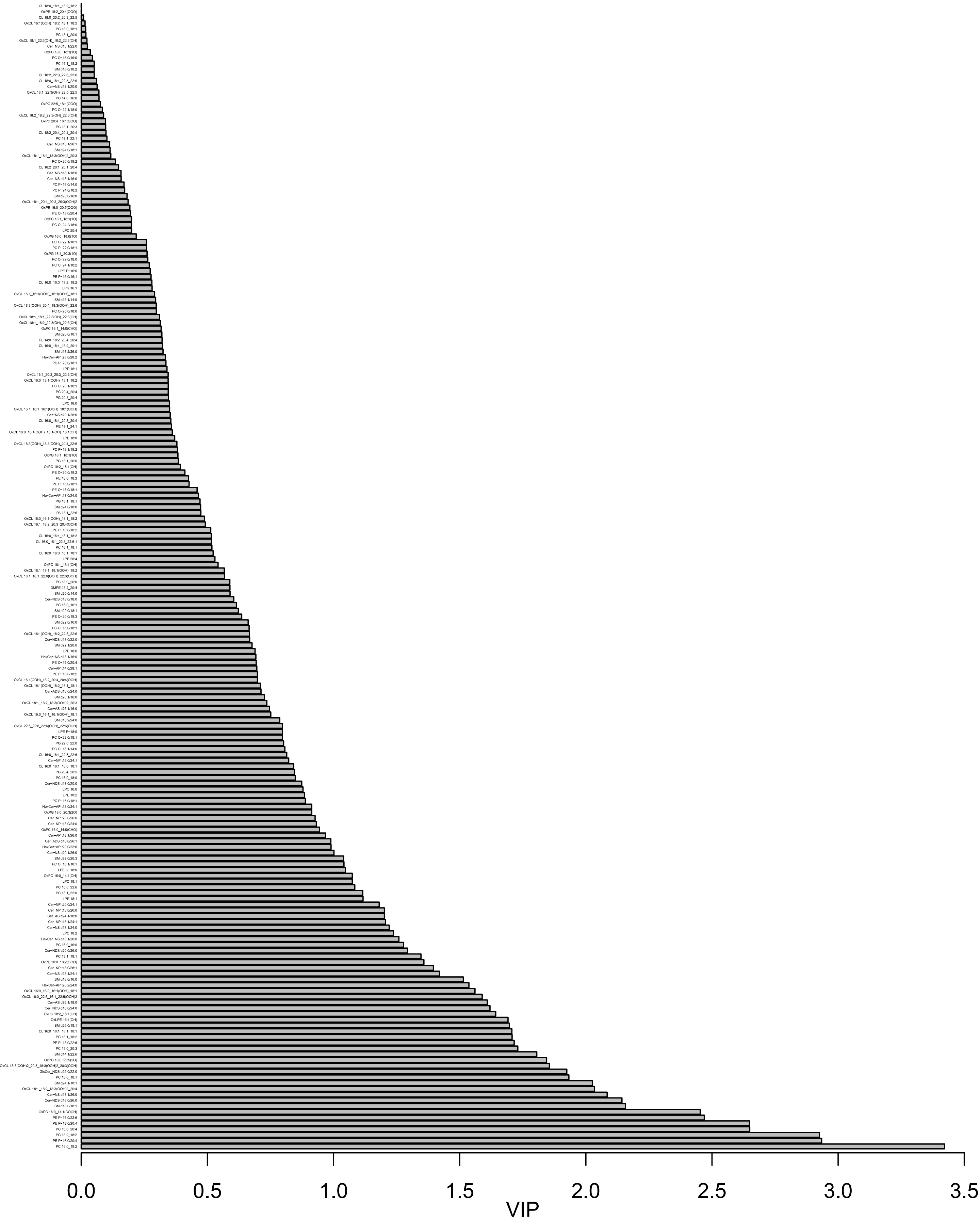

NILM vs HSIL

$R^2X = 0.72$
$R^2Y = 0.19$
$Q^2Y = 0.00$

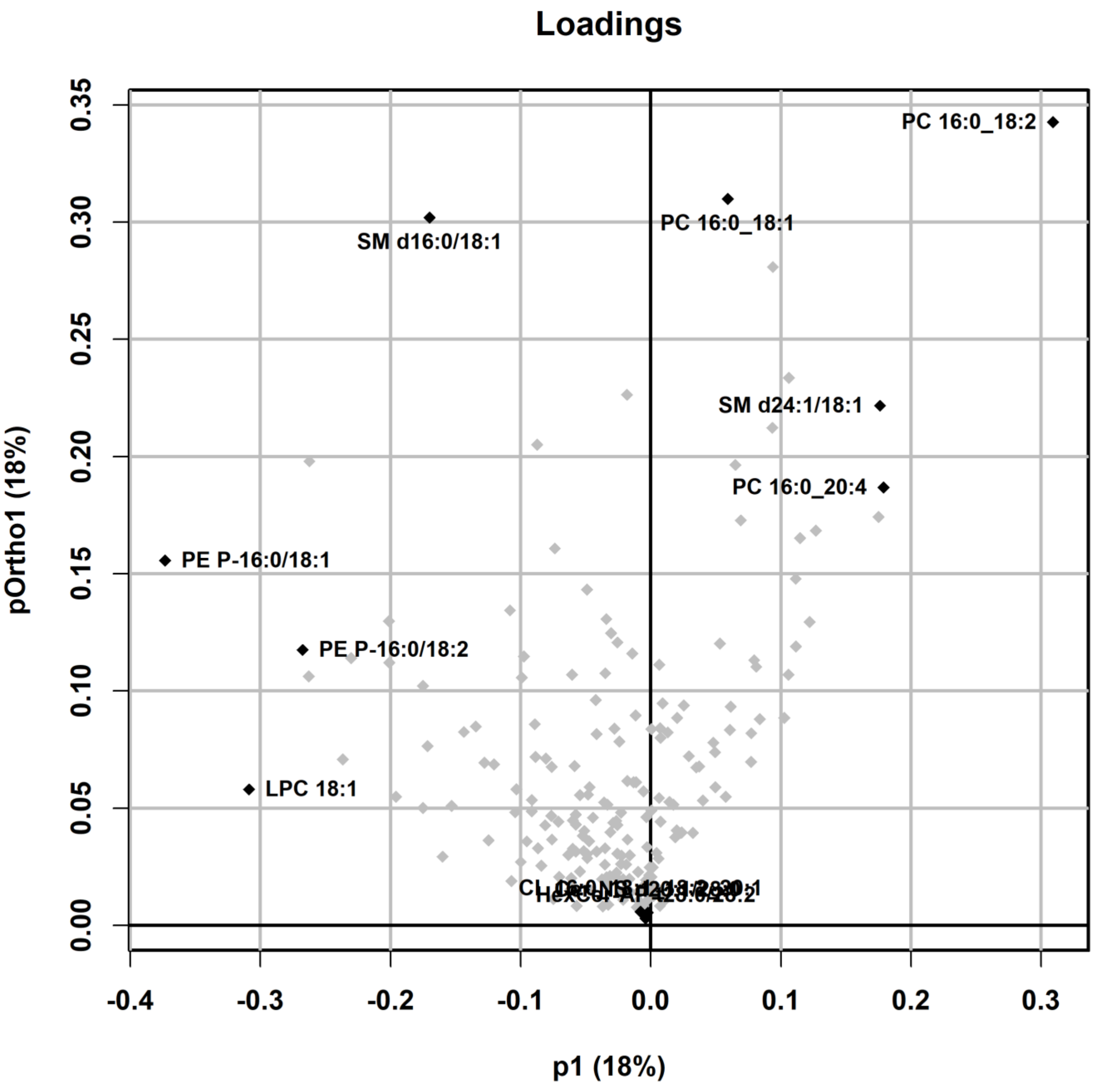

S-plot

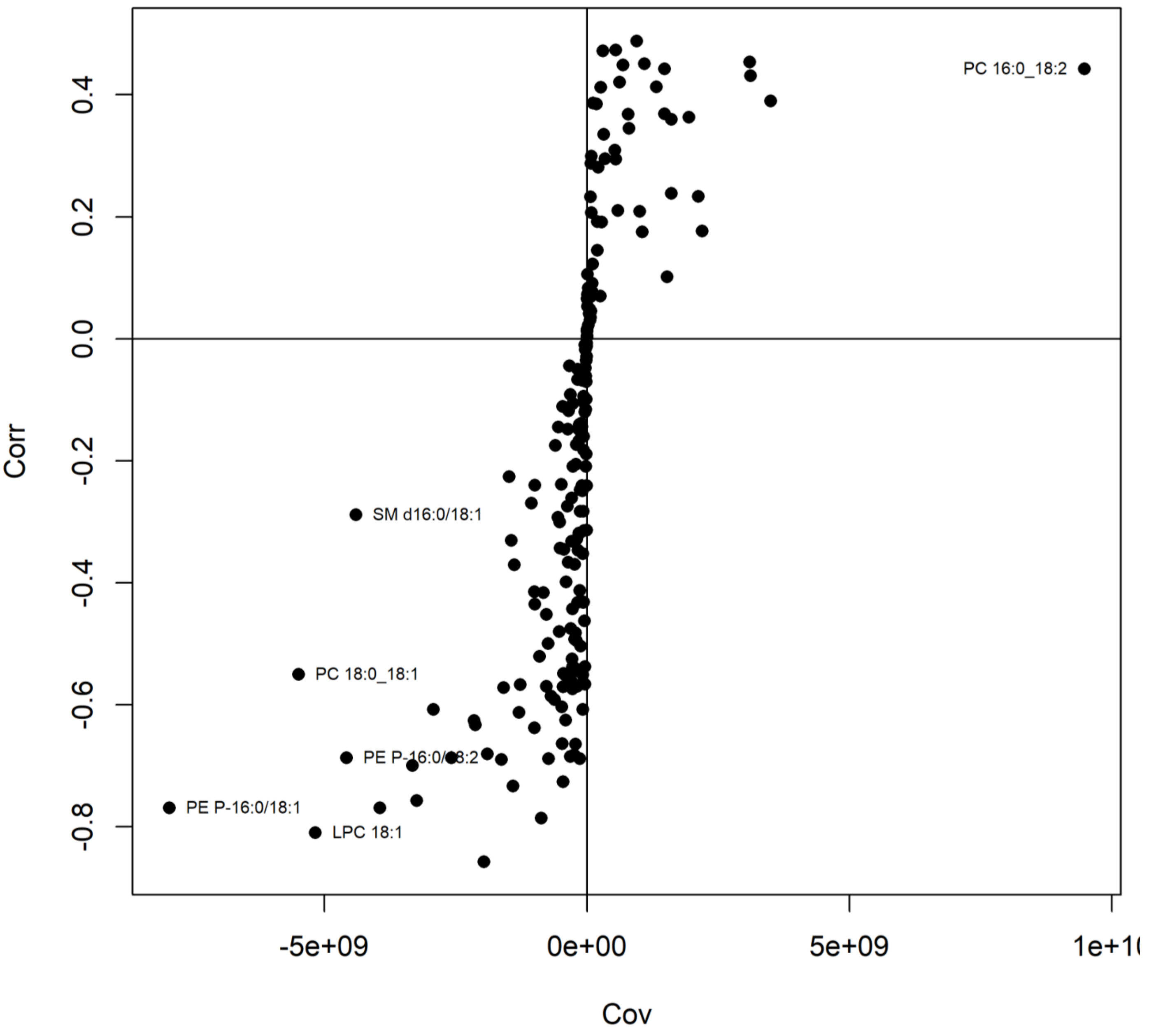
PC 16:0_18:2
SM d16:0/18:1
PC 18:0_18:1
PE P-16:0/18:2
PE P-16:0/18:1
LPC 18:1
0.4
0.2
0.0
-0.2
-0.4
-0.6
-0.8
Corr
-5e+09
0e+00
5e+09
Cov

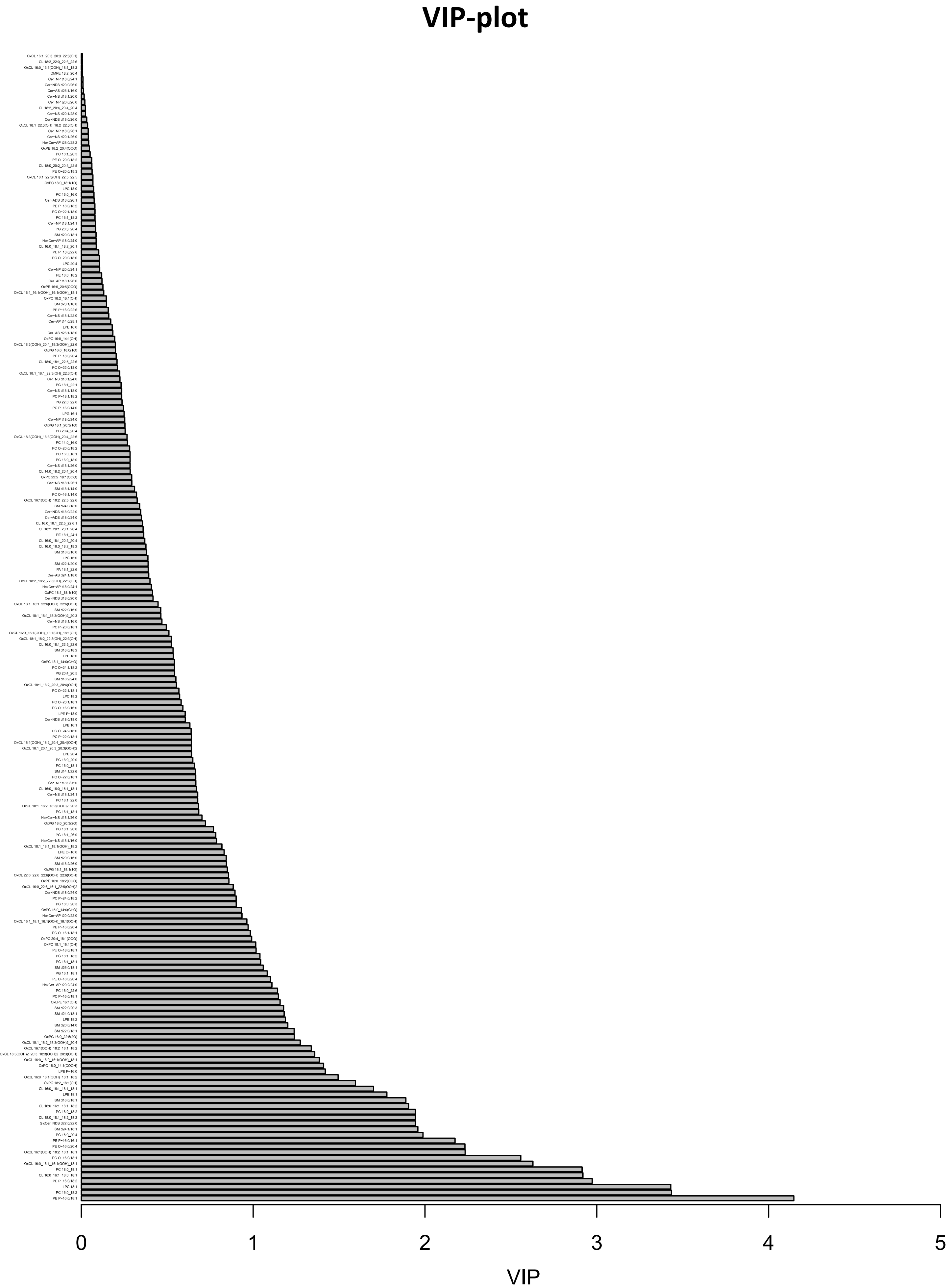
VIP-plot
0
1
2
3
4
5
VIP

NILM vs cancer

$R^2X = 0.77$
$R^2Y = 0.29$
$Q^2Y = 0.14$

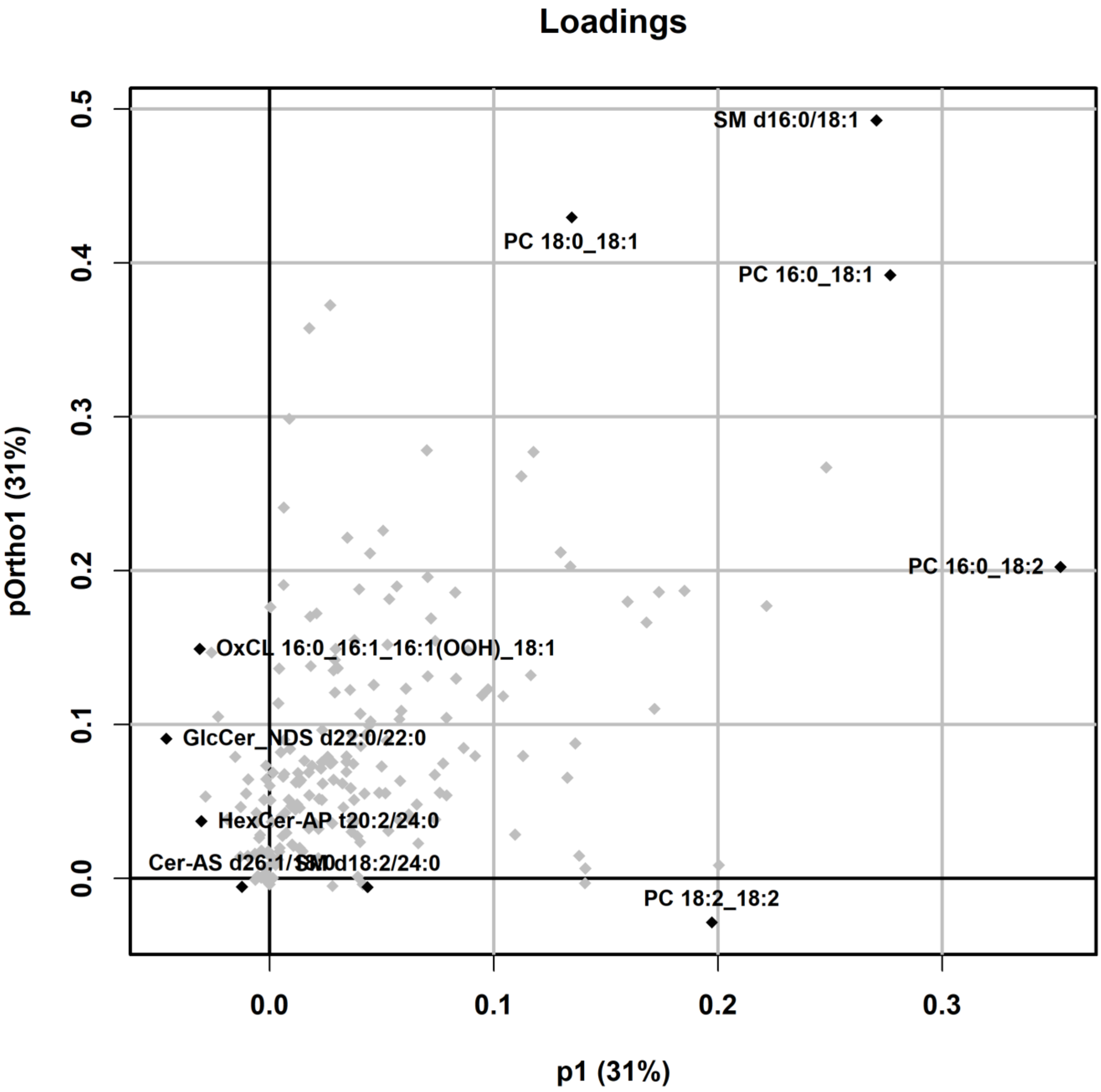

S-plot

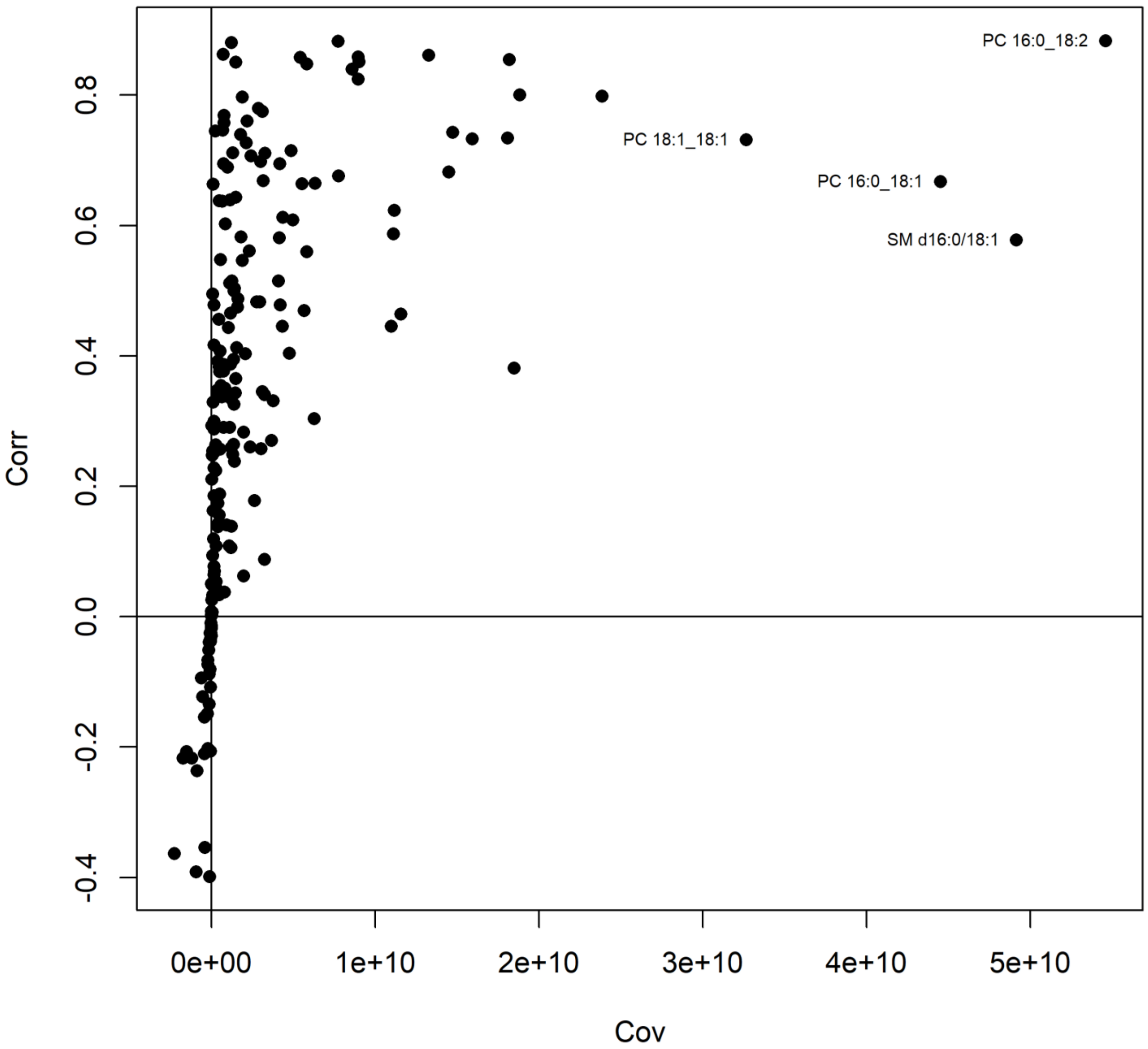
PC 16:0_18:2
PC 18:1_18:1
PC 16:0_18:1
SM d16:0/18:1
Corr
0.8
0.6
0.4
0.2
0.0
-0.2
-0.4
0e+00
1e+10
2e+10
3e+10
4e+10
5e+10
Cov

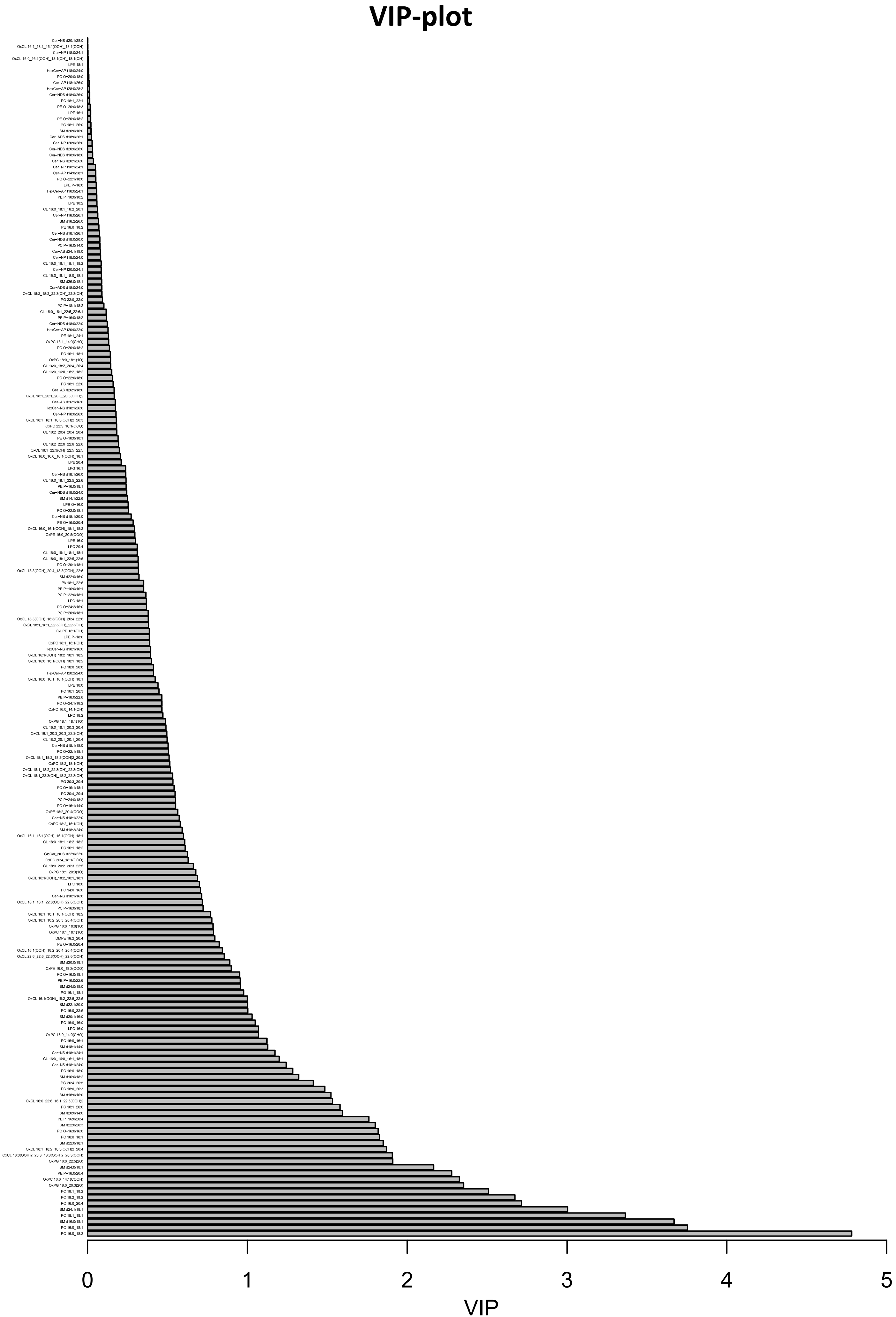

VIP-plot
0
1
2
3
4
5
VIP

Cervicitis vs LSIL

$R^2X = 0.77$
$R^2Y = 0.09$
$Q^2Y = -0.04$

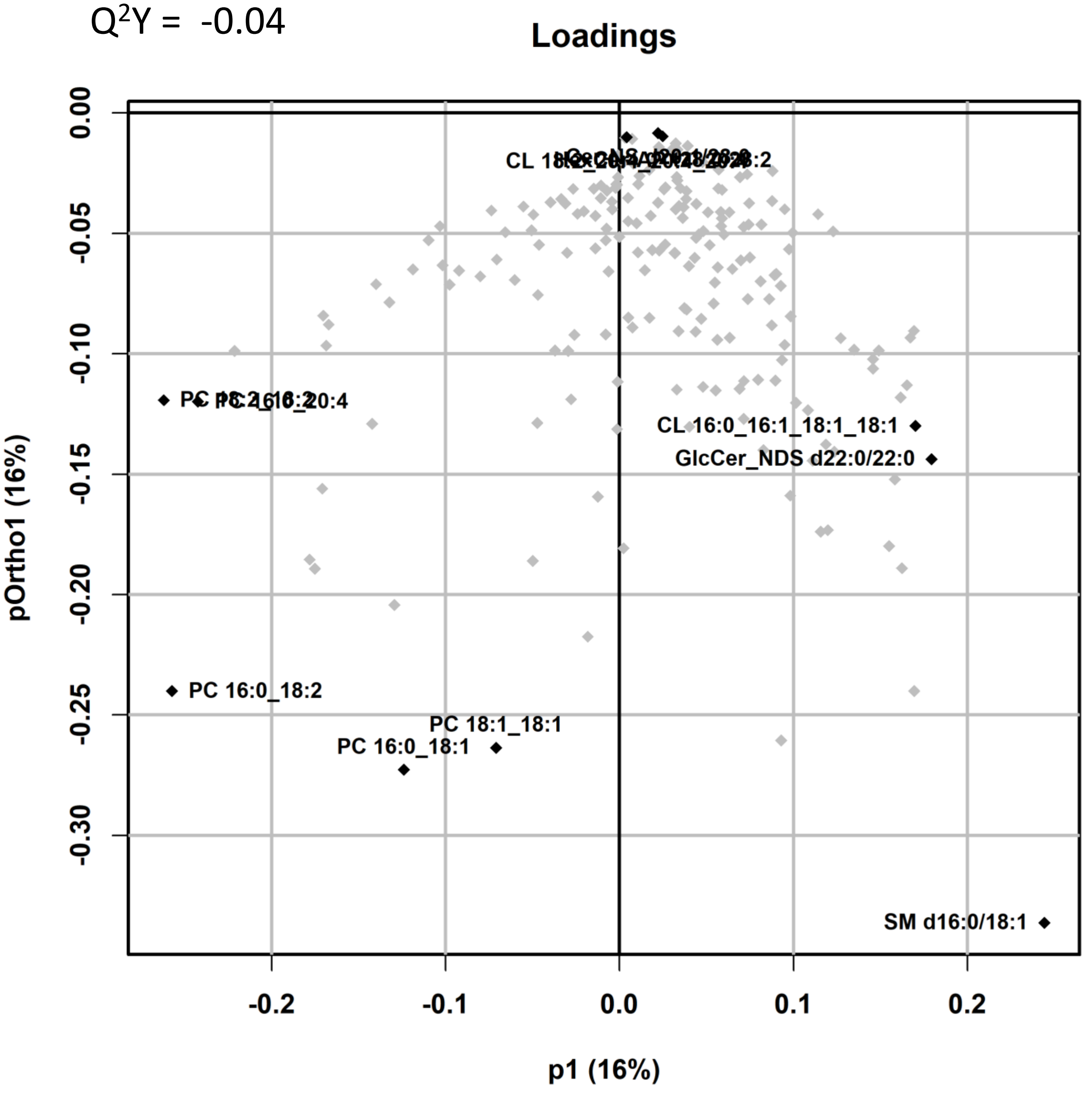

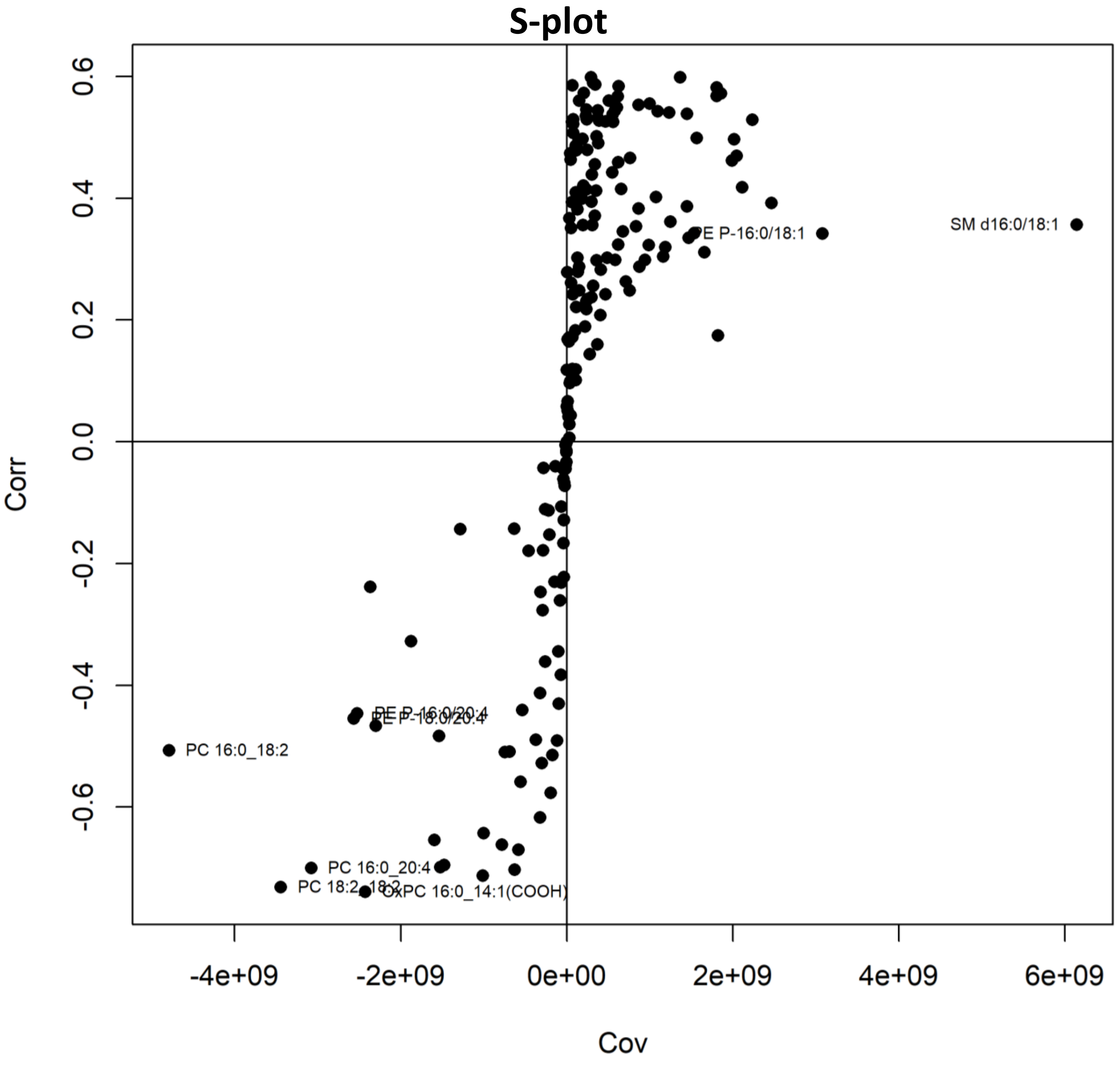
S-plot
Corr
Cov
0.6
0.4
0.2
0.0
-0.2
-0.4
-0.6
-4e+09
-2e+09
0e+00
2e+09
4e+09
6e+09
SM d16:0/18:1
PE P-16:0/18:1
PE P-16:0/20:4
PE P-18:0/20:4
PC 16:0_18:2
PC 16:0_20:4
PC 18:2_18:2
OxPC 16:0_14:1(COOH)

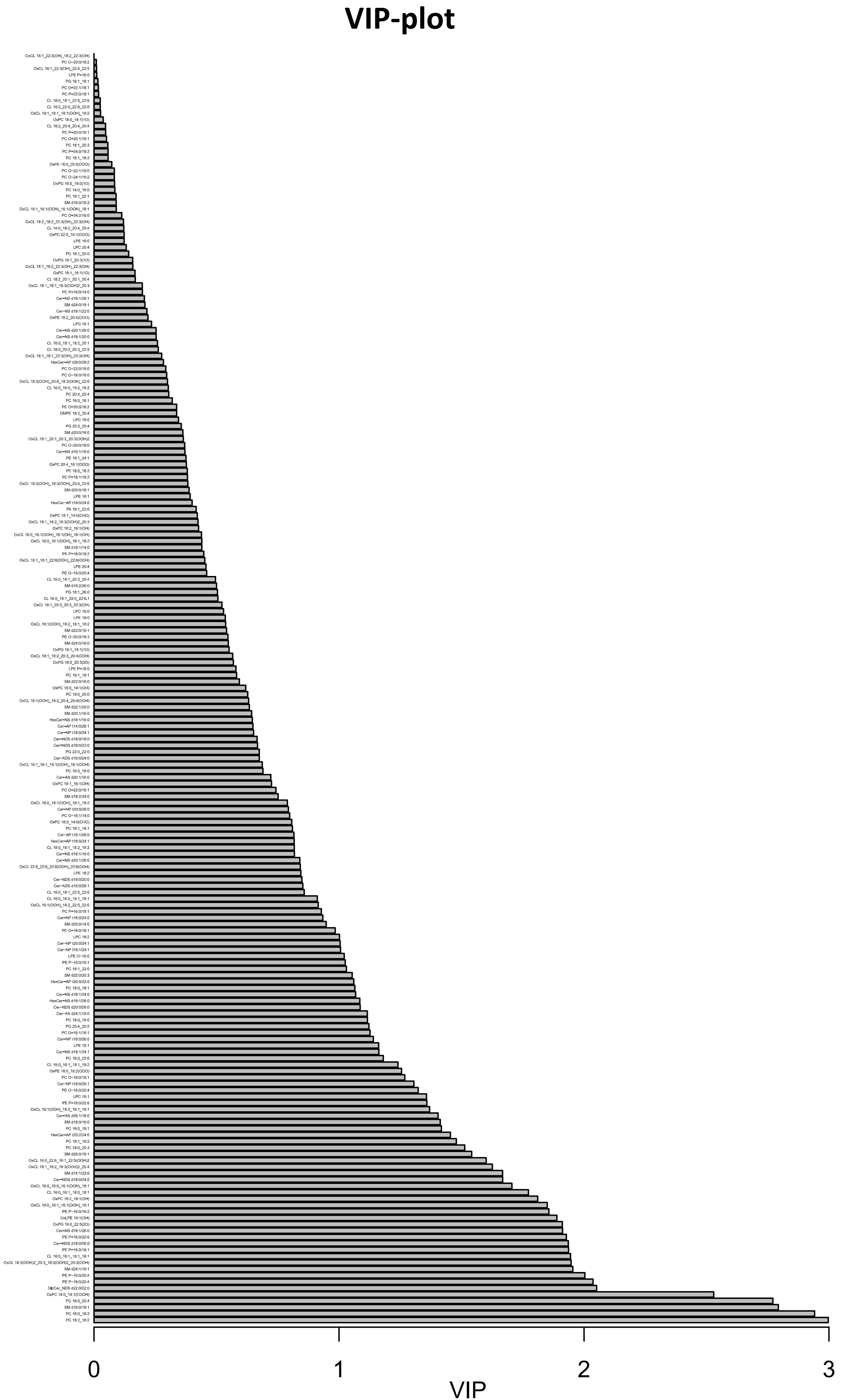

VIP-plot
0
1
2
3
VIP

Cervicitis vs HSIL

$R^2X = 0.76$
$R^2Y = 0.15$
$Q^2Y = -0.08$

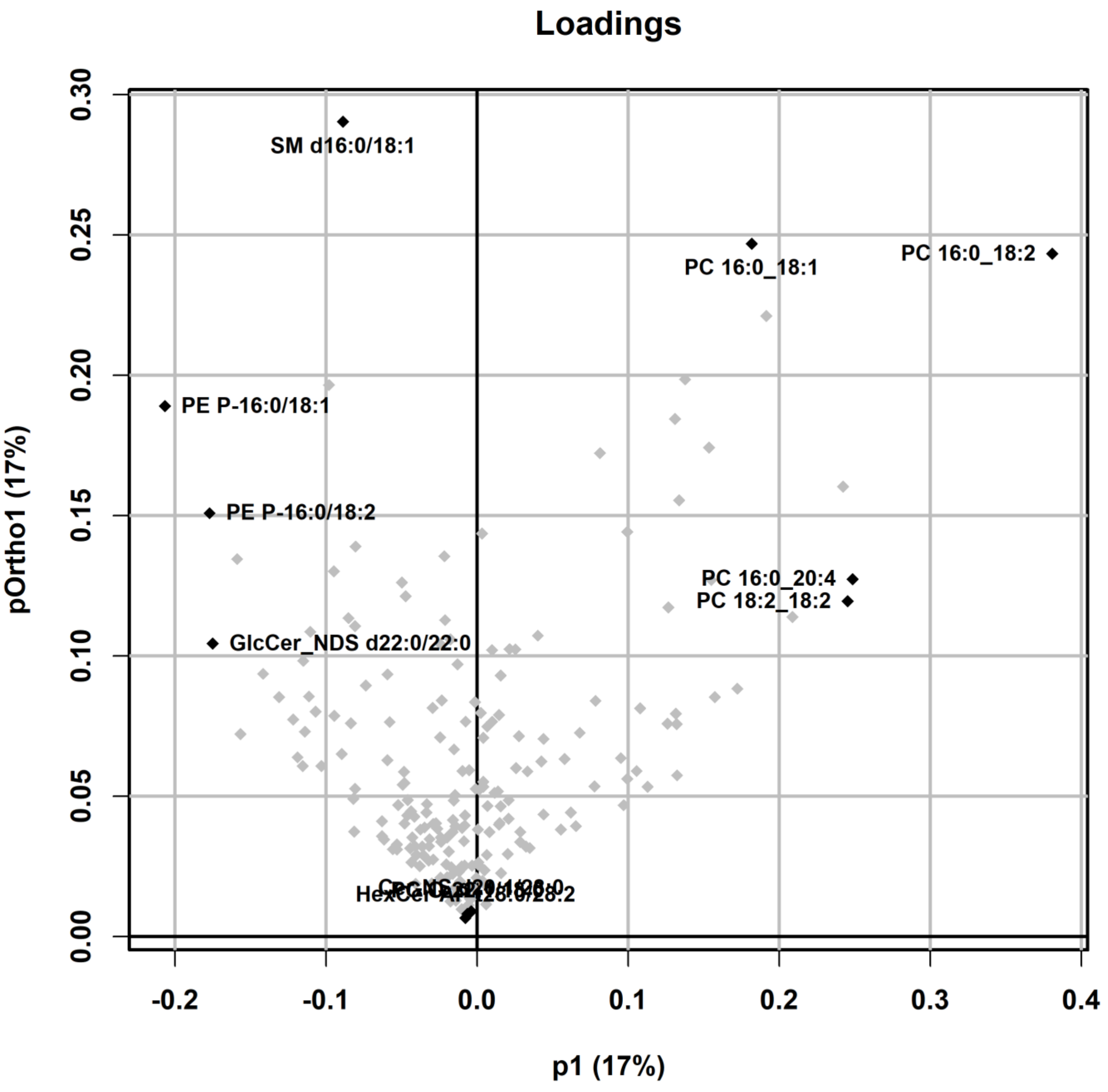

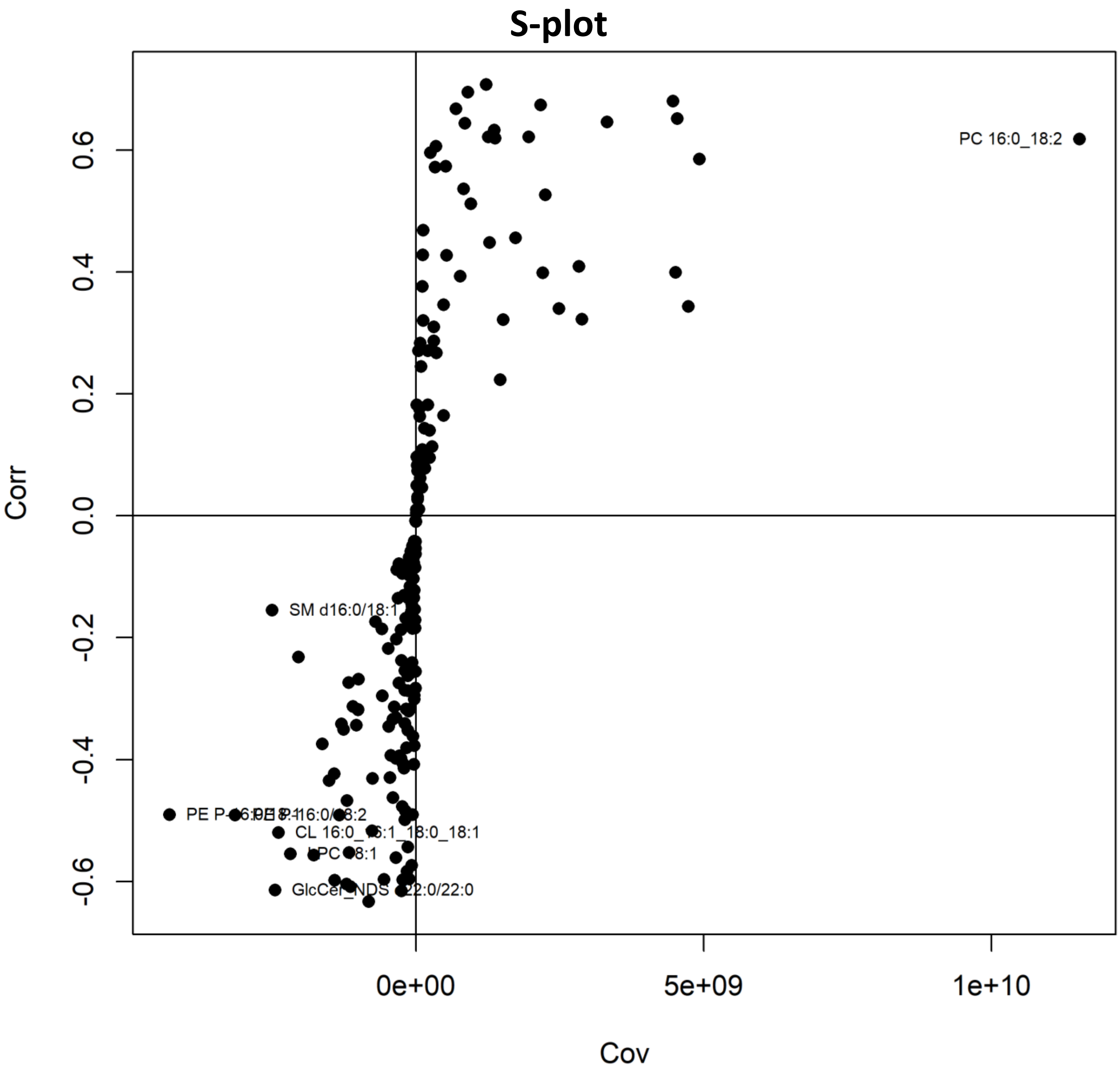

S-plot
PC 16:0_18:2
SM d16:0/18:1
GlcCer_NDS 22:0/22:0
0.6
0.4
0.2
0.0
-0.2
-0.4
-0.6
Corr
0e+00
5e+09
1e+10
Cov

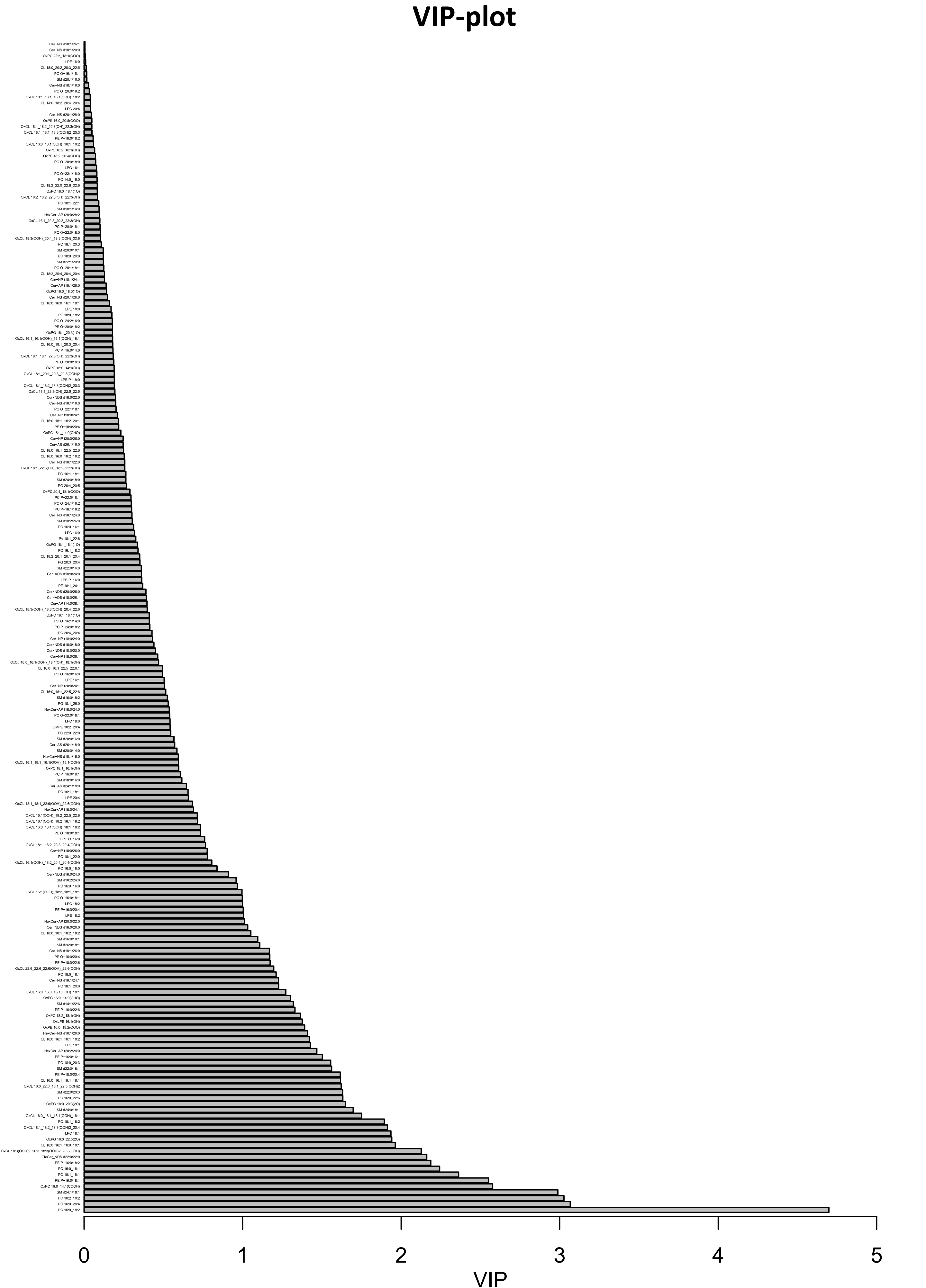
VIP-plot
0
1
2
3
4
5
VIP

Cervicitis vs cancer

$R^2X = 0.77$
$R^2Y = 0.38$
$Q^2Y = 0.27$

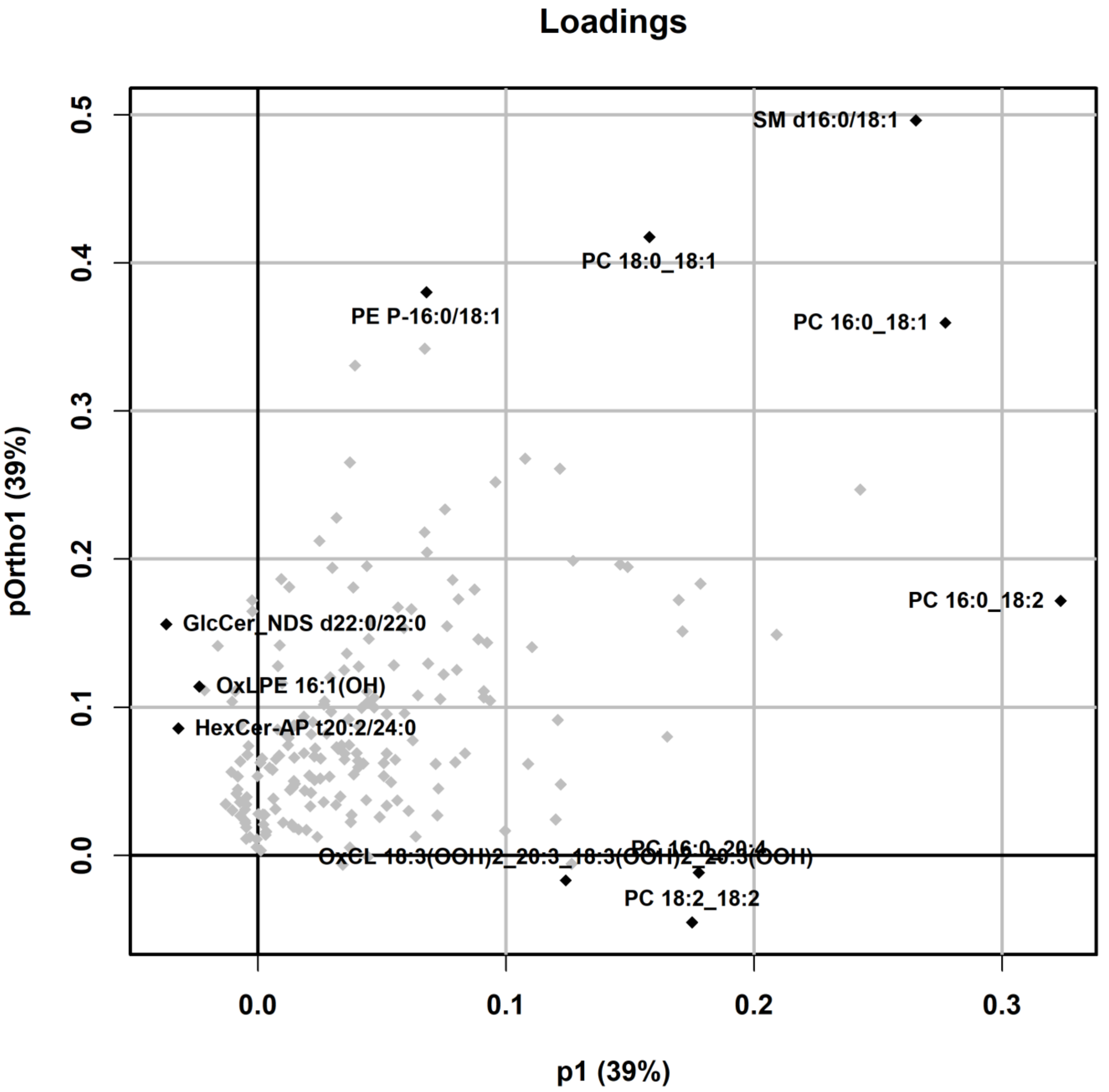

S-plot

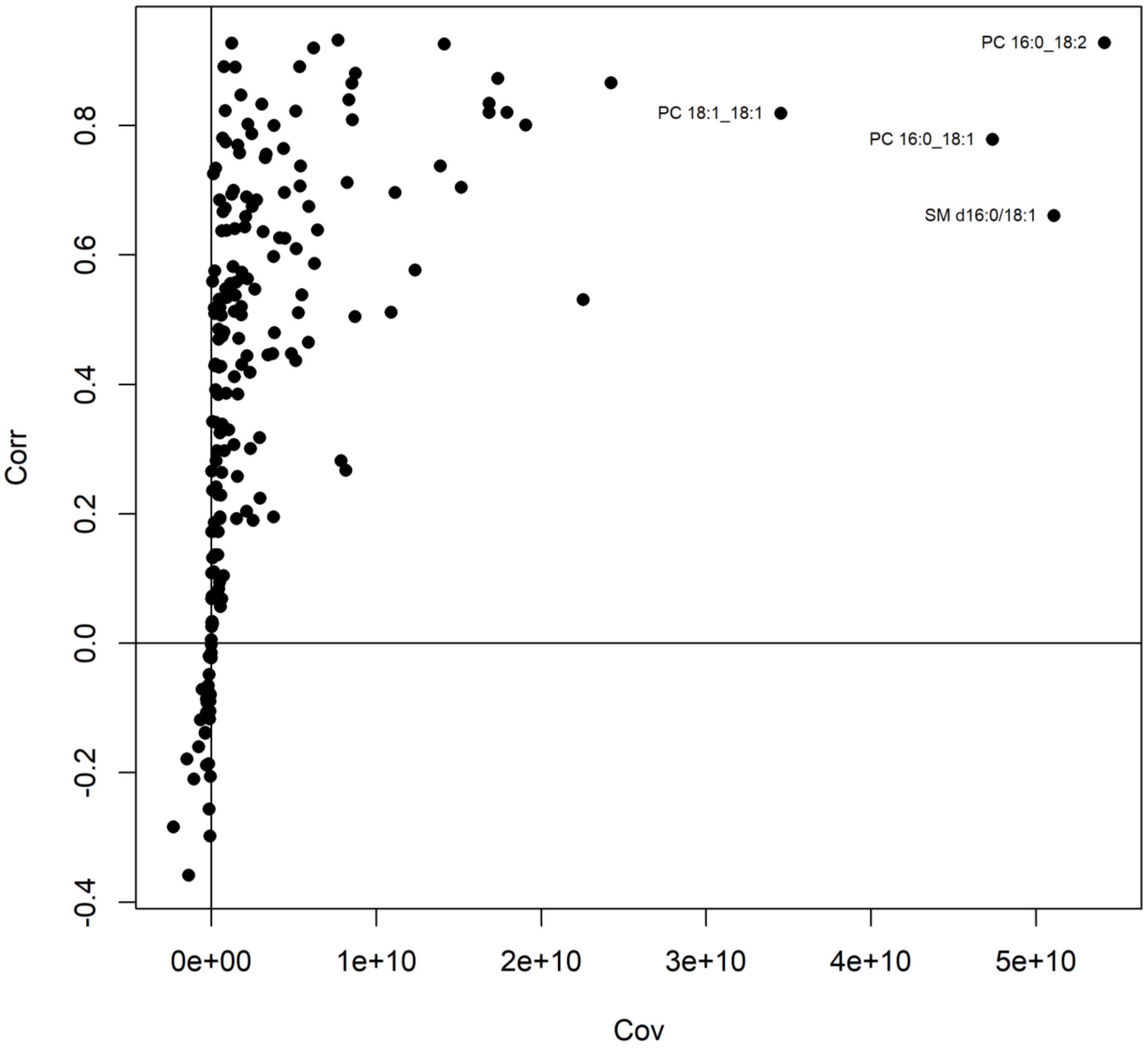

PC 16:0_18:2
PC 18:1_18:1
PC 16:0_18:1
SM d16:0/18:1
Corr
Cov
0.8
0.6
0.4
0.2
0.0
-0.2
-0.4
0e+00
1e+10
2e+10
3e+10
4e+10
5e+10

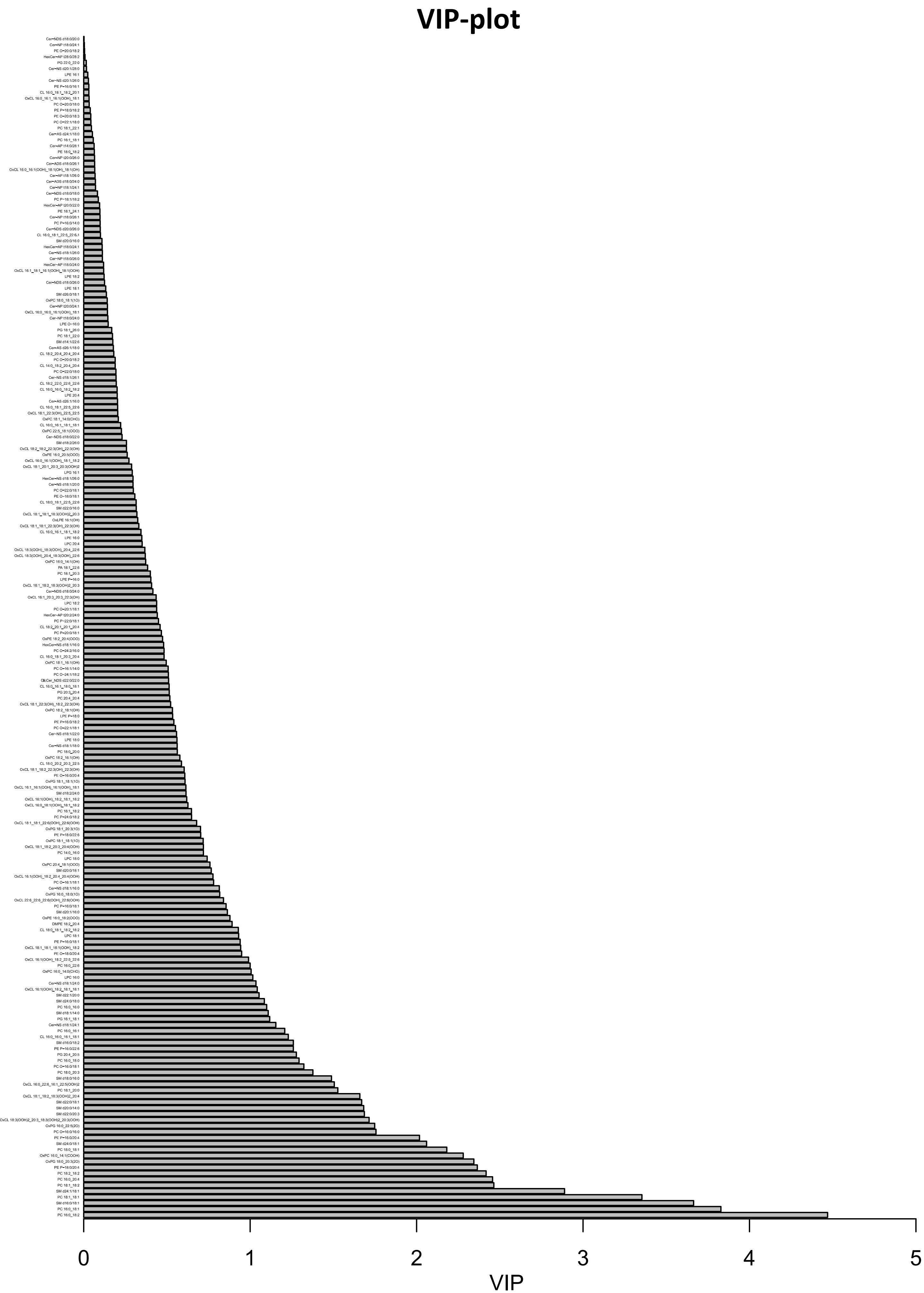
VIP-plot
0
1
2
3
4
5
VIP

LSIL vs HSIL

$R^2X = 0.76$
$R^2Y = 0.18$
$Q^2Y = 0.08$

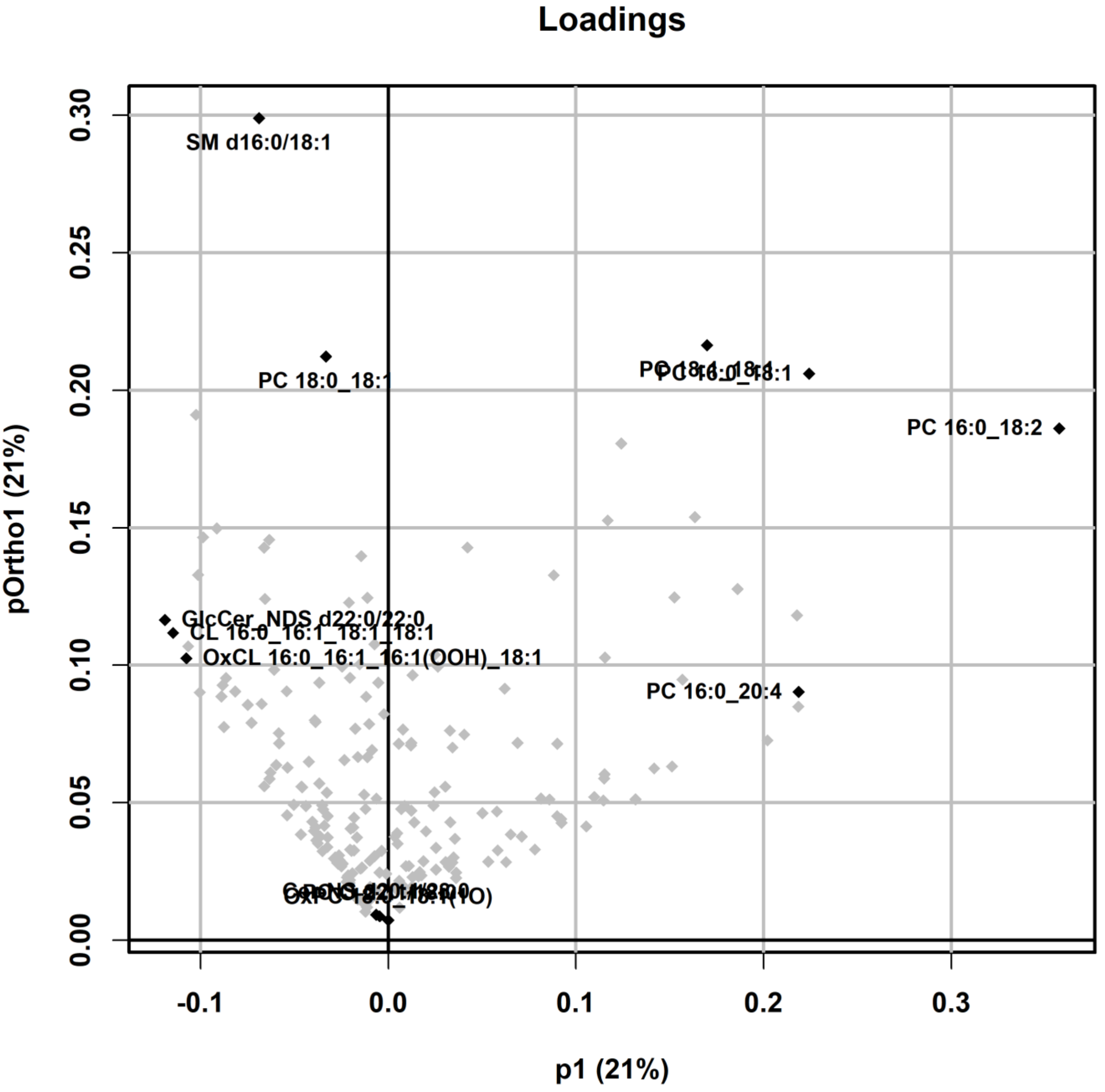

S-plot

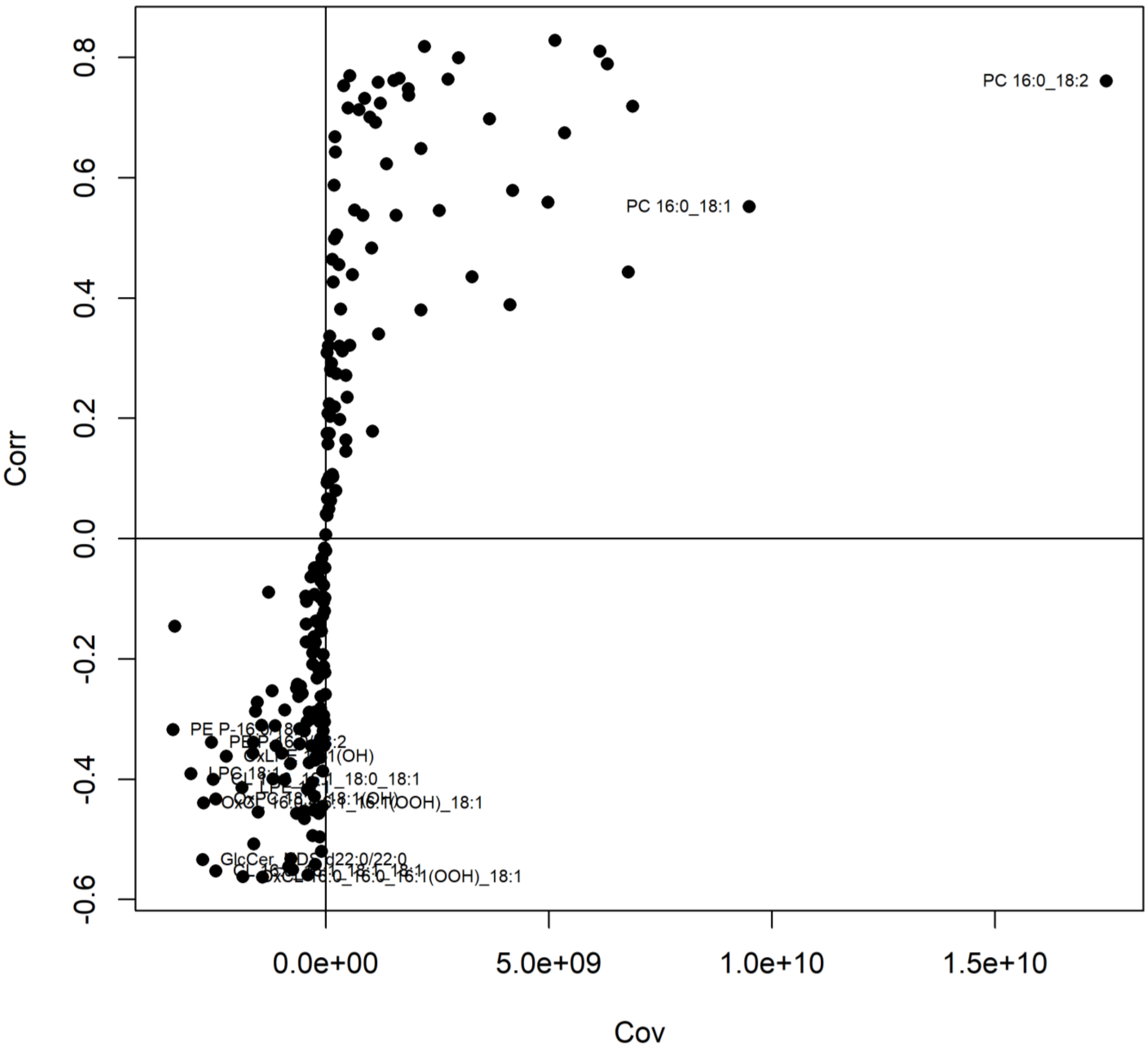

PC 16:0_18:2
PC 16:0_18:1
0.8
0.6
0.4
0.2
0.0
-0.2
-0.4
-0.6
Corr
0.0e+00
5.0e+09
1.0e+10
1.5e+10
Cov

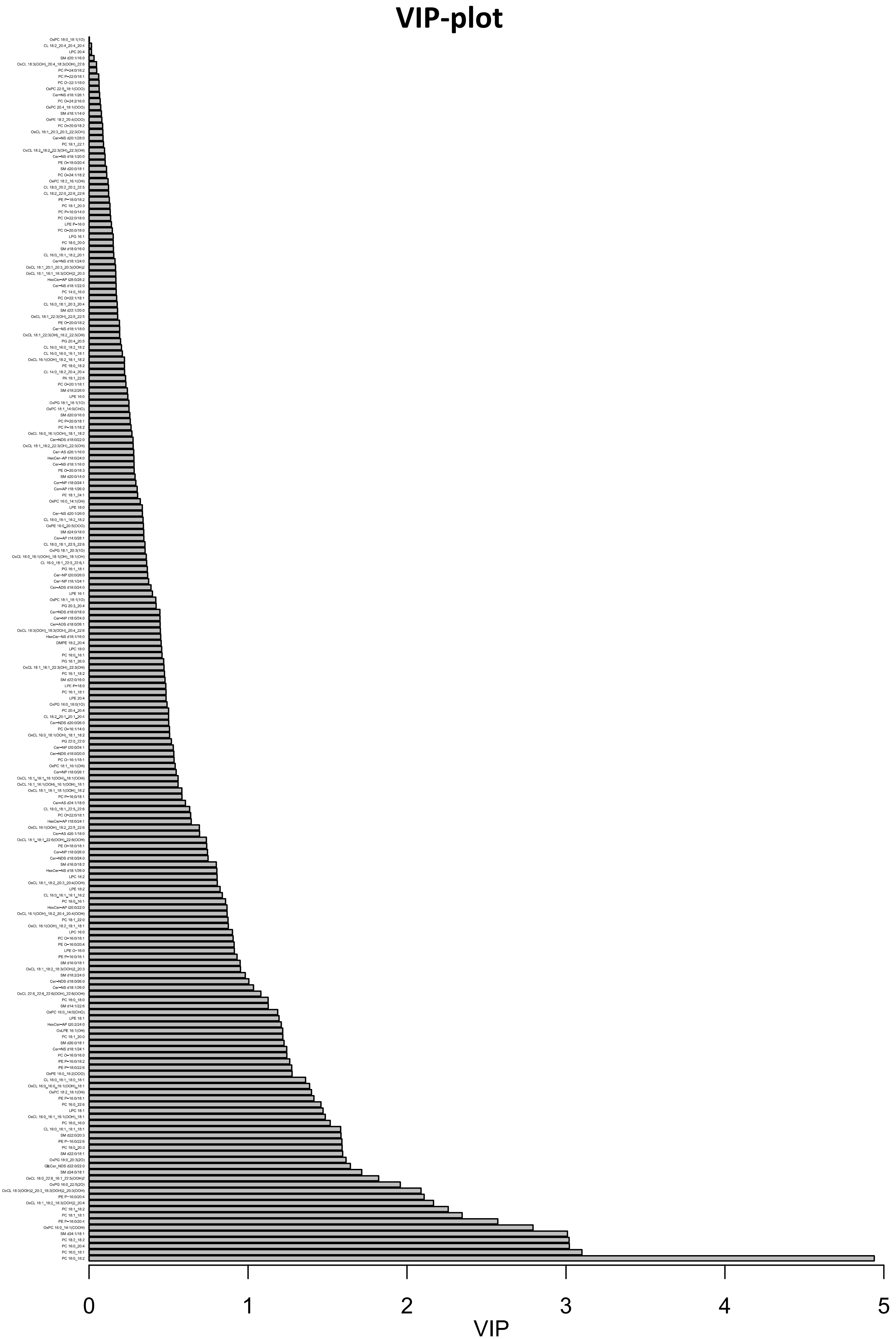
VIP-plot
0
1
2
3
4
5
VIP

LSIL vs cancer

$R^2X = 0.77$
$R^2Y = 0.42$
$Q^2Y = 0.34$

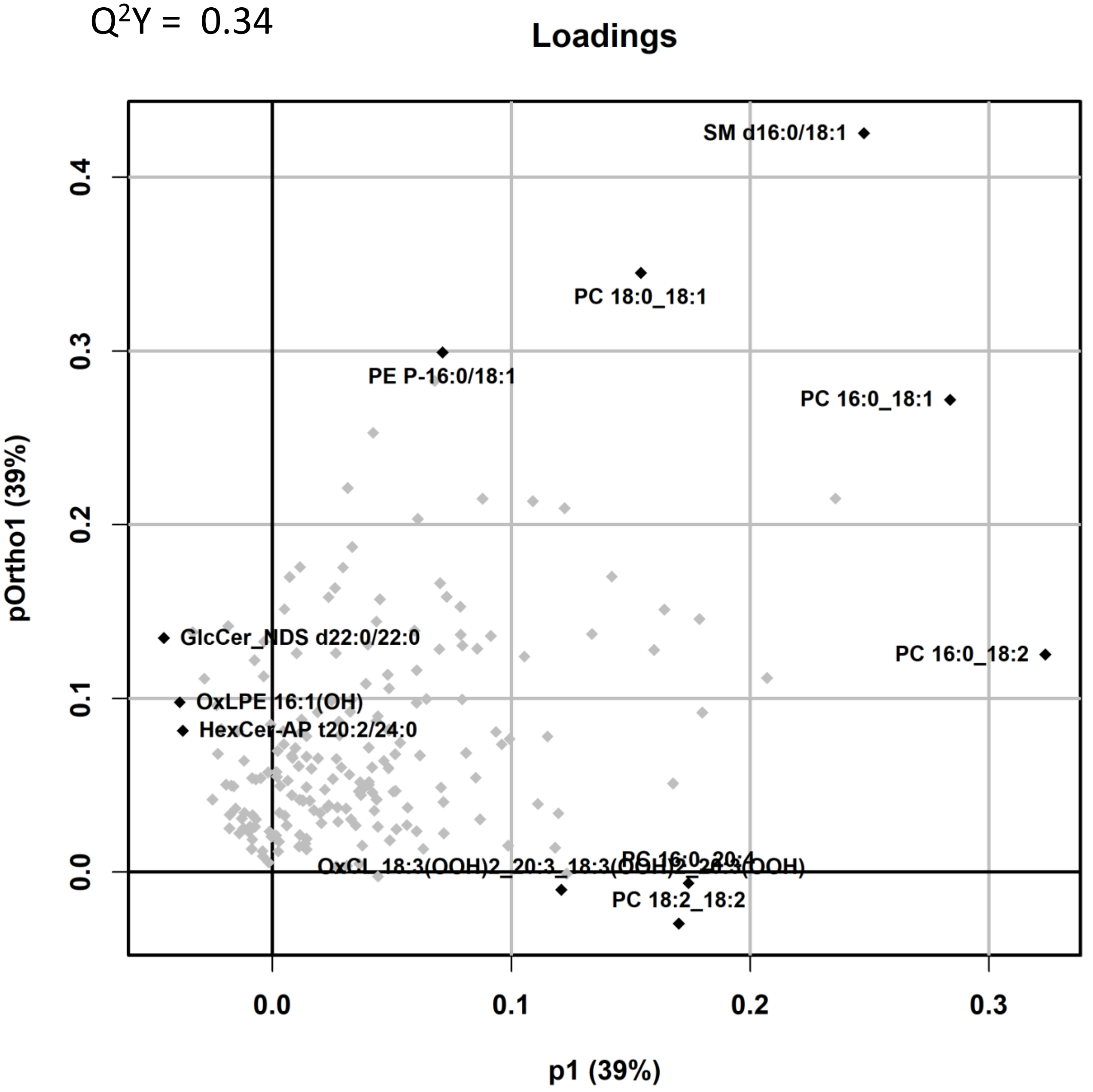

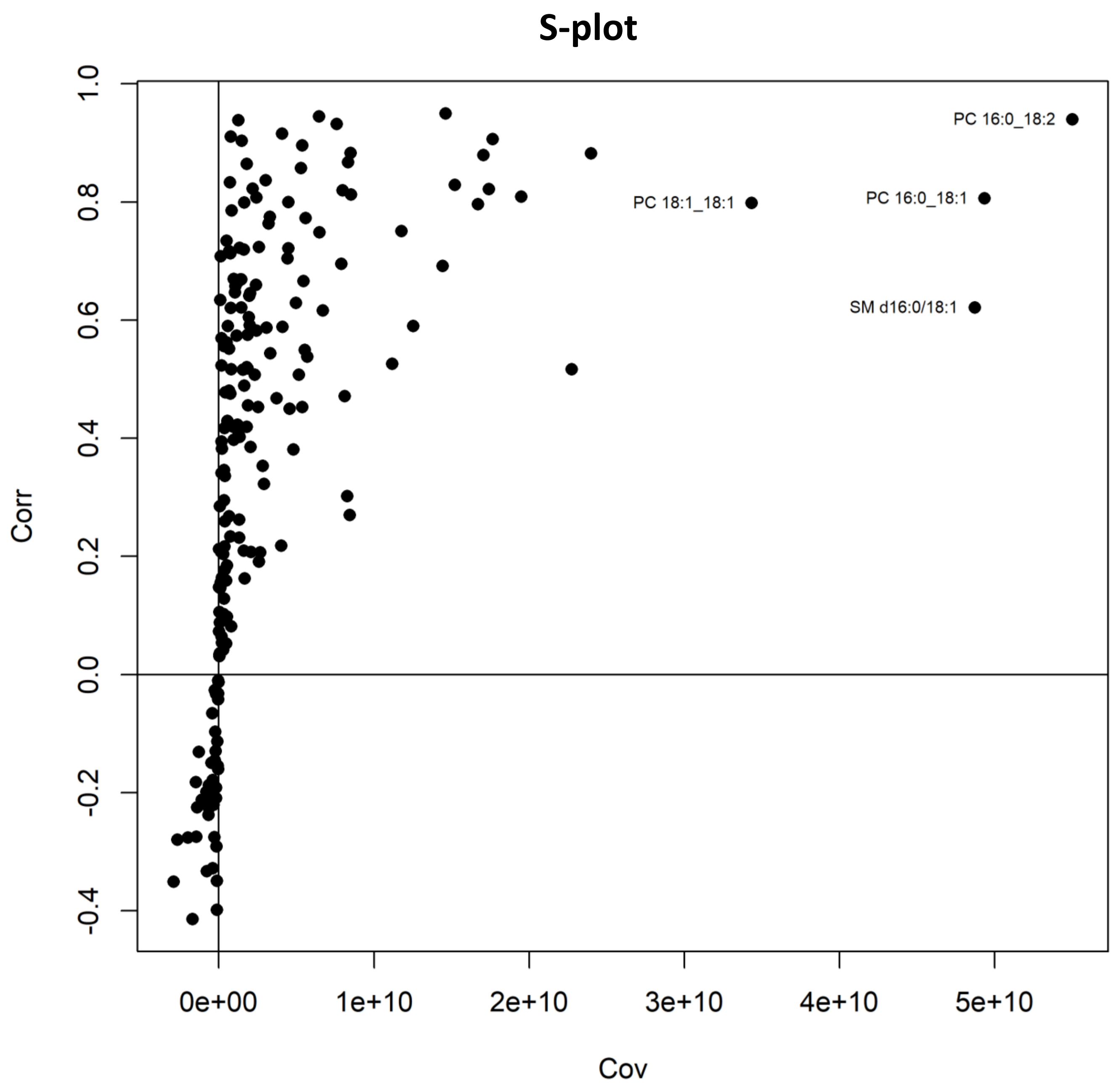
S-plot
Corr
Cov
1.0
0.8
0.6
0.4
0.2
0.0
-0.2
-0.4
0e+00
1e+10
2e+10
3e+10
4e+10
5e+10
PC 16:0_18:2
PC 18:1_18:1
PC 16:0_18:1
SM d16:0/18:1

VIP-plot

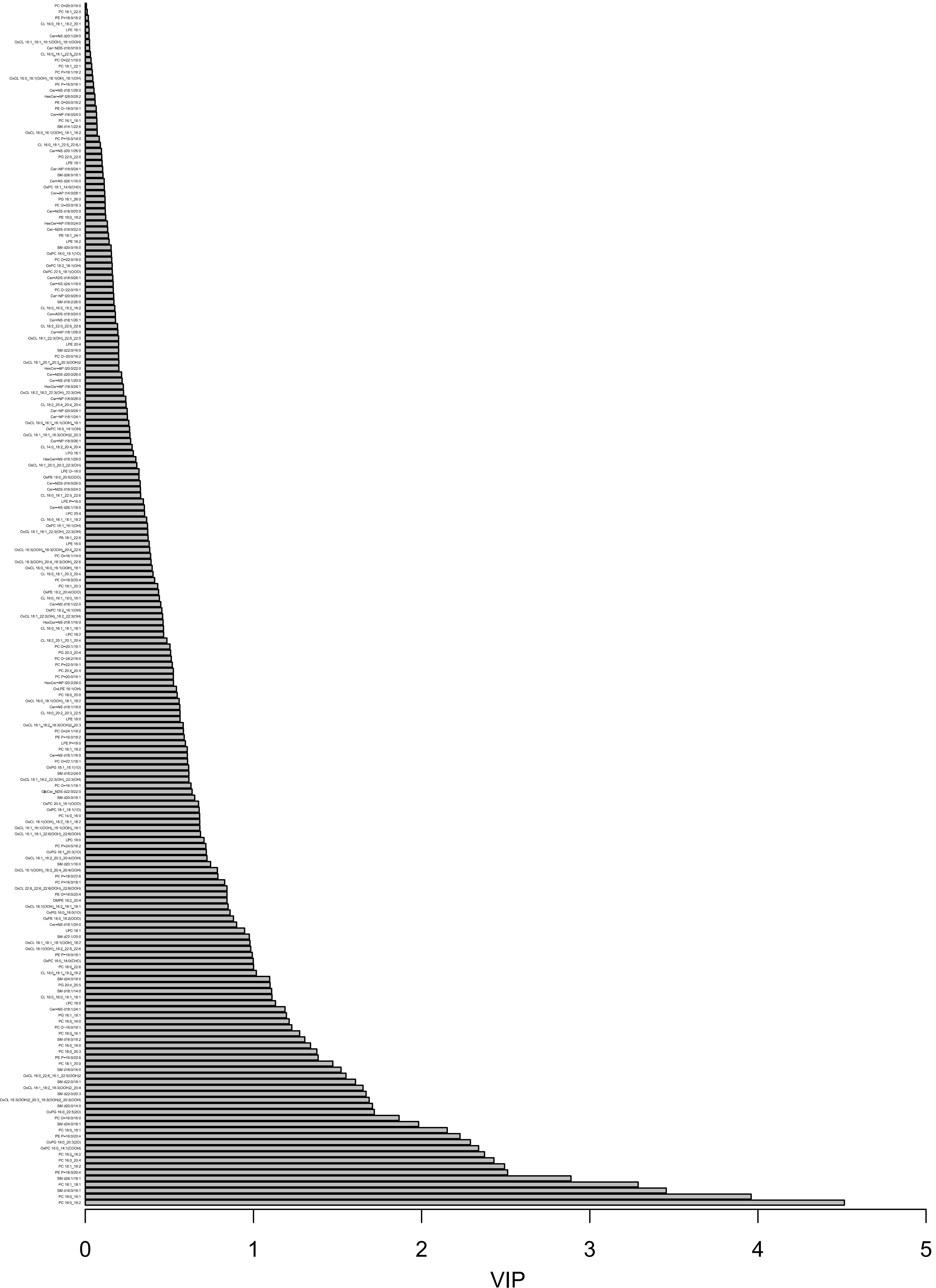

HSIL vs cancer

$R^2X = 0.68$
$R^2Y = 0.48$
$Q^2Y = - 0.05$

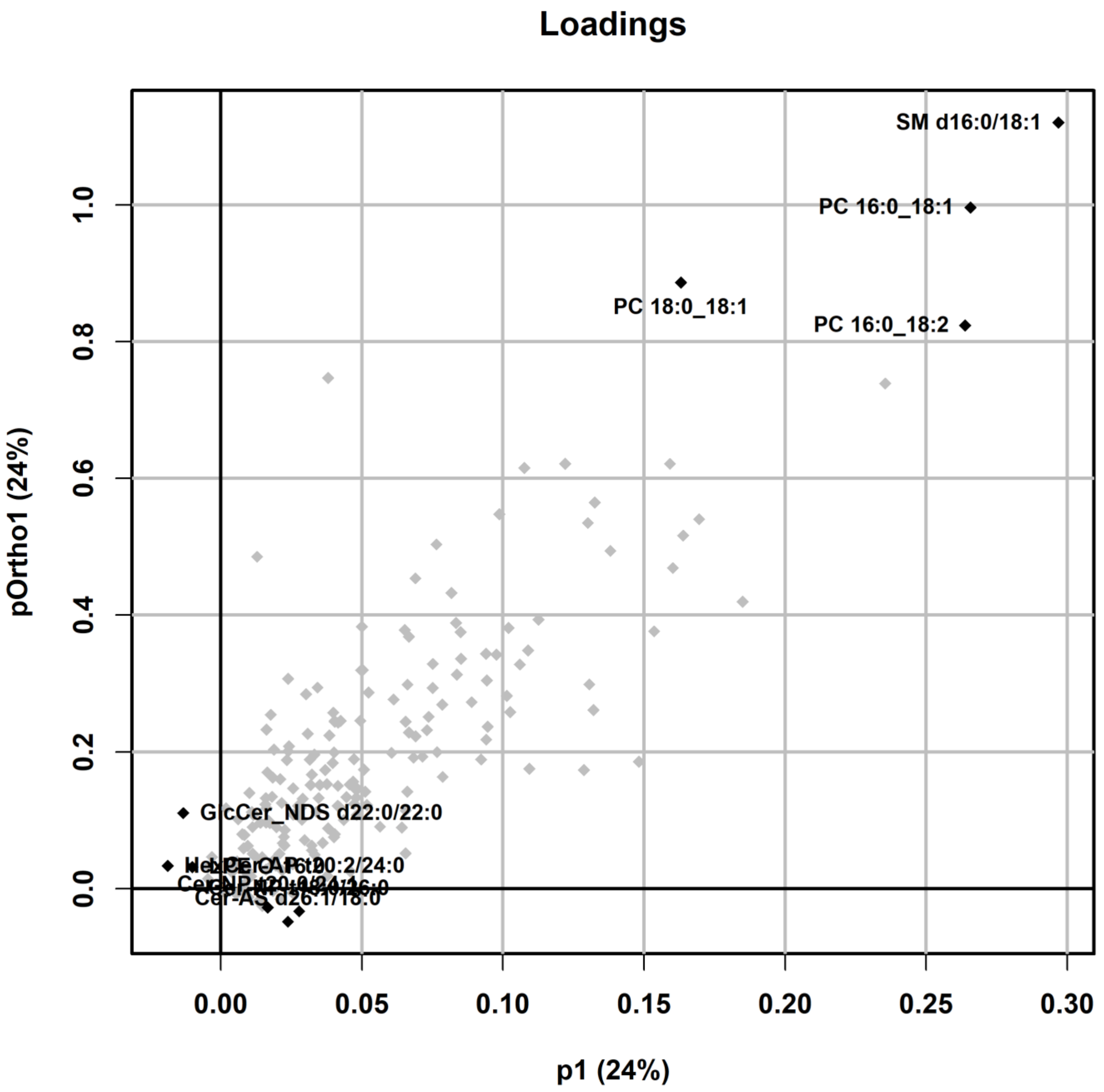

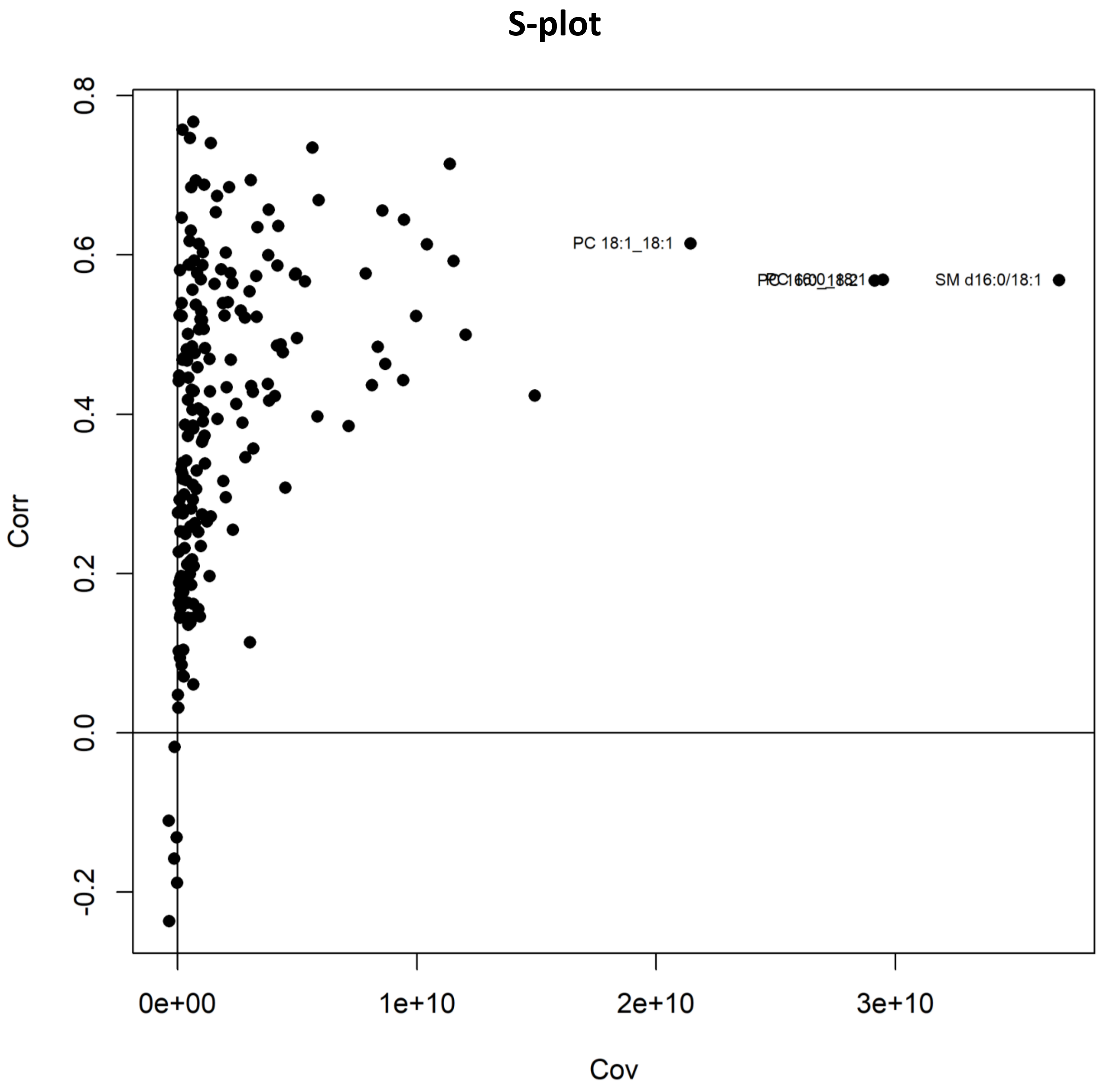
S-plot
Corr
0.8
0.6
0.4
0.2
0.0
-0.2
Cov
0e+00
1e+10
2e+10
3e+10
PC 18:1_18:1
SM d16:0/18:1

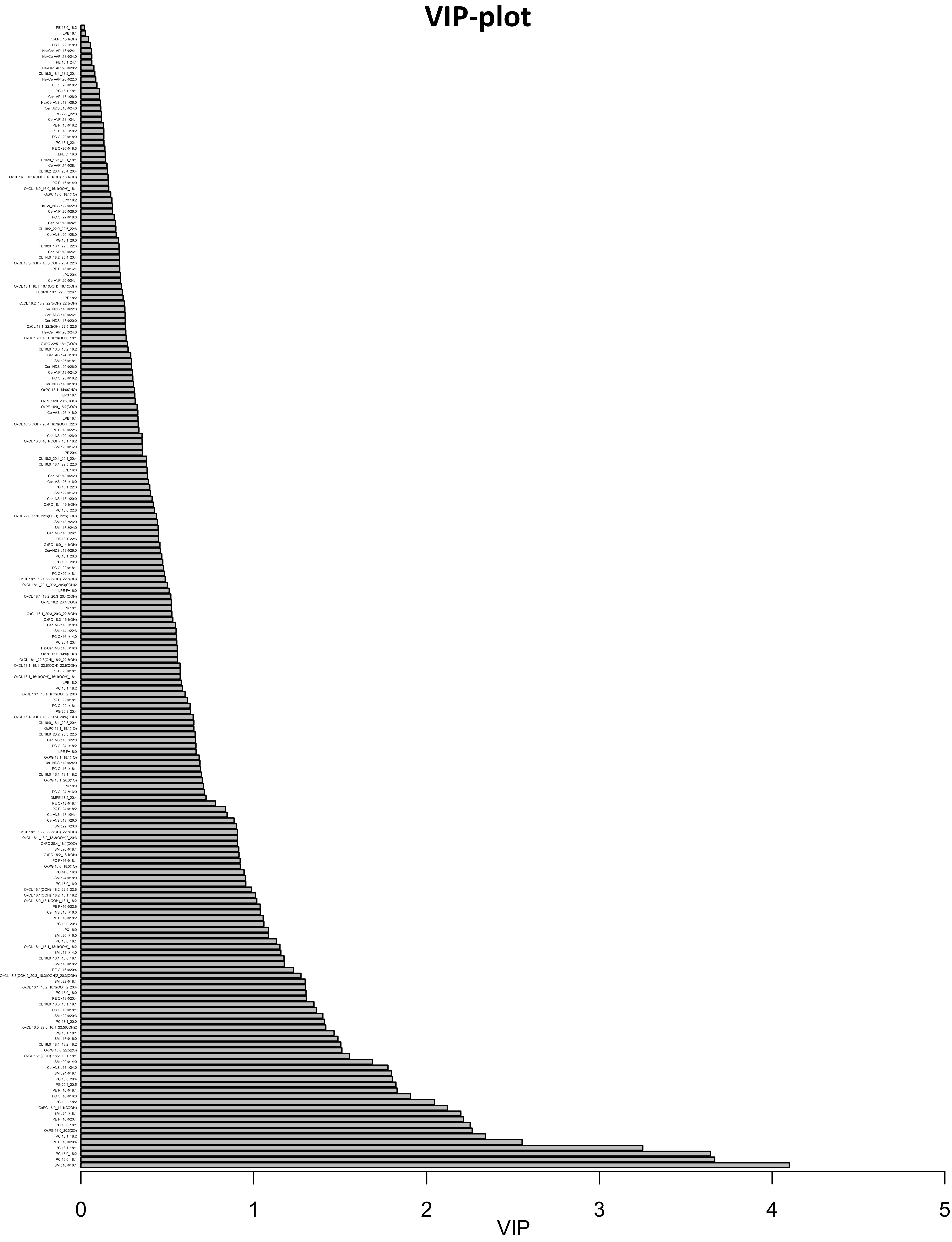
VIP-plot
0
1
2
3
4
5
VIP
