# Supplementary figures and images for: Non-Invasive Differential Diagnosis of Cervical Neoplastic Lesions by the Lipid Profile Analysis of Cervical Scrapings
Source: Metabolites. 2022 Sep 19;12(9):883. doi: 10.3390/metabo12090883 (PMC9506087; doi:10.3390/metabo12090883)

# Positive ion mode lipid markers MS/MS

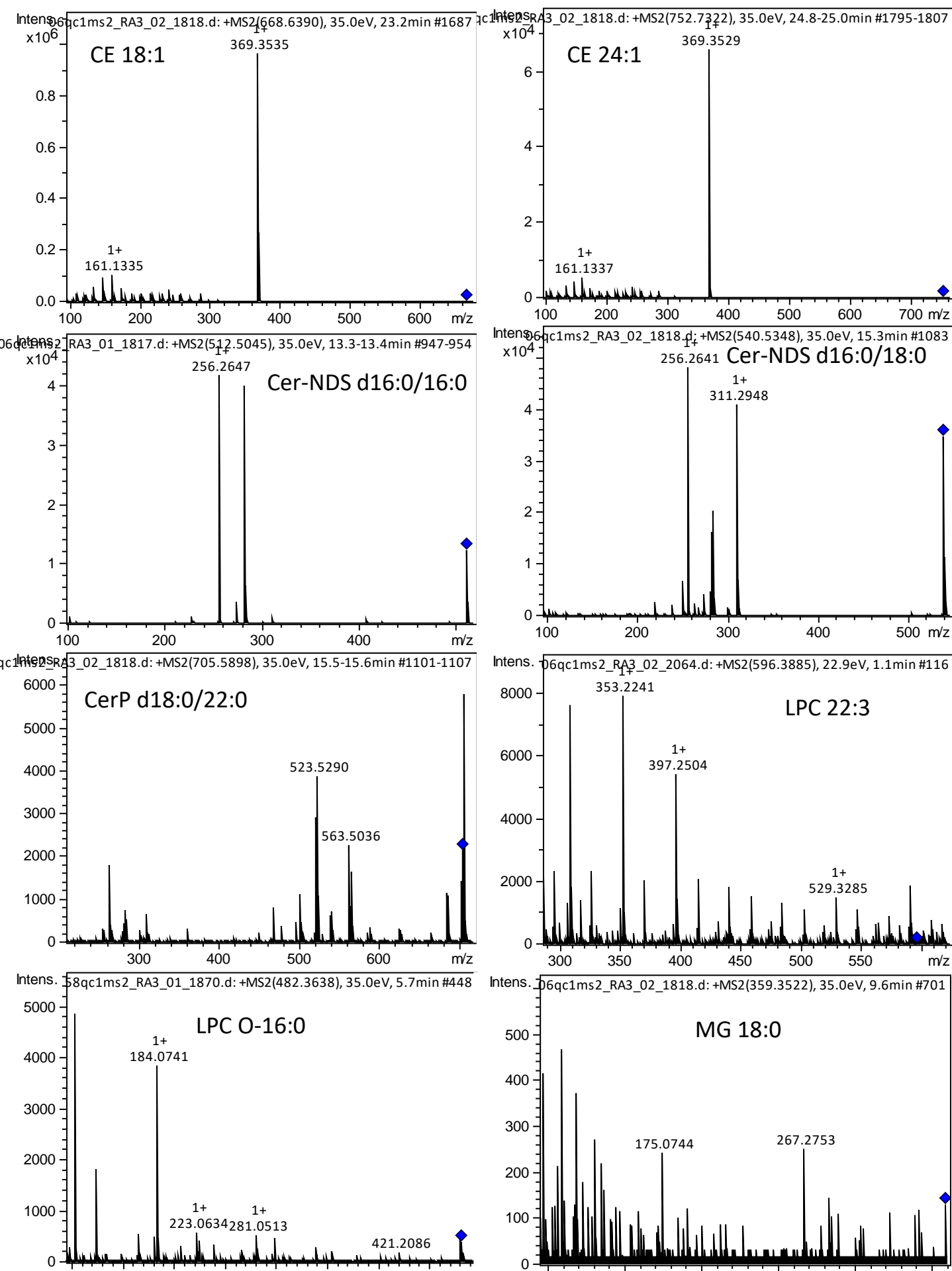

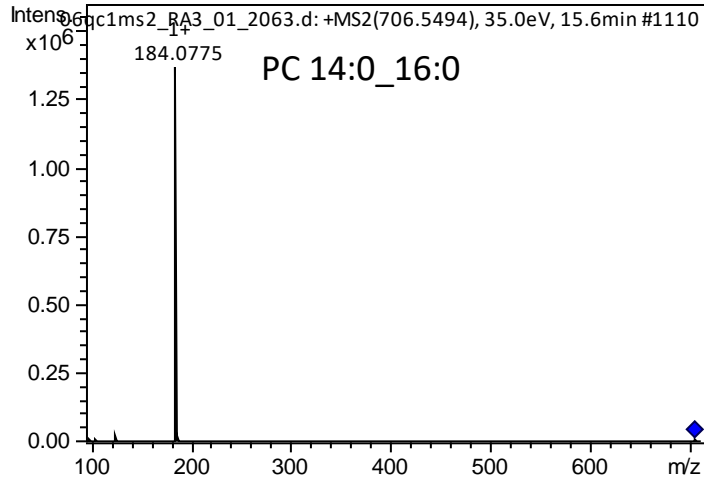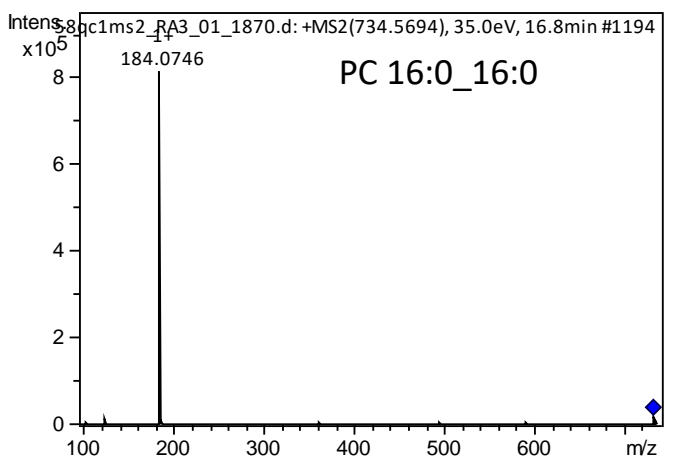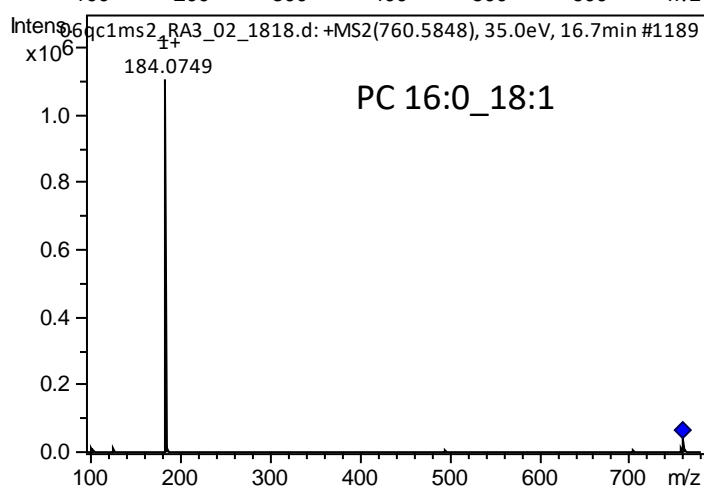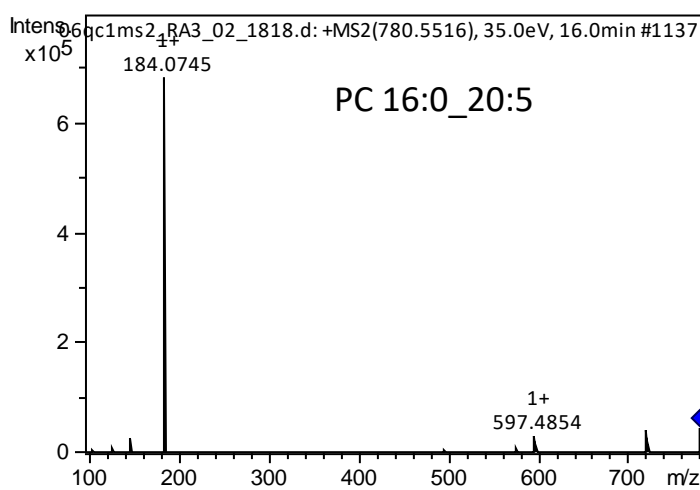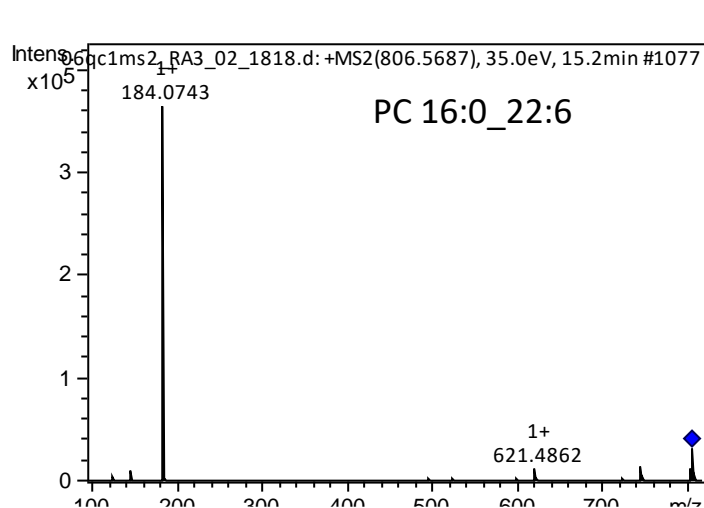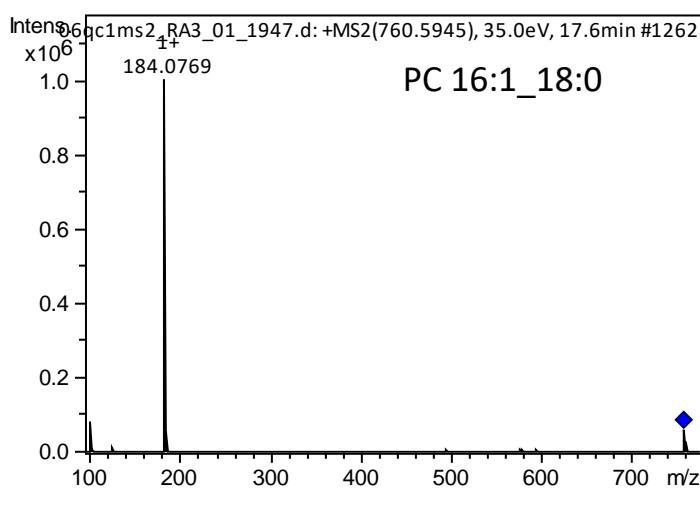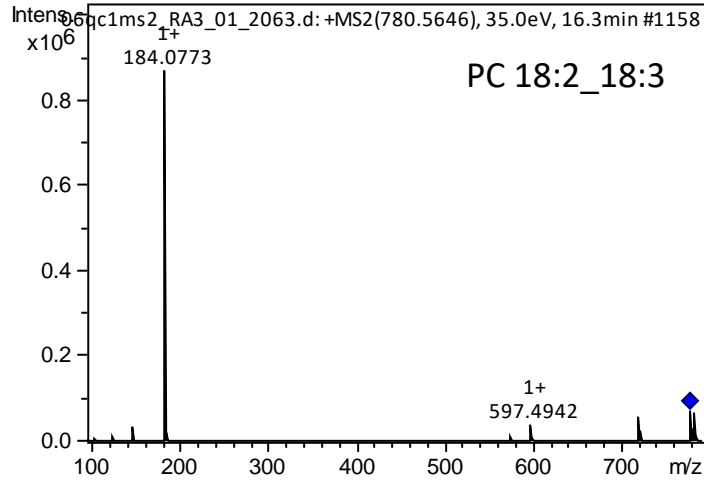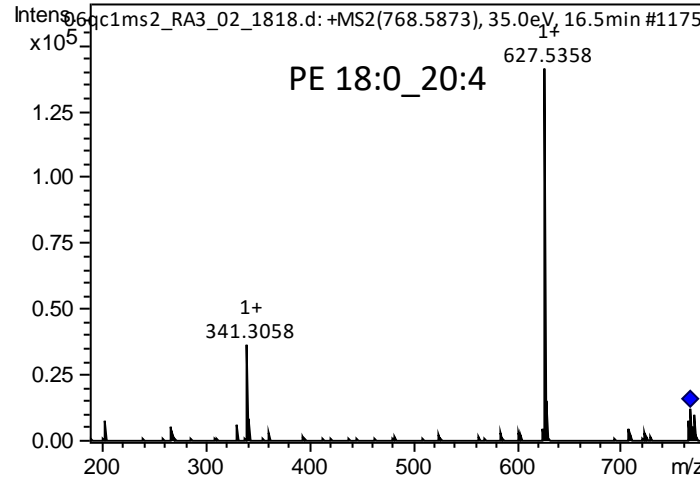

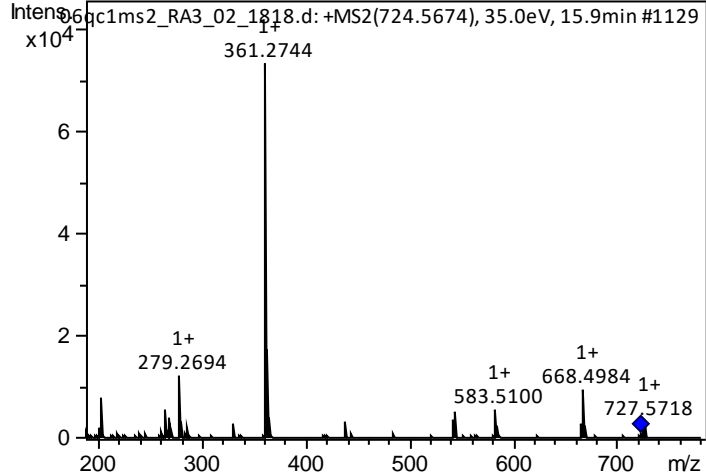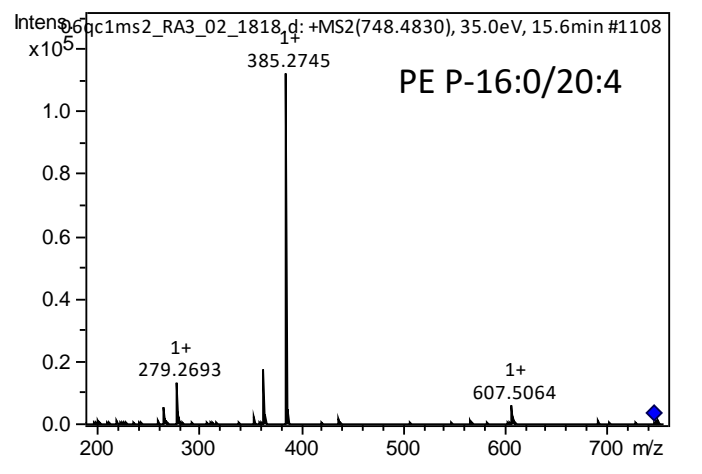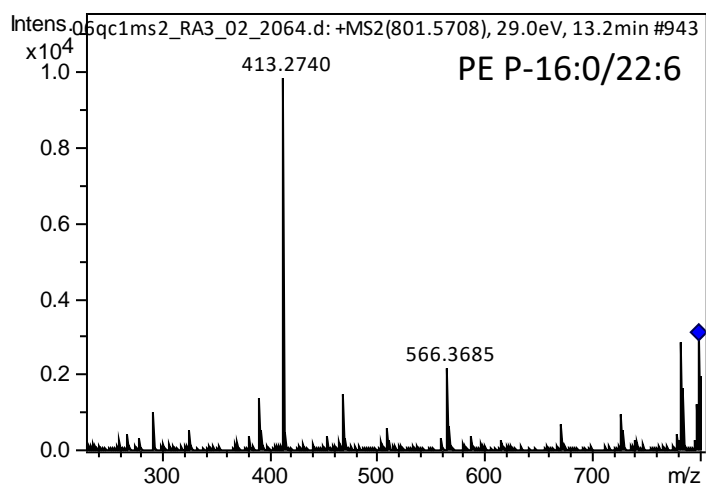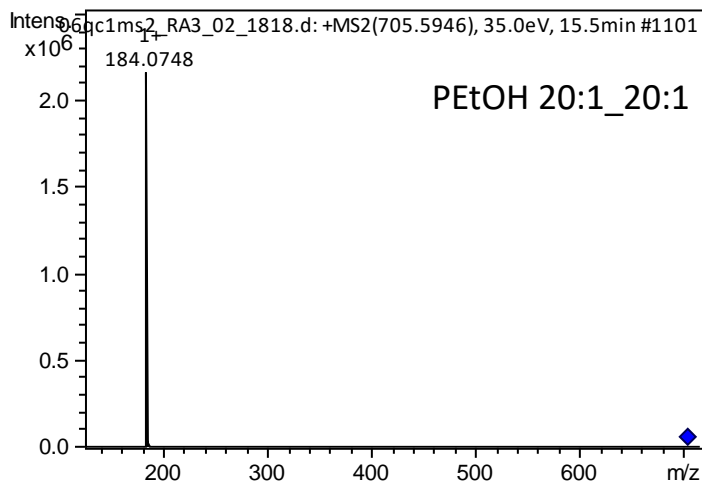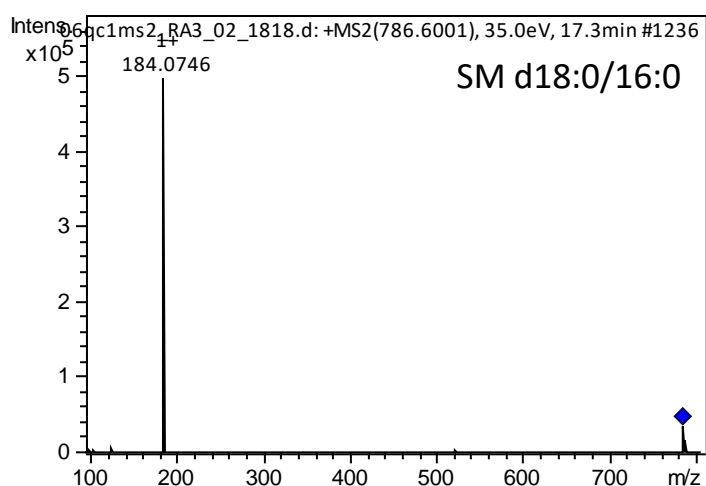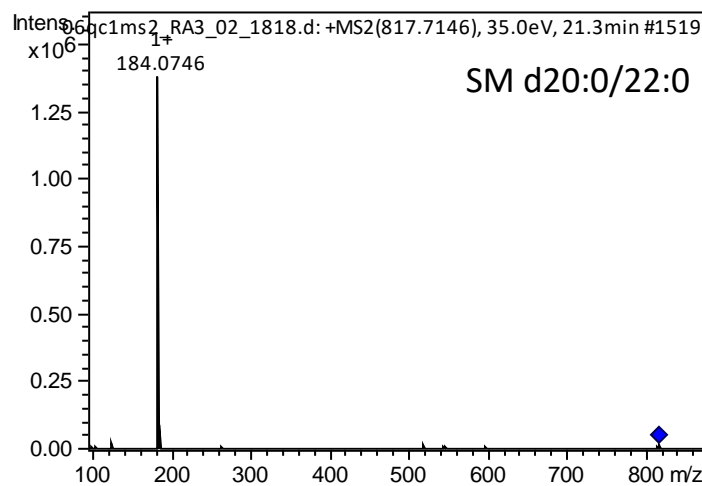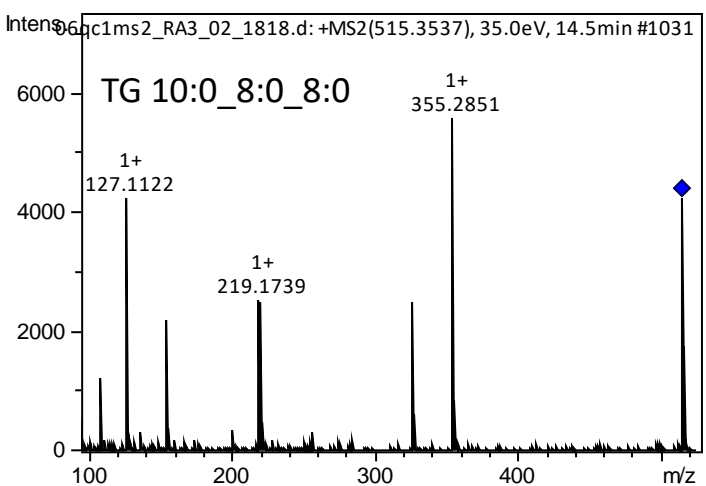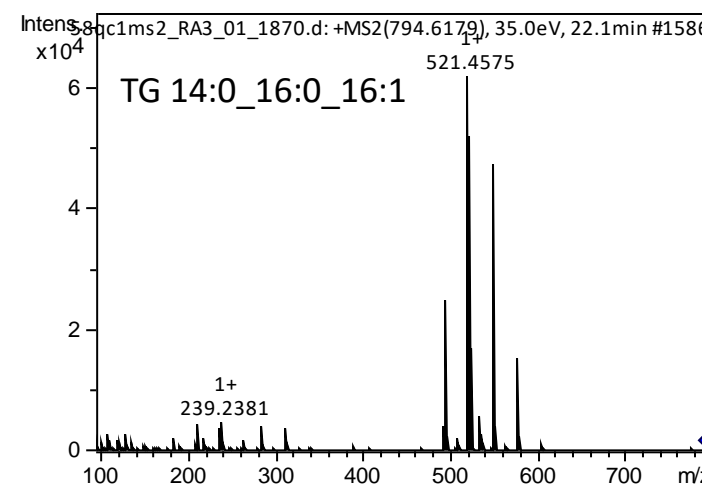

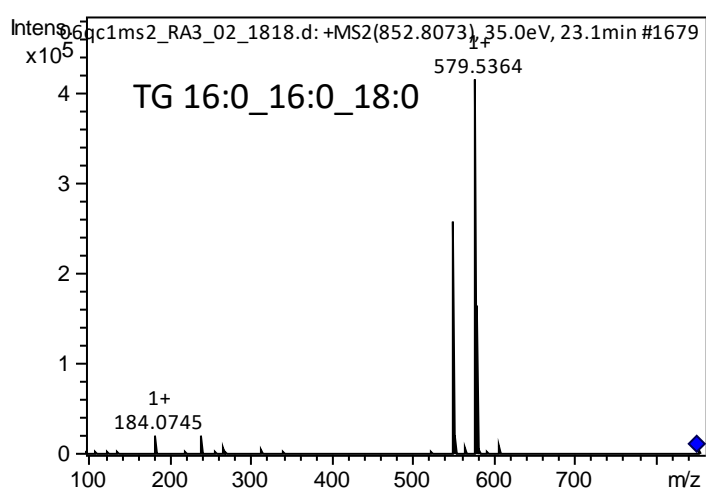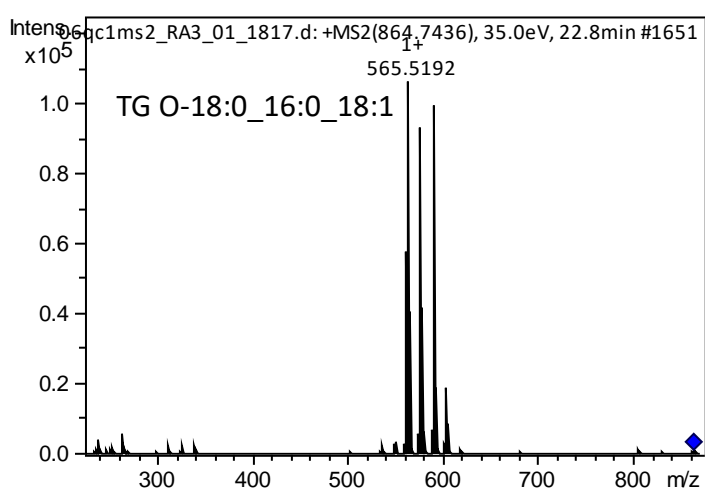

# Negative ion mode lipid markers MS/MS

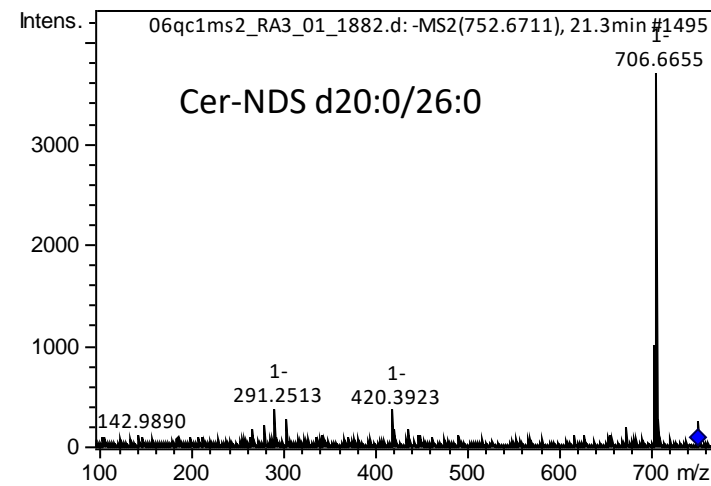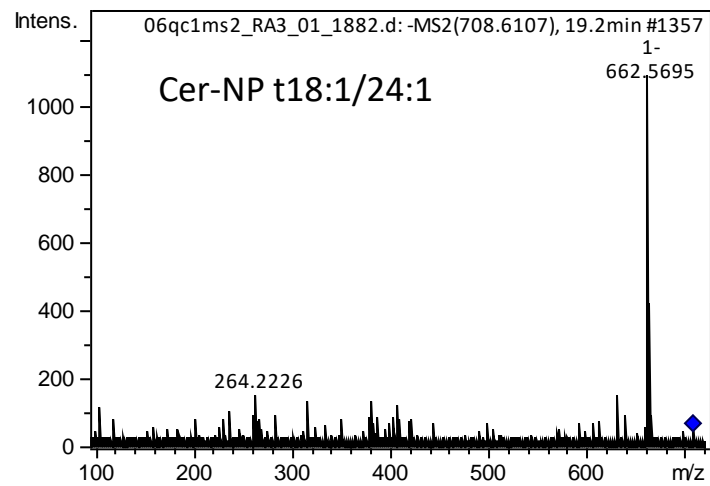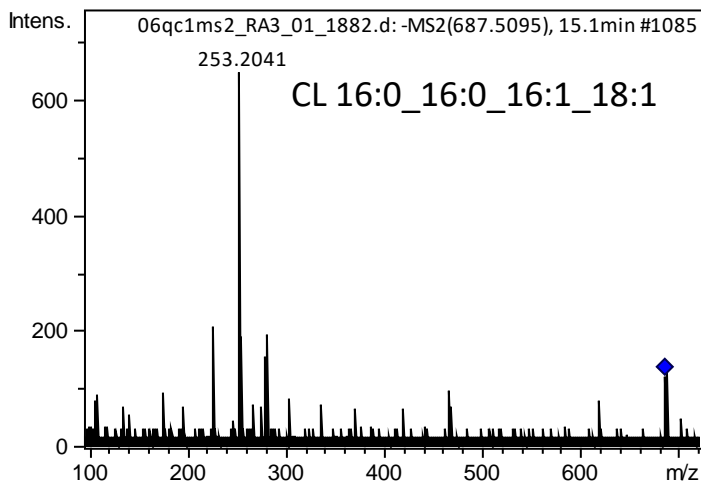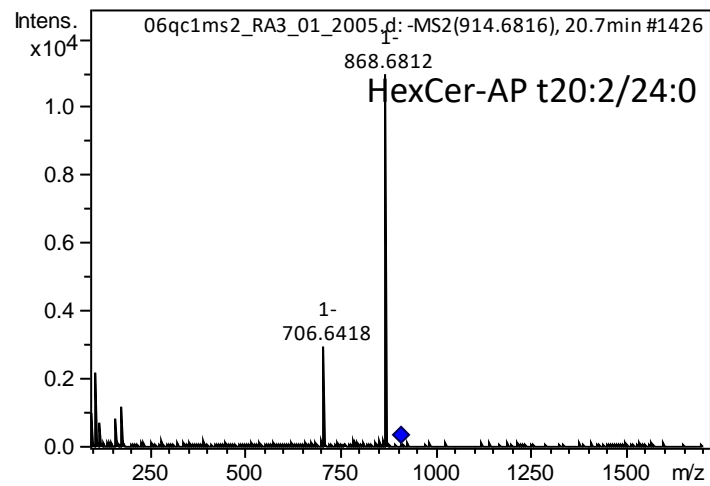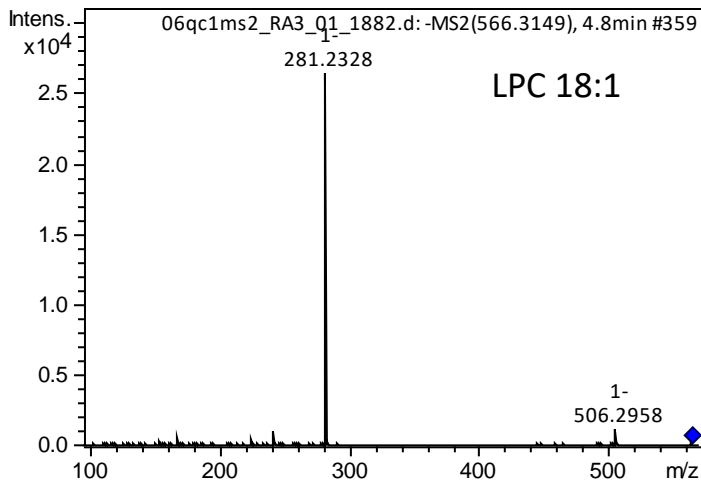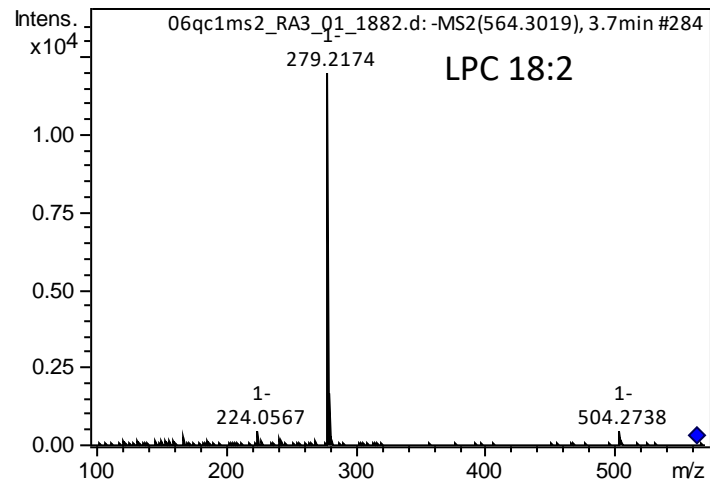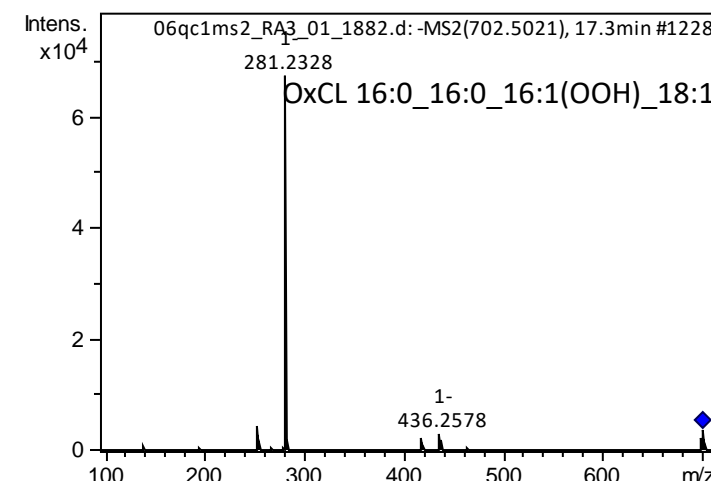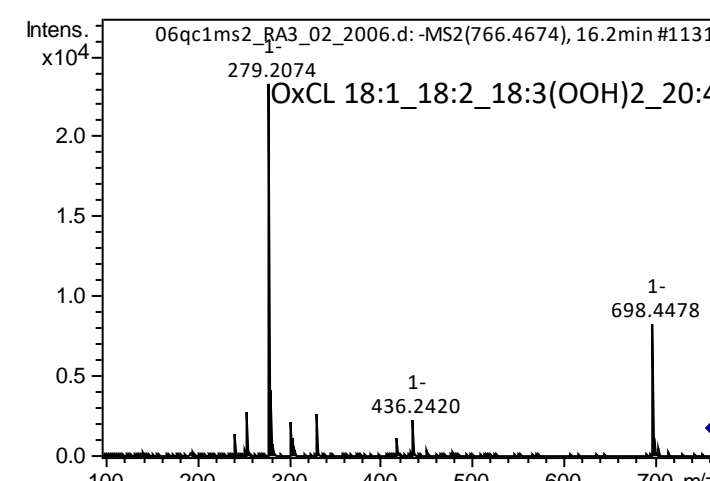

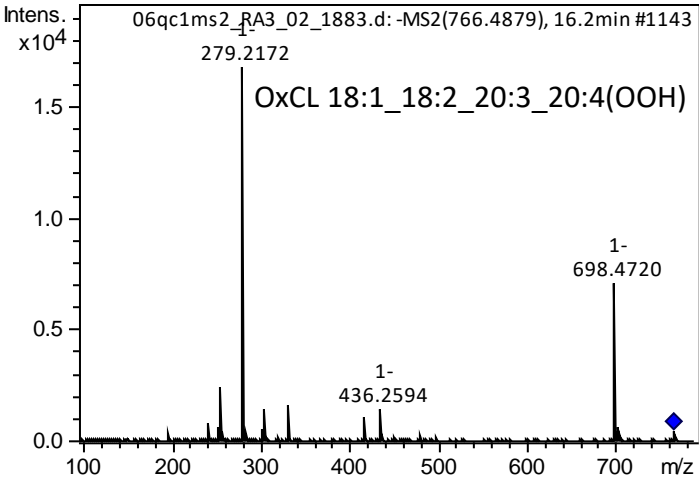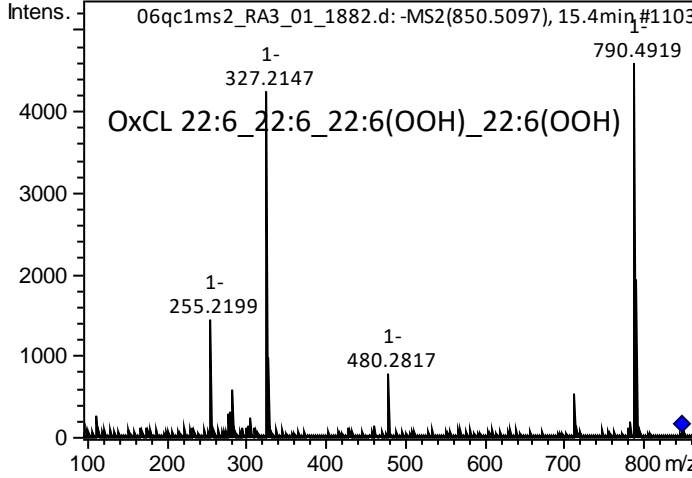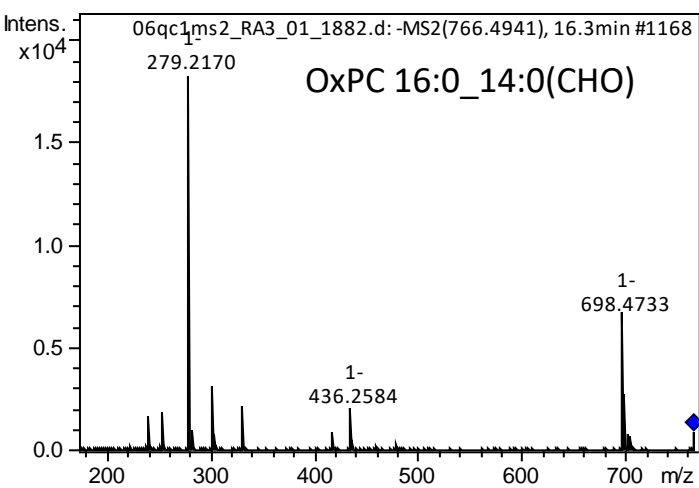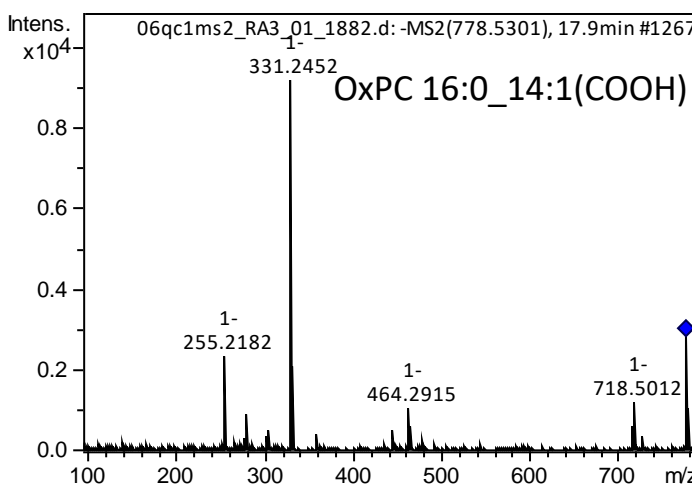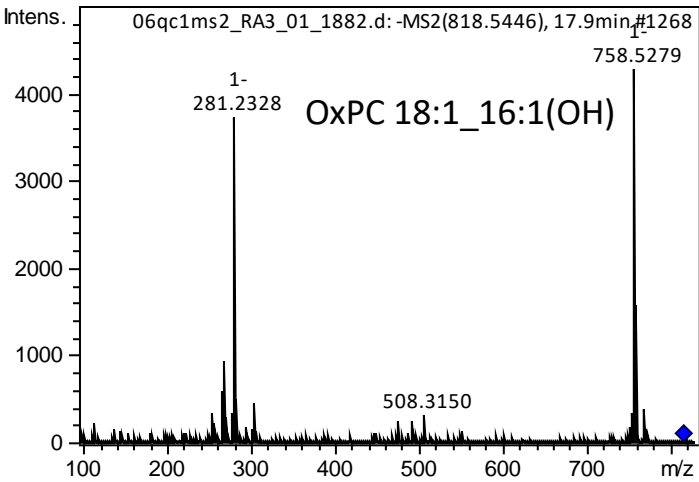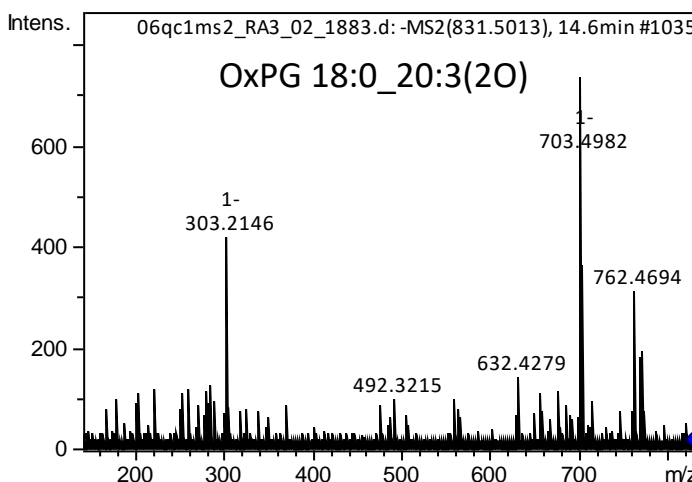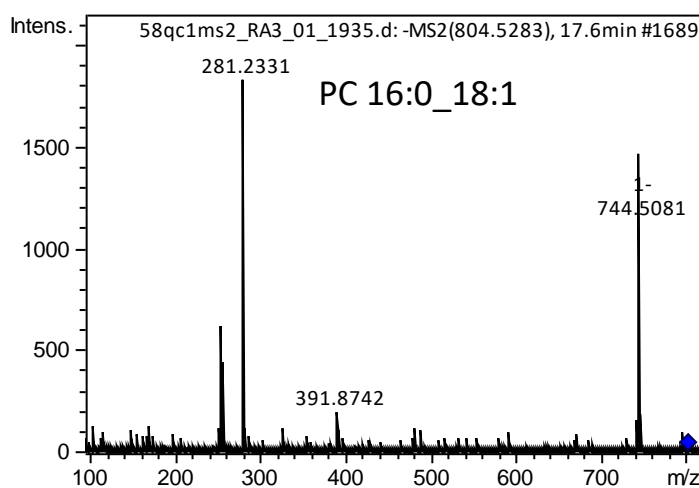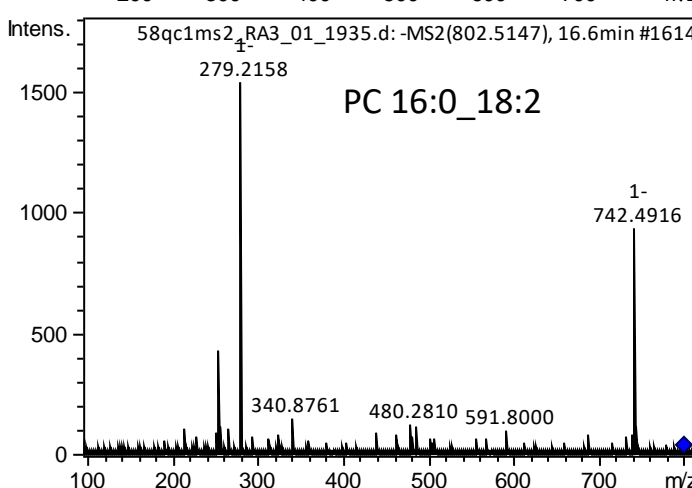

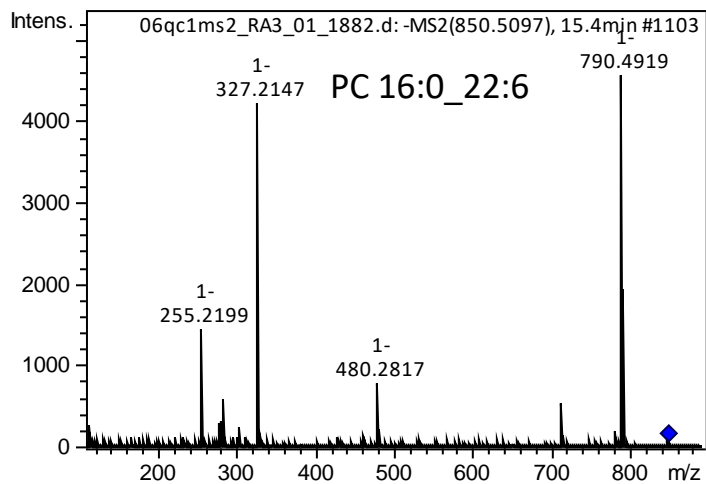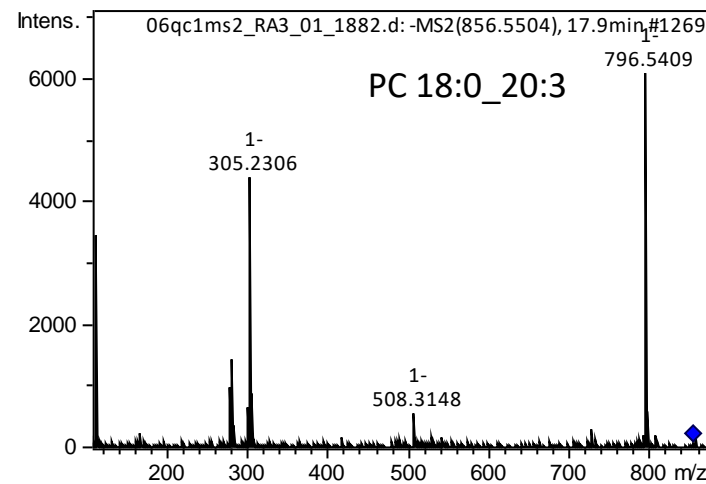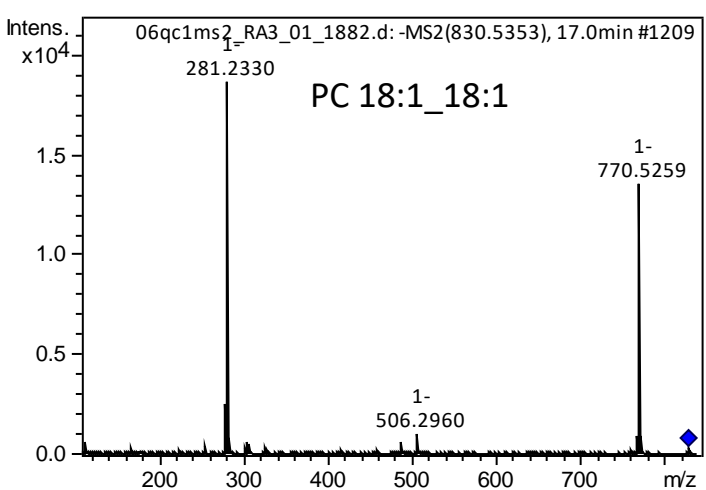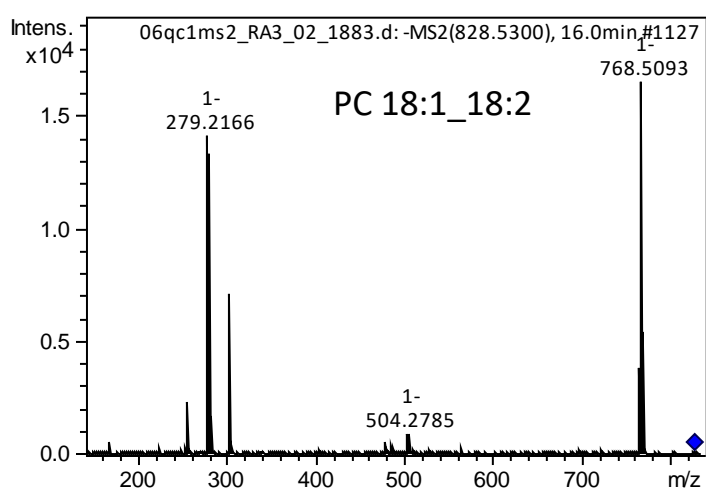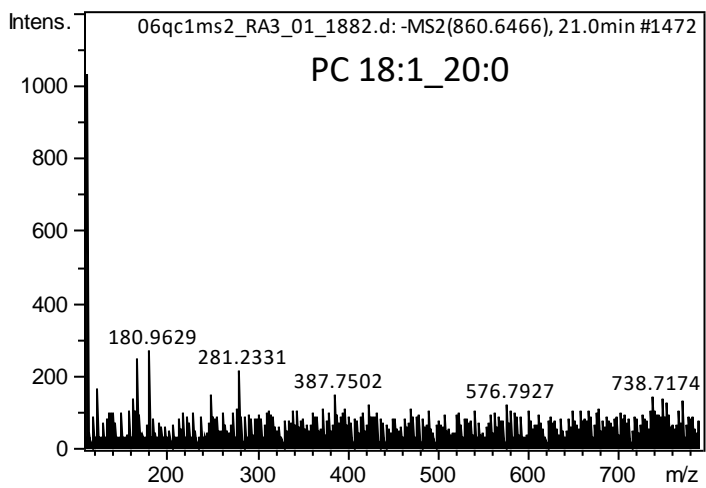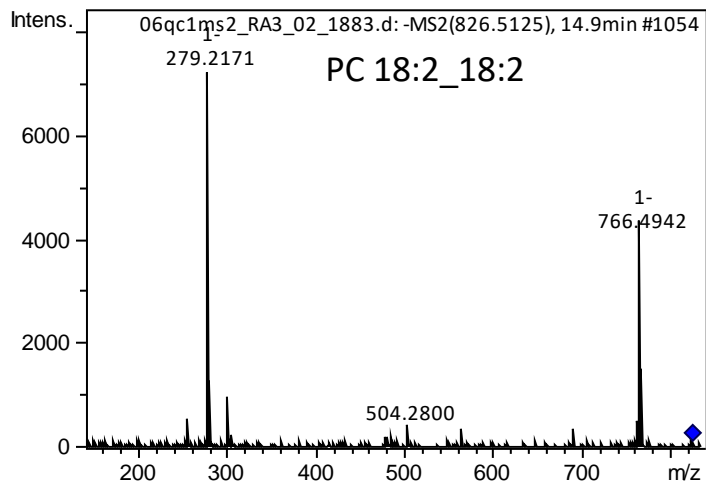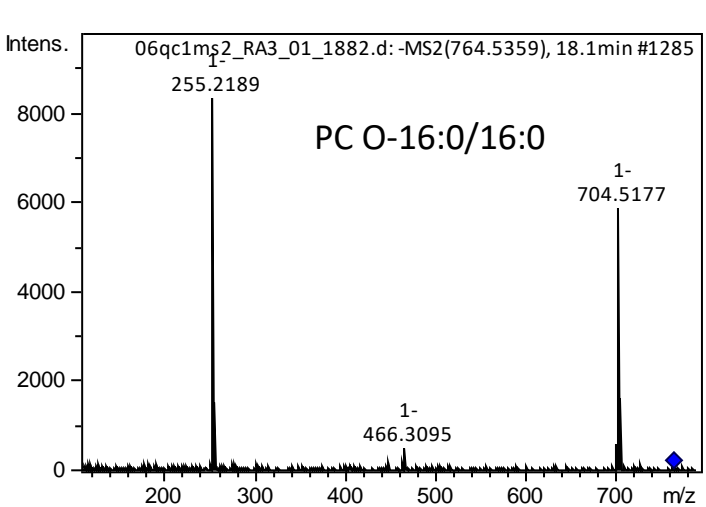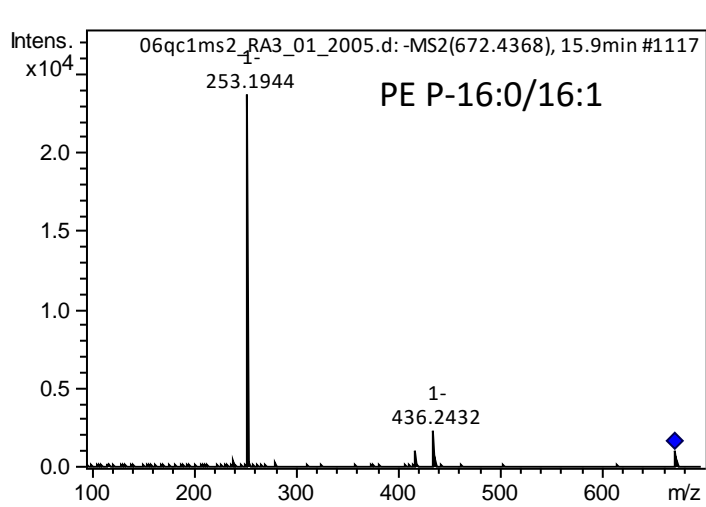

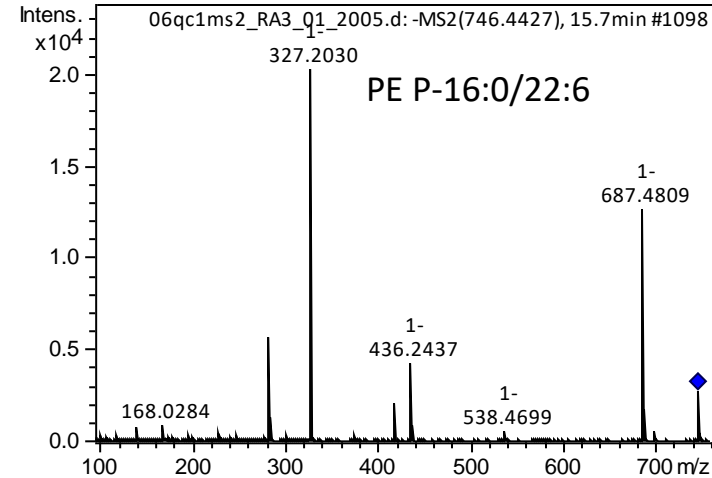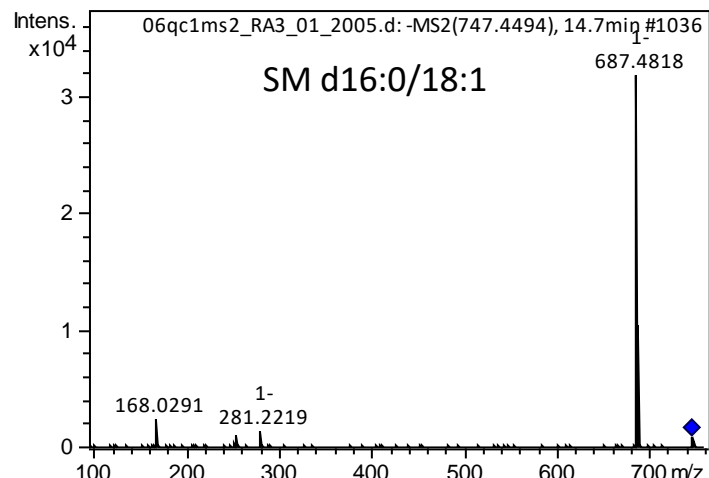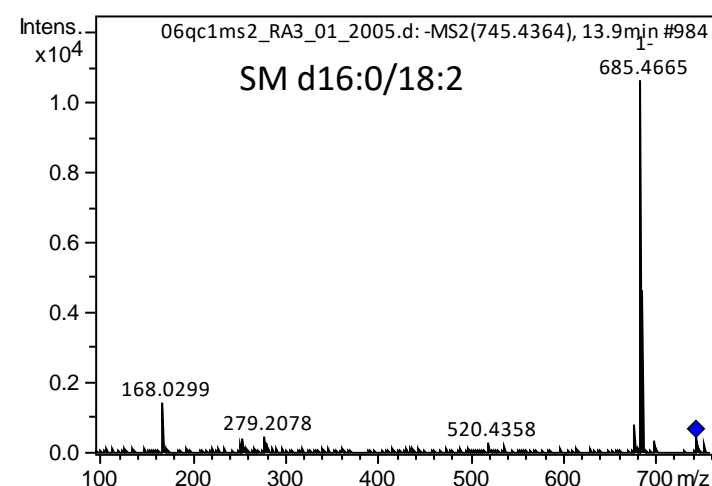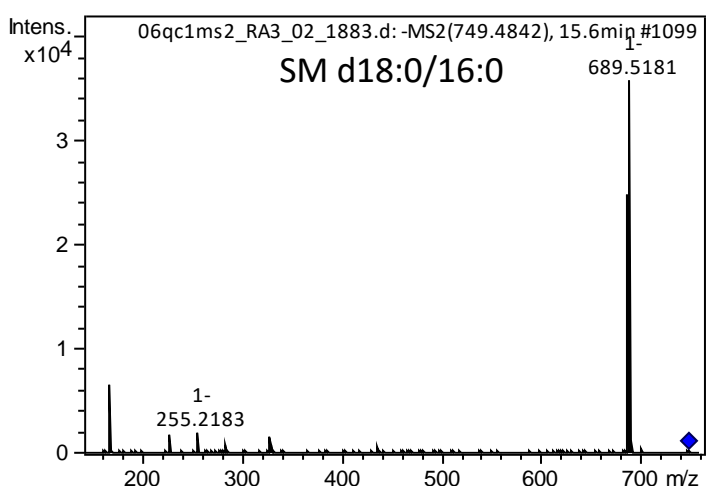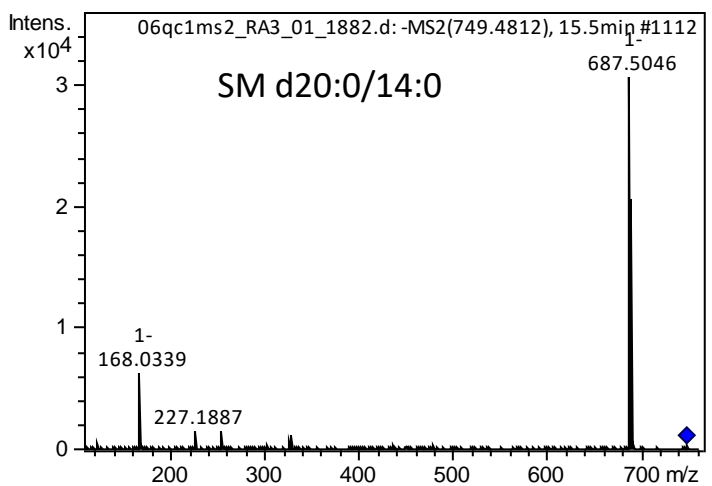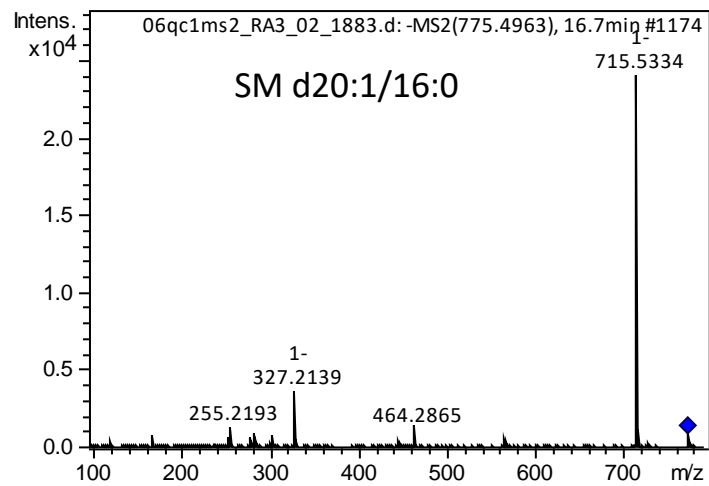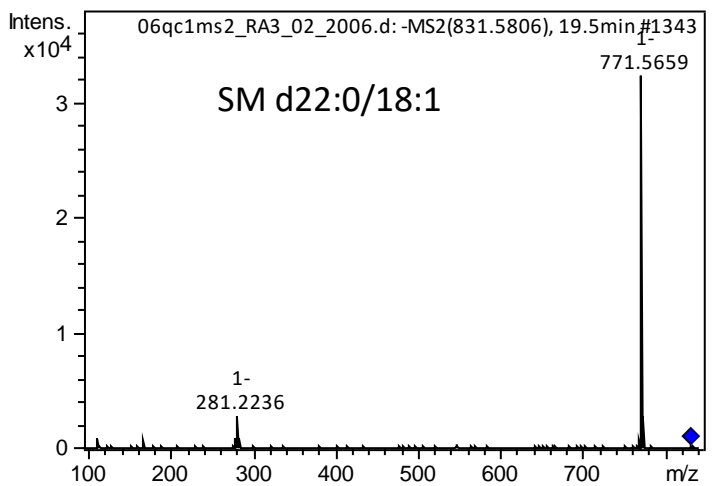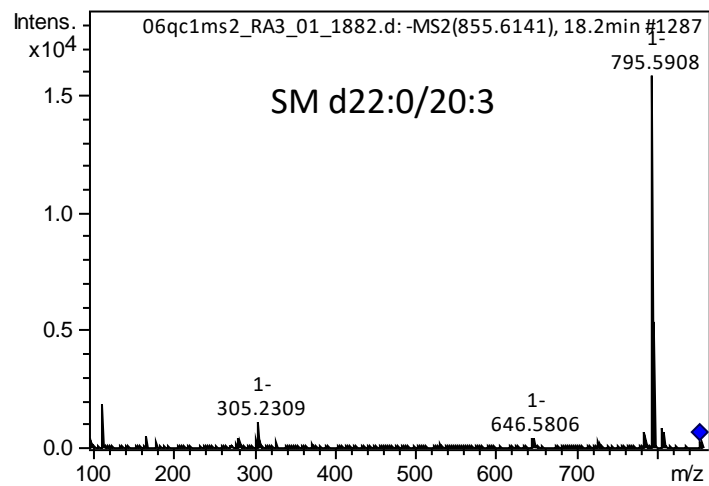

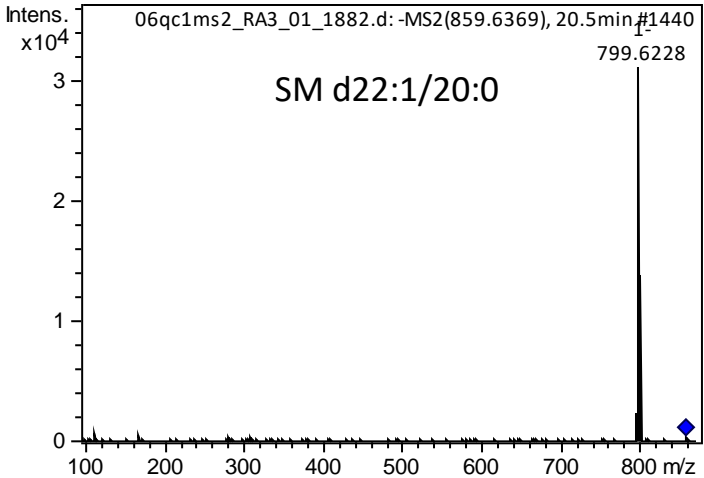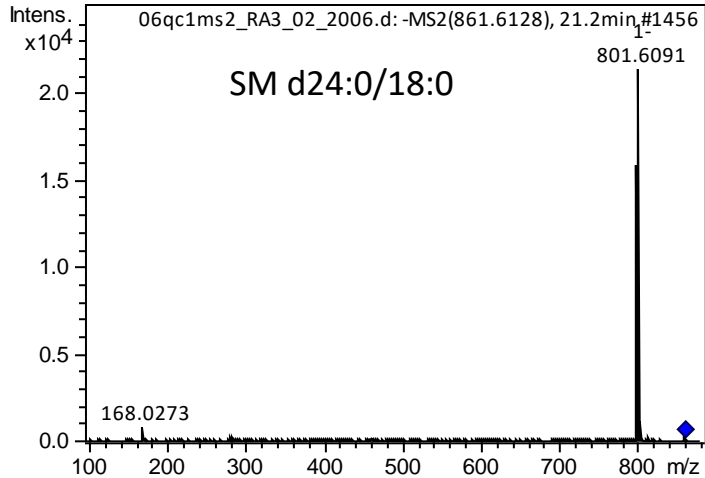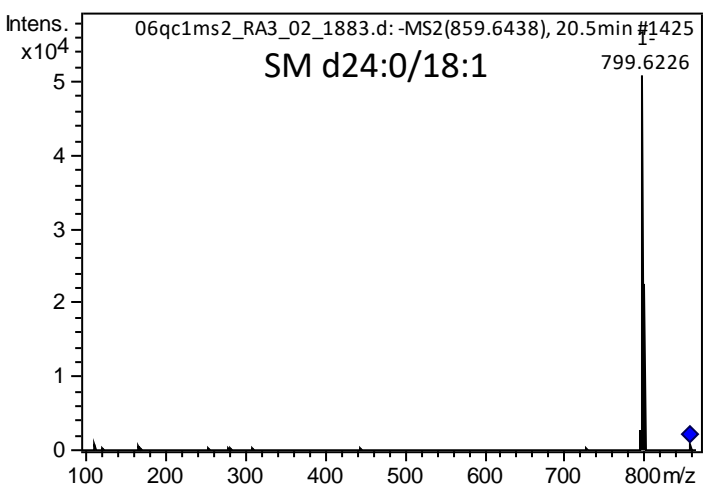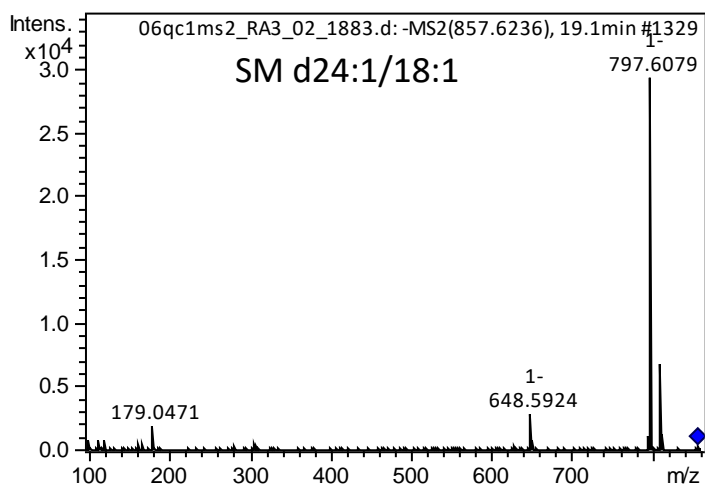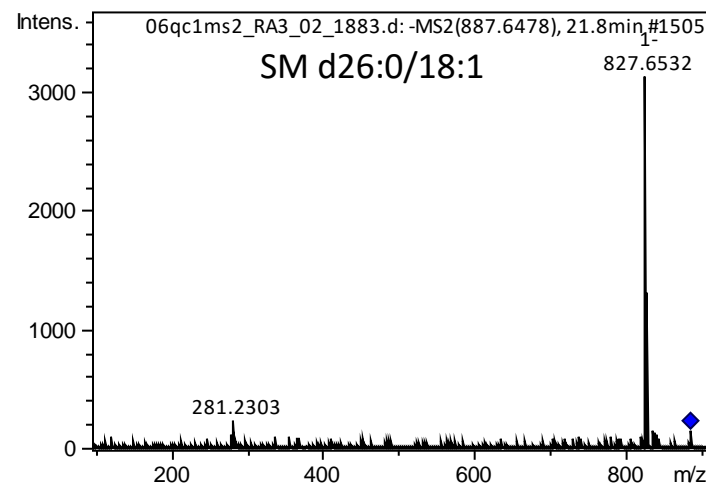

Supplement: Supplementary file 1 [file metabolites-12-00883-s001.zip › Figure S1.pdf]

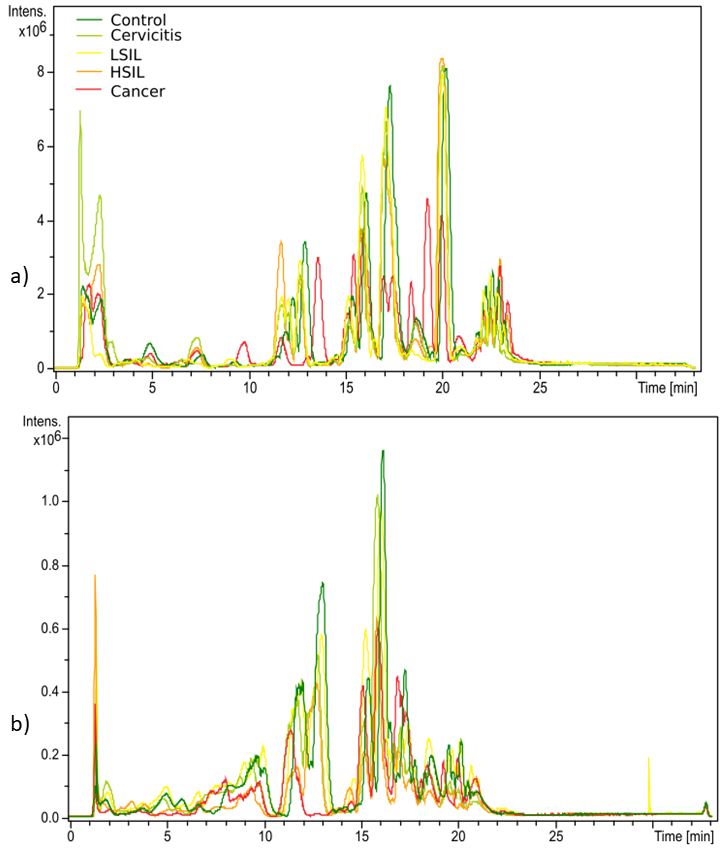

Supplement: Supplementary file 1 [file metabolites-12-00883-s001.zip › Figure S2.tif]

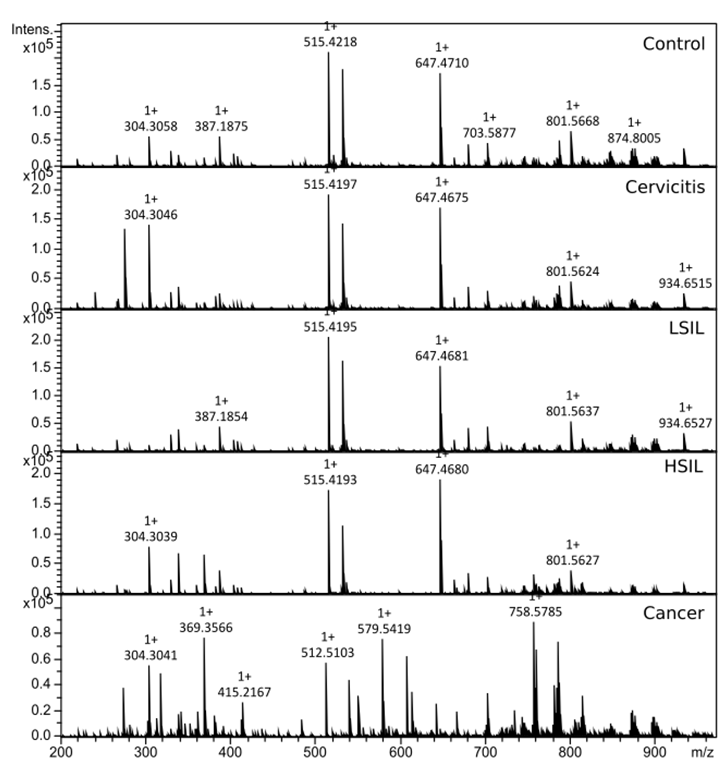

Supplement: Supplementary file 1 [file metabolites-12-00883-s001.zip › Figure S3.tif]

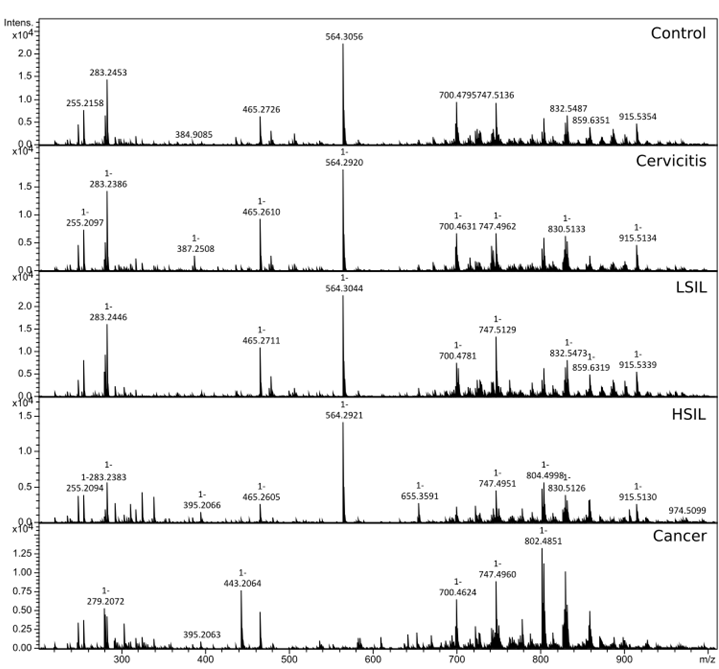

Supplement: Supplementary file 1 [file metabolites-12-00883-s001.zip › Figure S4.tif]

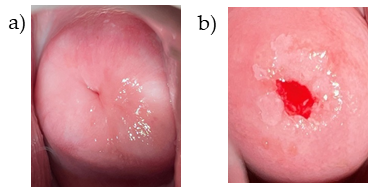

Supplement: Supplementary file 1 [file metabolites-12-00883-s001.zip › Figure S5.tif]
